# High Relative Humidity‐Induced Growth of Perovskite Nanowires from Glass toward Single‐Mode Photonic Nanolasers at Sub‐100‐nm Scale

**DOI:** 10.1002/advs.202412397

**Published:** 2024-12-12

**Authors:** Zhiqiang Wang, Xinkuo Li, Chenduan Chen, Minhan Lou, Jiajia Wu, Kai Gao, Zengling Li, Ke Sun, Zhou Li, Zhu Xiao, Linhan Li, Pan Wang, Sai Bai, Jianrong Qiu, Dezhi Tan

**Affiliations:** ^1^ Zhejiang Lab Hangzhou 311121 China; ^2^ School of Materials Science and Engineering Zhejiang University Hangzhou 310058 China; ^3^ China International Science & Technology Cooperation Base for Laser Processing Robotics Wenzhou University Wenzhou 325035 China; ^4^ School of Materials Science and Engineering Central South University Changsha Hunan 410083 China; ^5^ College of Optical Science and Engineering Zhejiang University Hangzhou 310027 China; ^6^ Institute of Fundamental and Frontier Sciences University of Electronic Science and Technology of China Chengdu 611731 China

**Keywords:** anisotropic, humidity, perovskite nanowires, single‐mode lasing, sub‐100‐nm scale

## Abstract

Metal halide perovskites (MHPs) have achieved substantial progress in their applications; however, their ionic crystal character and low formation energy result in poor structural stability and limited morphological tunability. In particular, high relative humidity (RH) commonly causes severe MHP degradation, which poses a major obstacle to long‐term device operation. Herein, high RH‐induced growth of anisotropic MHP structures on glass surfaces is reported under 25 °C and atmospheric conditions on a basis of glass corrosion by moisture. Nanowires (NWs) with tunable length and composition are obtained under 85% RH air, and water molecule‐induced facet engineering of perovskite is established for anisotropic growth. Importantly, single‐mode photonic lasing in these MHP NWs with thickness at sub‐100‐nm scale (down to 75 nm ∼ 1/7 lasing wavelength) is achieved via both one‐photon and multiphoton pumping. These nanowire lasers exhibited high quality factor (>3000), high degree of polarization (≈0.9), and excellent stability under laser irradiation. The work not only presents a distinctive technique for the growth of MHPs but also endows MHP NWs with new opportunities for nonlinear optics, strong light‐matter interactions, and active photonic integrated devices.

## Graphical abstract

High relative humidity induced glass corrosion, from which multiple ions are transported onto the glass surface to fabricate perovskite nanowires and other nanostructures. These nanowires exhibited single‐mode photonic lasing at sub‐100‐nm scale.

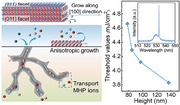

## 1 Introduction

MHPs are fascinating semiconducting materials with attractive optoelectronic properties and great potential for various applications in solar cells, light‐emitting diodes, lasers, and photodetectors.^[^ ^1^, ^2^, ^3^, ^4^ ^]^ Unfortunately, MHPs show low structural stability and are easily attached by water and oxygen.^[^ ^5^, ^6^ ^]^ In particular, humidity‐induced structural degradation is a major issue for the long‐term stability of MHPs for commercial applications.^[^ ^7^, ^8^, ^9^, ^10^ ^]^ Consequently, a high RH is an adverse environment in perovskite chemistry and devices. Despite studied, the mechanism of the interaction between water molecules and MHPs and the role of humidity in the growth of MHPs are unknown.^[^ ^1^, ^5^, ^7^, ^11^, ^12^ ^]^

An anisotropic morphology endows MHPs with distinctive properties. For example, MHP nanowires (NWs) contain unique 1D confined photonics and carriers for polarized optoelectronic devices.^[^ ^13^, ^14^, ^15^ ^]^ However, the intrinsic ionic crystal character and structural symmetry of the perovskites enable a rapid crystallization process, and the control of anisotropic growth is challenging.^[^ ^16^, ^17^ ^]^ Solution‐phase and vapor‐phase methods allow the synthesis of various functional NWs.^[^ ^18^, ^19^, ^20^ ^]^ Organic molecules are essential in solution‐phase synthesis for the anisotropic growth and assembly of MHPs. The stability and controllability of NWs for solid state applications are limited by ligand dissociation and aggregation.^[^ ^16^, ^21^ ^]^ High temperature is necessary for vapor‐phase synthesis, such as vapor–liquid–solid growth and vapor epitaxial growth, which are applicable to synthesis of most nanostructures, including MHPs.^[^ ^16^, ^18^, ^19^, ^20^, ^22^, ^23^ ^]^ However, high temperature could be detrimental to MHPs. Furthermore, the growth processes are complicated by the necessity of oxygen‐ and water‐free environments.

Additionally, single‐mode lasing, which can be from NWs, are important for numerous applications including lighting, optical communication and computing.^[^ ^13^, ^24^, ^25^ ^]^ There is a continuous effort to reduce the diameter of NW lasers to a subwavelength scale for the miniaturized coherent light source and the emerging photonic and optoelectronic integrated devices.^[^ ^26^, ^27^, ^28^, ^29^, ^30^ ^]^ One typical method to realize lasing at subwavelength scale is using surface plasmon to tightly confine photons in a hybrid system (called plasmonic laser) consisting of NWs and metals.^[^ ^26^, ^27^, ^29^, ^31^ ^]^ To achieve lasing, the diameters of organic–inorganic hybrid and all‐inorganic perovskites are suggested to be >≈160 and ≈180 nm, respectively.^[^ ^13^, ^32^ ^]^ In fact, the reported MHP micro/nanowire lasers have diameters of several hundred nanometers that approximately equal to lasing wavelength or micrometers, which limits applications in ultracompact integration.^[^ ^13^, ^17^, ^23^, ^32^, ^33^ ^]^ To date, there is also lack of methods to synthesize NWs with suitable size below 100 nm for nanolasers.^[^ ^34^ ^]^ Moreover, there is a growing interest in multiphoton pumped (MPP) laser with efficient frequency upconversion of coherent light, which is crucial for nonlinear optics, near‐infrared communications and bioimaging.^[^ ^33^, ^35^, ^36^, ^37^, ^38^, ^39^ ^]^ Unfortunately, to achieve MPP lasers at subwavelength scale is more challenging due to much smaller cross‐section of multiphoton absorption (in particular, three and more photon absorption) and larger *P*_th_ than that of one‐photon process. MPP single‐mode lasers have not been reported in NWs.

We report humidity‐induced room temperature (25 °C) growth of MHP NWs on glass surfaces under 85% RH atmospheric conditions based on precursor glass corrosion by moisture. We established a principle of water molecule‐induced facet engineering for the anisotropic growth of MHPs in that the mechanisms were clarified. These MHPs showed tunable composition and shape as well as PL. Additionally, MHP NWs showed single‐mode lasing upon one or multiphoton pumping with high quality factor, high polarization dependence and excellent stability. More importantly, single‐mode lasing in a MHP NW with the thickness as small as 75 nm was achieved and demonstrated a lasing threshold dependence on thickness of NWs.

## 2 Results and Discussion

### 2.1 Fabrication and Optical Properties of CsPbBr_3_ NWs

**Figure 1:**
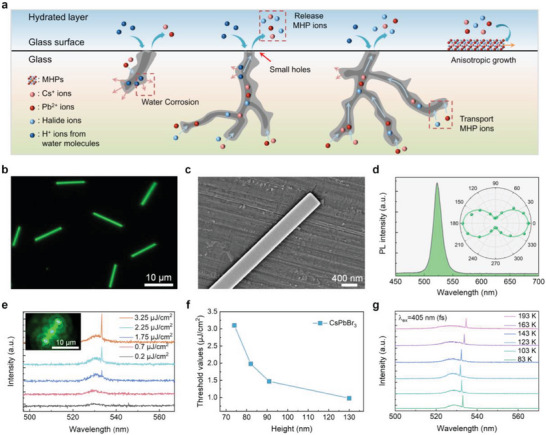
Optical properties of CsPbBr_3_ NWs. a) Schematic of humidity‐induced synthesis of CsPbBr_3_ NWs on glass. b) PL image. c) SEM image. d) PL spectrum. Excitation: 405 nm. Inset of d) Polar plots of intensity of polarization‐dependent PL. The green curve in the polar plots shows the fitting result with a cosine function. e) Lasing spectra excited by a 405 nm femtosecond laser with various pump fluences at 83 K. Inset: Lasing image. f) *P*_th_ as a function of the height of CsPbBr_3_ NWs excited by a 405‐nm femtosecond laser. g) Lasing spectra as a function of temperature.

Humidity induces glass corrosion via hydration and ion release/exchange, which allows outward diffusion of mobile ions to the surface to produce an interfacial hydrated layer.^[^ ^40^, ^41^, ^42^ ^]^ Cs^+^, Pb^2+^, and halide ions exhibit high mobility and enable supersaturation in the hydrated layer for the formation of MHPs (**Figure** **1**a).^[^ ^40^, ^41^, ^42^, ^43^ ^]^ We obtained NWs with the length of ≈10 µm (Figure 1b; Figure S1, Supporting Information) on the surface of Cs^+^, Pb^2+^, and Br^−^ doped glass after exposure to 85% RH air for 12 h. The hydrated layer on glass surface was clearly observed via SEM microscopy, which provided a good platform for the formation of perovskite NWs (Figure S2, Supporting Information). The NWs featured smooth surface (Figure 1c) and a rectangular cross‐section (Figure S3, Supporting Information), which were confirmed by atomic force microscopy (Figure S4, Supporting Information, revealing a height of 84.5 nm). The photoluminescence (PL) peak centered at 524 nm (Figure 1d) indicated the presence of CsPbBr_3_ crystals, verified by X‐ray diffraction (Figure S5a, Supporting Information), Raman spectroscopy (Figure S5b, Supporting Information), and element mapping (Figure S6, Supporting Information). The full width at half maximum (FWHM) of the PL spectrum was as small as 13.8 nm, suggesting the high‐quality of CsPbBr_3_ NWs.^[^ ^13^, ^44^ ^]^ The 1D morphology endows MHP NWs with remarkable properties. The PL of CsPbBr_3_ NW is highly polarization‐dependent (inset of Figure 1d) due to the dielectric mismatch between NWs and the surrounding environment.^[^ ^14^, ^34^ ^]^ The degree of polarization (DOP) of PL for CsPbBr_3_ NWs was determined to be 0.78 (Table S1, Supporting Information) using the following equation: DOP = (*I*_A_ − *I*_P_)/(*I*_A_ + *I*_P_), where *I*_A_ and *I*_P_ are the PL intensity with the polarization of the excitation laser along and perpendicular to the NW, respectively. The obtained DOP is among the highest values for MHP NWs (Table S2, Supporting Information).^[^ ^13^, ^14^, ^34^ ^]^ The as‐synthesized CsPbBr_3_ NWs also show superior light guiding performance with a low propagation loss of 0.018 dB µm^−1^ (Figure S7 and Table S3, Supporting Information).^[^ ^45^, ^46^ ^]^

The high crystal quality, smooth surface, well‐defined morphology, and low light propagation loss enable MHP NWs an excellent gain medium and Fabry–Perot microcavity for nanolasers.^[^ ^13^ ^]^ We observed lasing in a MHP NW (inset of Figure 1e) when the pumping fluence of the 405 nm femtosecond laser was above the threshold. Figure 1e revealed that there was only spontaneous emission when the pumping fluence was low (e.g., 0.7 µJ cm^−2^) and further increasing the pumping fluence to above the threshold led to single‐mode lasing (SML), whose intensity increased fast with pumping fluence (Figure S8a, Supporting Information). The lasing spectrum was centered at 533.4 nm with a FWHM (∆*λ*) of 0.16 nm (Figure S8b, Supporting Information) and the quality factor (Q) was determined to be 3334 following the formula: Q = *λ*/∆*λ*, where *λ* is lasing wavelength. *P*_th_ increased with decrease of height and the smallest height for single‐mode laser was 75 nm (≈1/7*λ*) with a pumping threshold of 3.1 µJ cm^−2^ (Figure 1f), which is comparable to or smaller than that of larger‐sized NW and microwire lasers.^[^ ^32^, ^33^, ^35^, ^47^ ^]^ This implies the high quality of the as‐prepared perovskite nanowires and low loss propagation loss in lasing output. To our knowledge, this is the first time to achieve single‐mode photonic nanolaser in a MHP NW at sub‐100‐nm scale, breaking down the optical diffraction limit. The negligible blueshift with increase of pumping fluence (Figure 1e) indicated that the low‐threshold lasing in MHP NWs at sub‐100‐nm scale originated from the exciton‐photon coupling, rather than exciton‐polaritons or electron–hole plasma, and provided a stable lasing platform for real applications.^[^ ^13^, ^30^, ^47^, ^48^ ^]^ Figure 1g illustrated that SML still occurred when the temperature was up to 193 K. The lasing peak showed a blue shift with the temperature increasing from 83 to 123 K and then redshift with the temperature was up to 193 K, which was attributed to the synergistic effects between lattice thermal expansion and exciton‐phonon interaction, supported by the analysis of data extracted from Figure 1g (Figure S9, Supporting Information).^[^ ^49^ ^]^ With the increase of temperature from 83 to 123 K, a blue shift of lasing peaks was observed (Figure S9a, Supporting Information), in consistent with that of PL peaks, implying the key role of lattice thermal expansion effect. However, in the range of 123–193 K, a red shift tendency of lasing peaks with the increase of temperature was observed and furthermore, the energy difference between PL peaks and lasing peaks becomes larger as the increase of temperature (Figure S9, Supporting Information), being ascribed to the dominant role of electron‐phonon interaction in this temperature range.

The size of our photonic NW laser is nearly the same with that of plasmonic lasers, but *P*_th_ is one to four orders of magnitude smaller and Q is several times larger.^[^ ^25^, ^26^, ^27^, ^29^ ^]^ Ultrasmall size, large Q and low power consumption are essential for on‐chip integrated applications. For the butt‐coupling (Figure S10, Supporting Information), the present NW laser has an ultrasmall area of 0.025 µm2, which is one order of magnitude smaller than previously demonstrated single‐mode photonic micro/nanowire lasers (Table S4, Supporting Information).^[^ ^33^, ^47^ ^]^

We also analyzed the polarization properties of lasing. A high DOP of 0.93 was obtained (Figure S11a, Supporting Information). In this case, the lasing intensity reached the highest with the polarization of the electric field (E_y_) perpendicular to the NWs. We simulated the waveguide mode in NWs on glass (Figure S11b, Supporting Information) and confirmed that the fundamental transverse electric (TE) mode perpendicular to the NWs can be well supported in a NW with a width of 320 nm and a height of 80 nm (Figure S11c, Supporting Information). However, other modes were not supported (Figure S11d, Supporting Information), which is in line with the highly polarization‐dependent lasing. The simulation results also validate the feasibility of lasing in perovskite nanowires with small thickness at sub‐100‐nm scale. To meet the end of further reducing the NW size while maintaining lasing emission, two ways might be useful. The first one is to prepare nanowires with higher quality, for example, more regular shape and smooth surface with less structure defects. This will reduce the optical propagation loss to an extent. Another one is the utilization of surface plasmons to localize light and realize lasing beyond the size limitation.

**Figure 2:**
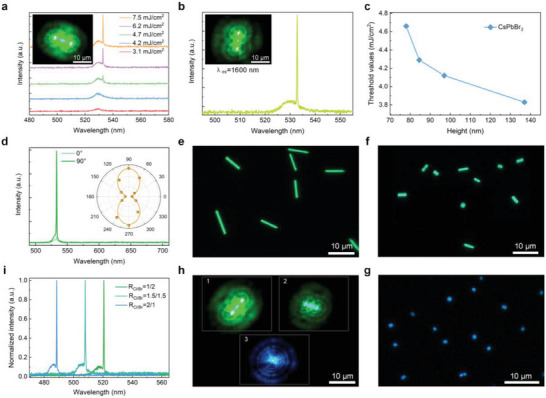
MPP lasing of MHPs. a) Lasing spectra as a function of pumping fluence of a 1030‐nm femtosecond laser for a CsPbBr_3_ NW. b) SML under a 1600 nm femtosecond laser pumping of a CsPbBr_3_ NW. Insets: Lasing images. c) *P*_th_ as a function of height of CsPbBr_3_ NWs excited by a 1030‐nm femtosecond laser. d) Lasing spectra at two different polarization angle (0° and 90°). Inset: polar plots of polarization dependent lasing intensity. e–g) PL images. h) Lasing images. e,h_1_) *R*_Cl/Br_ = 1/2. f,h_2_) *R*_Cl/Br_ = 1.5/1.5. g,h_3_) *R*_Cl/Br_ = 2/1. i) Lasing spectra of mixed halide perovskites.

SML was also achieved in a NW with multiphoton pumping when excited using a 1030 nm (**Figure** **2**a) and 1600 nm (Figure 2b) femtosecond laser, which originated from three‐photon (Figure S12a,b, Supporting Information) and four‐photon pumped stimulated emission, respectively. *P*_th_ increased when the height of NWs decreased (Figure 2c). The SML was observable in a NW with the height down to 78 nm and the *P*_th_ was 4.65 mJ cm^−2^. Q was calculated to be 3481 with FWHM of 0.15 nm (Figure S12c, Supporting Information). The side mode was considerably suppressed with a large ratio of side mode suppression (*R*_SMS_) of 12 dB calculated using the formula of *R*_SMS_ = 10log_10_(*I*_d_/*I*_s_) (Figure S12d, Supporting Information), where *I*_d_ and *I*_s_ are the intensity of the dominant mode and side mode, respectively. MPP SML was available by using 1200 nm (three‐photon pumping, Figure S13, Supporting Information) and 2000 nm (four‐photon pumping, Figure S14, Supporting Information) femtosecond laser as the excitation source. MPP sing‐mode lasing was observable when the temperature reached 173 K (Figure S15, Supporting Information). The lasing intensity is polarization dependent with a high DOP of 0.88 (Figure 2d; Table S5, Supporting Information). The polarization‐dependence indicates to a new usable dimensionality for integrated photonics, nonlinear optics, light sources, and communications with MHP NWs.^[^ ^50^, ^51^ ^]^

### 2.2 Optical Properties of As‐Prepared Mixed‐Halide Perovskites

Mixed‐halide perovskite NWs were formed on Cl^−^‐Br^−^ codoped glass with Cl^−^/Br^−^ (*R*_Cl/Br_) doping ratios of 1/2 (Figure 2e) and 1.5/1.5 (Figure 2f) using the same humidity treatment strategy. MHP microplates were produced when *R*_Cl/Br_ = 2/1 (Figure 2g; Figure S16, Supporting Information). The PL was centered at 512, 502, and 484 nm for *R*_Cl/Br_ = 1/2, 1.5/1.5, and 2/1, respectively (Figure S17, Supporting Information), which was highly polarized (Figure S18, Supporting Information). These nanostructures of mixed‐halide perovskites also exhibited good PL waveguide behaviors (Figure S19, Supporting Information). SML with tunable color (Figure 2h) and wavelength (Figure 2i) was demonstrated in these MHP nanostructures with small FWHM (≈0.15 nm, and Table S5, Supporting Information), high Q (>3000, Table S5, Supporting Information), high DOP (>0.8, Figure S20, Supporting Information), low 3PP threshold (Figure S21, Supporting Information), and high *R*_SMS_ (Table S5, Supporting Information). We also realized SML in mixed‐halide perovskites at sub‐100‐nm scale and an increase in *P*_th_ with decrease of height was also observed in mixed‐halide perovskite NWs at sub‐100‐nm scale (Figure S22, Supporting Information). It is worth to note that the lasing in MHP microplatelets with *R*_Cl/Br_ = 2/1 was formed by whispering‐gallery mode and the lateral size of lasers can be <1 µm (Figure 2g). Although different lasing characteristics have been detected on the mixed‐halide perovskite NWs, it is difficult to make an effective comparison based on different Cl/Br ratios due to the multivariate situation. Five‐photon pumped SML was observed in MHP microplatelets when excited by a 2000 nm femtosecond laser (Figure S23, Supporting Information).

### 2.3 Growth Mechanism of Humidity‐Directed MHPs from Glass

Although trace water has been reported to be able to accelerate the crystallization of perovskites, the intrinsic role played by water molecules is mass transportation via dissolving the precursors or partial defective perovskites, and the final perovskites are formed in the local water‐free environment.^[^ ^11^, ^52^ ^]^ Particularly, high humidity (>50%) generally induces significant degradation of perovskites.^[^ ^7^, ^8^, ^11^ ^]^

**Figure 3:**
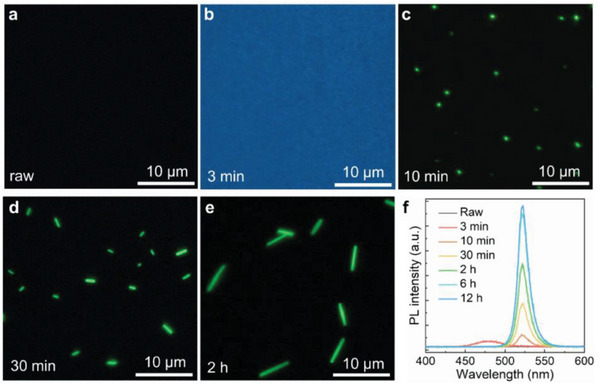
Evolution of CsPbBr_3_ NWs with an increase in the humidity treatment time. a–e) PL images of initial glass and CsPbBr_3_ structures with an increase in growth time (85% RH). f) PL spectra of CsPbBr_3_ structures with an increase in growth time (85% RH).

In this study, water molecules can adsorb onto and interact with glass surface, and induce corrosion via hydration and ion release/exchange, forming a hydrated layer (Figure 1a; Figure S2, Supporting Information), which was confirmed by the presence of small holes on the surface under high RH (Figure S24, Supporting Information). The initial glass does not exhibit PL emission (**Figure** **3**a). After 3 min under 85%RH, we observed blue emission (Figure 3b) from the glass surface with the PL peak at 480 nm (Figure 3f), which indicates emergence of CsPbBr_3_ quantum dots (QDs) with the size of ≈4.2 nm.^[^ ^21^ ^]^ Green emissive particles appeared on the surface after 10 min with PL at 523 nm (Figure 3c), which grew into short NWs after 30 min (Figure 3d). The length of NWs increased with time and reached ≈10 micrometers after 12 h. The length of NWs was adjustable by controlling the humidity treatment time (Figure 3), which could reach over 40 µm (Figure S25, Supporting Information). Length of mixed‐halide MHP NWs also increased with growth time (Figure S26, Supporting Information). Although the length increased, the PL peak kept constant after 2 h (Figure 3f). In situ experiments confirmed the growth of NWs during humidity treatment (Figure S27, Supporting Information). To exclude the effect of the oxygen in the air, we also created CsPbBr_3_ NWs under 85% RH nitrogen conditions (Figure S28, Supporting Information).

**Figure 4:**
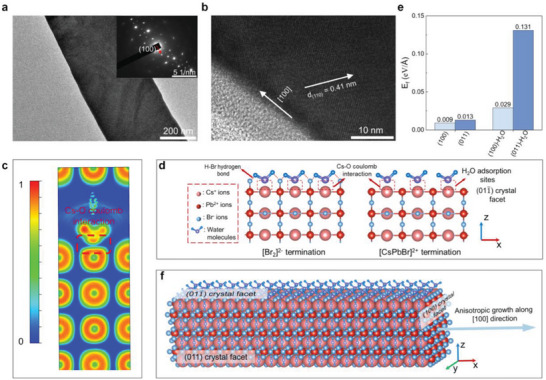
Growth mechanism of NWs. a,b) TEM results of CsPbBr_3_ NWs grown on the glass surface under 85% RH air. a) TEM image. Inset: selected‐area electron diffraction pattern. b) High‐resolution TEM image of a CsPbBr_3_ NW. The selected‐area electron diffraction pattern showed that CsPbBr_3_ growth occurred along the [100] direction, confirmed by high‐resolution TEM images with lattice distance of 0.41 nm indexed to the (110) plane of a cubic CsPbBr_3_ crystal. The four‐fold symmetrical SAED pattern was also aligned with a cubic crystal structure. c) Electron localization related to Cs─O bond. d) Water adsorption on the (011) facet. e) Facet energy before and after water adsorption. f) Schematic of anisotropic growth of MHPs along the [100] direction.

Next, we discussed the growth mechanism of high‐quality NWs. Blue PL at 480 nm was detectable in 3 min after exposure glass to 85% RH air, because of the presence of ≈4.2 nm CsPbBr_3_ quantum dots (QDs) (Figure 3), which were embedded in the glass beneath the surface in the range of 0–4 µm (Figure S29, Supporting Information). Increasing the interaction time led to spread out of active ions, including Cs^+^, Pb^2+^, and halide ions, for CsPbBr_3_ growth along the [100] direction on glass surface (**Figure** **4**a,b).^[^ ^40^, ^41^, ^43^ ^]^ Owing to the structure symmetry, the ionic crystal characteristics, and the close facet energy between (100) and (011) facets (Figure 4e), 1D growth of MHPs (cubic phase in particular) is unfavorable, and epitaxial substrates or surface ligands were usually required.^[^ ^22^, ^53^, ^54^ ^]^ Electron localization related to Cs─O was observed based on the density function theory calculation (Figure 4c), suggesting the adsorption of water molecules on Cs^+^ in the perovskite crystal via the Coulomb interaction (Figure 4d), which would prevent [PbBr_6_]^2−^ octahedra from further adsorbing on the perovskite crystal surface and subsequent crystal growth. Furthermore, the (100) facet is nonpolar, whereas the (011) facet is polar with positively or negatively charged terminals and could be attacked easily by polar water molecules through Cs─O bonding and hydrogen bonding (Figure 4c,d).^[^ ^55^ ^]^ This adsorption could also lead to a significantly increased difference in facet energies of the (100) and (011) facets (Figure 4e). These factors facilitated 1D growth of CsPbBr_3_ along the [100] direction (Figure 4f). Simultaneously, the preformed QDs were degraded, as indicated by disappearance of blue emission, to provide high‐purity precursors for growth of NWs. Crystallization process of MHPs was also modulated by removing defective sites in MHPs to improve crystal quality.^[^ ^43^, ^52^, ^56^ ^]^ These contribute to the high crystal quality and smooth surface (Figure 1c; Figure S30, Supporting Information) of NWs, which are essential for lasing at sub‐100‐nm.

Anisotropic growth was influenced by the supersaturation condition that could be enhanced by low supersaturation.^[^ ^13^, ^22^ ^]^ Taking the Cl^−^‐Br^−^ codoped glass as an example, Cl^−^ diffused faster than Br^−^ because of its smaller radius and weight. A larger *R*_Cl/Br_ in glass could result in higher supersaturation. The presence of Cl^−^ decreased the formation energy of perovskites and increased the rate of nucleation and crystal growth.^[^ ^57^, ^58^ ^]^ Consequently, the length of CsPb(Cl*_x_* Br_1−_ *_x_*)_3_ NWs decreased as the Cl^−^ concentration increased, and microplatelets were formed with *R*_Cl/Br_ = 2/1 (Figure 2g).

Interestingly, we found that suitable RH is important to fabricate high‐quality NWs. No MHPs were formed at 20% RH. PL was detectable after treatment at 35% RH (Figure S31, Supporting Information) and 45% RH (Figure S32, Supporting Information), but no isolated particles were observed on the surface even after 21 days (Figure S33, Supporting Information). NWs emerged when these glasses were further treated at 85% RH for 1 day (Figure S34, Supporting Information). CsPbBr_3_ and CsPb(Cl*_x_* Br_1−_ *_x_*)_3_ MHPs tetrahedrons were obtained at 65% RH (Figure S35, Supporting Information) and 75% RH (Figure S36, Supporting Information). The height of the interfacial hydrated layer decreased exponentially with RH at levels such as 65% RH and 75% RH, potentially resulting in a larger supersaturation compared to 85% RH. This phenomenon partly explains the formation of MHPs tetrahedrons.^[^ ^13^, ^22^, ^59^, ^60^ ^]^ At the RH level of 95%RH, some dirty and un‐defined structures formed on the glass surface instead of perovskite nanowires (Figure S37, Supporting Information), which might be originated from the simultaneous formation and damage of perovskites under such a high level of RH. Based on the experimental results from such a broad range of humidity conditions, we conclude that the humidity level of 85%RH would be the optimal growth environment for perovskite nanowires.

In solution‐phase and vapor‐phase synthesis methods, organic molecules and high temperature are usually essential.^[^ ^16^, ^18^, ^20^, ^61^ ^]^ Therein, the stability and controllability of NWs for solid‐state applications are limited by ligand dissociation and aggregation. Careful temperature control was also required in the traditional vapor‐phase synthesis.^[^ ^43^, ^56^ ^]^ Consequently, defects and inhomogeneity could be created in NWs during the synthesis or deposition process, which may hamper their optical performance, for example, increasing the lasing pumping threshold and decreasing the damage threshold. Our work demonstrates an alternative mechanism to synthesize MHPs under moderate basic environment that the hydrated layer exists on the glass surface, in contrast to the previous reports that high centration halide ions were used to stabilize perovskite crystals in the acid solution by diminishing the solubility and grow 3D perovskites in the oxygen‐free aqueous solution.^[^ ^43^, ^58^, ^62^ ^]^

Glass is a precursor for formation of MHPs in our study that relies on the interaction between water molecules and glass at high RH. NWs were obtained after exposing the glass to humid air on a damp day without controlling RH (Figure S38, Supporting Information), which promised to produce MHP NWs outside the laboratory for lighting, detection, and energy conversion without the need of complicated chemicals or heavy experimental facilities. Furthermore, the on‐substrate synthesis is free of surfactant, aggregation, and fusion, and the glass can serve as a facile dielectric substrate, which is of primary importance for fabricating opto‐electronic devices and stabilizing the optical properties of MHPs for multifunctional applications.^[^ ^21^, ^63^ ^]^ We even could tune the morphology of the MHPs to fabricate stable MHP tetrahedrons (Figures S35,S36, and S39, Supporting Information).

In addition, the developed technique is also applicable to optical data storage and information encryption. A nonluminescent “2024” pattern was created by femtosecond laser direct writing on the glass surface and humidity treatment resulted in green emission (Figure S40a, Supporting Information).^[^ ^64^ ^]^ A secondary writing transformed “2024” to “8888” (Figure S40b, Supporting Information). Thus, we got different information under PL microscopy and optical microscopy. The smallest dot size was ≈1.8 µm (Figure S40c, Supporting Information), which allows for high resolution patterning to increase the data capacity. We also demonstrated dynamic holographic display (Figure S41, Supporting Information) using ultrasmall CsPbBr_3_ QDs excited by 405‐nm holographic light.

### 2.4 Heat, Light Irradiation, and Lasing Stability of NWs

**Figure 5:**
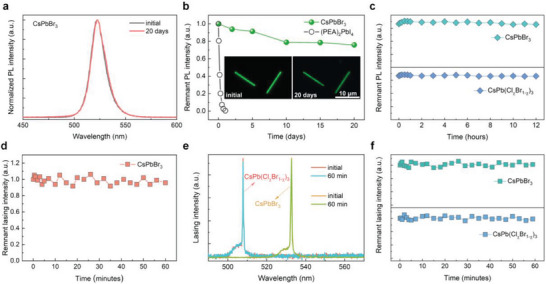
Stability of MHPs and lasing. a) PL spectra of CsPbBr_3_ NWs before and after storage for 20 days under 85 °C. b) PL intensity of CsPbBr_3_ NWs and 2D (C_8_H_9_NH_3_)_2_PbI_4_ (denoted as (PEA)_2_PbI_4_) as a function of time under 85 °C. Inset: PL image of CsPbBr_3_ NWs. c) PL intensity of CsPbBr_3_ and CsPb(Cl*_x_* Br_1‐_ *_x_*)_3_ NWs (*R*_Cl/Br_ = 1.5/1.5) as a function of irradiation time of 405 nm continuous laser with power density of 0.4 W cm^−2^. d) Lasing intensity of CsPbBr_3_ NWs as a function of time under 405 nm femtosecond laser irradiation (≈10^8^ pulses). e) Lasing spectra of CsPbBr_3_ and CsPb(Cl*_x_* Br_1‐_ *_x_*)_3_ NWs before and after irradiation for 1 h. f) Lasing intensity of CsPbBr_3_ and CsPb(Cl*_x_* Br_1‐_ *_x_*)_3_ NWs as a function of time under 1030 nm femtosecond laser irradiation.

The stability of MHPs was tested. The CsPbBr_3_ NWs exhibited impressive stability (**Figure** **5**a), and PL retained ≈80% after 20 days under 85 °C ambient conditions (Figure 5b). CsPb(Cl*_x_* Br_1−_ *_x_*)_3_ NWs remained stable after exposure to 405‐nm continuous laser for 12 h without an obvious change in the PL spectrum (Figure S42, Supporting Information) and intensity (Figure 5c), suggesting no degradation or phase segregation in the mixed‐halide perovskites. Figure 5d shows that the NW lasers can be stable under 405‐nm femtosecond laser irradiation for 1 h. The MPP lasing peak did not shift (Figure 5e) and the lasing intensity (Figure 5f) did not decrease after 1 h irradiation with a 1030 nm femtosecond laser. The lasing stability further evidences the high stability of MHP NWs. The high stability of MHP anisotropic structures may originate from the high crystal quality. As a reference, heating at 85 °C and 405 nm light irradiation could induce severe degradation in MHPs and phase segregation in mixed‐halide perovskites.^[^ ^9^, ^16^, ^65^ ^]^ The stability of NWs was verified by preparing 2D layered (C_8_H_9_NH_3_)_2_PbI_4_ perovskites with emission at 527 nm (Figure S43a, Supporting Information) that were reported to show superior stability to 3D MHPs because of the suppression of ion migration and water‐induced decomposition by the hydrophobic organic amine cations in the crystal lattice (Figure S43b, Supporting Information).^[^ ^66^ ^]^ In addition, with CsPbBr_3_ NWs under higher temperature conditions like 150 °C for 1 day, nearly half of the PL intensity was also remained (Figure S44, Supporting Information). The CsPbBr_3_ NWs showed better stability than (C_8_H_9_NH_3_)_2_PbI_4_ flakes (Figure 4b) and organic–inorganic NWs.^[^ ^67^ ^]^ Wrapping a layer of polymer or 2D material like graphene would promise in further enhancing the stability of perovskite NWs in the future.

## 3 Conclusion

In summary, high relative humidity‐directed fabrication of perovskite nanowires on glass surfaces under ambient conditions (25 °C and 85% relative humidity) was developed based on humidity‐directed precursor glass corrosion with Cs^+^, Pb^2+^, halide elements exportation. Density functional theory calculations revealed that H_2_O molecule adsorption could contribute to a significantly increased difference in facet energies between the (100) and (011) facets of CsPbBr_3_ nanowires, and thus facilitated 1D growth of CsPbBr_3_ along the [100] direction predominantly. The water‐MHP interaction is crucial to ensure high‐quality MHPs with smooth surfaces and enables single‐mode photonic nanolasers in MHP NWs with their heights at sub‐100‐nm scale. These MHP nanolasers possess high quality factor, high DOP, and high stability, and will find broad applications in nonlinear optics, subwavelength photonics, and integrated photonic devices at sub‐100‐nm scale. It also demonstrated versatility to prepare nanowire and nanoplate of halide stoichiometry‐dependent perovskites, which also exhibited excellent optical performance like high DOP and highly‐polarized single‐mode lasing. Additionally, by manipulating the relative humidity, tetrahedrons were prepared with varied compositions. The current work is a new room‐temperature vapor‐phase and on‐substrate strategy to grow MHP NWs, which would shape the future for preparing anisotropic nanostructures and expanding optics and optoelectronic applications.

## Conflict of Interest

The authors declare no conflict of interest.

## Author Contributions

Z.W. and X.L. contributed equally to this work. D.T. conceived the idea, organized, and supervised the project. Z.W. performed the experiments and collected the data. X.L. performed the theoretical calculation. C.C. constructed light path for relevant optical measurements. J.W. carried out the experiments for holographic display. D.T. interpreted the results and wrote the manuscript. K.G., Z.L., K.S., Z.L., Z.X., L.L. P.W., S.B., and J.Q. contributed to the data analysis and discussion of the manuscript.

## Supporting information



Supporting Information

## Data Availability

The data that support the findings of this study are available from the corresponding author upon reasonable request.
