# Supplementary material for: High Relative Humidity‐Induced Growth of Perovskite Nanowires from Glass toward Single‐Mode Photonic Nanolasers at Sub‐100‐nm Scale
Source: Adv Sci (Weinh). 2024 Dec 12;12(5):2412397. doi: 10.1002/advs.202412397 (PMC11791984; doi:10.1002/advs.202412397)
Supplement: Supplementary file 1 — Supporting Information [file ADVS-12-2412397-s001.docx]

*Supporting information*

**High Relative Humidity-Induced Growth of Perovskite Nanowires from Glass toward Single-Mode Photonic Nanolasers at Sub-100-nm Scale**

Zhiqiang Wang^1^†, Xinkuo Li^1,2^†, Chenduan Chen^1^, Minhan Lou^1^, Jiajia Wu^1^, Kai Gao^1^, Zengling Li^1^, Ke Sun^3^, Zhou Li^4^, Zhu Xiao^4^, Linhan Li^4^, Pan Wang^5^, Sai Bai^6^, Jianrong Qiu^5^ & Dezhi Tan^1,2^*

**Affiliations:**

^1^Zhejiang Lab, Hangzhou 311121, China

^2^School of Materials Science and Engineering, Zhejiang University, Hangzhou 310058, China

^3^China International Science & Technology Cooperation Base for Laser Processing Robotics, Wenzhou University, Wenzhou 325035, China

^4^School of Materials Science and Engineering, Central South University, Changsha, Hunan 410083, China

^5^College of Optical Science and Engineering, Zhejiang University, Hangzhou 310027, China

^6^Institute of Fundamental and Frontier Sciences, University of Electronic Science and Technology of China, Chengdu 611731, China

*Corresponding author. Email: wctdz@zju.edu.cn

†These authors contributed equally to this work.

Materials and Methods

**Materials**

The chemicals were all used as purchased from Aladdin without further purification. H_3_BO_3_ (≥99%), NaPO_3_ (≥99%), KPO_3_ (≥99%), Al_2_O_3_ (99.99%), ZnO (99.9%), Cs_2_CO_3_ (99.9%), PbCl_2_ (≥99%), PbBr_2_ (≥99%), NaCl (99.5%), NaBr (≥99%).

The perovskite precursor glass (perovskite element-containing glass) was prepared via a melt-quenching method using chemical powders according to molar ratio of 10P_2_O_5_-40B_2_O_3_-5Na_2_O-5K_2_O-10ZnO-10Al_2_O_3_-7Cs_2_O-3PbX_2_-5NaX (where X is Cl or Br) with fully mixed. After melting in a covered alumina crucible at 1150 ℃ for 15 min, the melts were poured onto a preheated stage and pressed to be a plate of glass with 3 mm thickness. Subsequently, the perovskite precursor glass samples were annealed at 300℃ for 3 h in a muffle furnace to release the internal stress.

The nominal ratios of Cl/Br were set as 1/2, 1.5/1.5 and 2/1 in the Cl^−^-Br^−^ codoped glass, respectively.

**Methods**

**Relative humidity control for the growth of MHPs on glass surfaces**

The well-prepared glass samples (after polishing) were placed in a home-made automatic tunable humidity controller for the growth of specific MHPs on glass surfaces. The relative humidity and treatment time were freely configurated.

**Glass surface patterning with laser processing and humidity treatment**

A home-built laser processing system (Pharos, Light Conversion) with the wavelength of 1030 nm was used for the processing. The pulse duration and repetition frequency utilized here was 1 ps and 100 kHz, respectively. The laser power irradiated on the glass surface from an 100X (NA=0.9) objective was 6.5 mW detected by the power meter. A computer-controlled translation stage that could move three dimensionally with the spatial resolution of 0.1 μm was used to carve the designed pattern. A charge-coupled device (CCD) camera coupled to the computer was used for the real-time observation of the fabrication process.

Before laser patterning on the glass surface, the polished glass surface was wrapped by a thin layer of PMMA through a spin-coating method. Specifically, droplets of 2wt% PMMA in ethyl lactate solution were spin coated on the glass surface via a two-step rotating process (600r/min for 10 s followed by 3000r/min for 20 s). This coating procedure repeated 2 times.

A “2024” dot pattern without PL on glass surface was initially created by pulsed laser (1 ps, 100 kHz, 65 nJ), the as-processed glass was subsequently placed in the humidity controller (55% RH for 6 days) for the growth of emissive MHPs along the dots of pattern. Then, the commentary part of “8888” apart from “2024” was fabricated by laser with the same parameters.

**Optical measurement**

The optical images and PL images were captured from Olympus BX53M microscopy irradiated by a white light and a 360 nm LED light source, respectively. Micro-region PL spectra were obtained from a home-built confocal fluorescence spectrophotometer excited by 405 nm continuous-wave laser. Charge-coupled device (CCD) was used for the collection of PL waveguiding image of nanowires. The heat treatments (*e.g.*, 85 ºC heating) for the stability test were carried out under atmospheric conditions and the measured RH was generally lower than 10%, but without special control.

Optically pumped lasing measurements was carried out with the same micro-PL setup with optical multi-channel gratings (150/600/1800 g/mm) on the perovskite nanostructures grown on the initial glass. The temperature of samples for pumped lasing measurements was decreased to -190℃ with the support of liquid nitrogen. The 1030 nm pulsed laser (95 fs, 22 kHz) as excitation light was focused onto the MHPs sample surface by using a 20X objective (NA=0.45).

**Scanning electron microscopy (SEM) and Energy-dispersive spectroscopy (EDS)**

The scanning electron microscopy (SEM) images were obtained on a ZEISS Gemini SEM 300 field-emission SEM operated at 3 kV. Energy-dispersive spectroscopy (EDS) was performed on single nanowire transferred onto a conductive tape operating at 10.0 kV.

**Transmission electron microscopy (TEM)**

The nanowire for TEM test was prepared by dry transfer of as-grown CsPbBr_3_ nanostructures onto a TEM grid. The TEM images and Lattice fringe images were acquired on a transmission electron microscope (JEOL, JEM-2100F) at an accelerating voltage of 200 kV.

**X-ray diffraction (XRD)**

The XRD patterns of samples were characterized by Bruker D8 Advance X-ray diffractometer with a Cu Kα.

**Raman spectroscopy**

Raman spectra were collected from RENISHAW Invia micro confocal Raman spectrometer excited by a continuous-wave laser of 532 nm wavelength.

**Atomic force microscope (AFM)**

The morphology of the synthesized MHPs on their raw glass was examined with AFM in tapping mode (Bruker Dimension Icon).

**PLQYs**

The photoluminescence quantum yields were obtained by using a photoluminescence quantum yield spectrometer (Hamamatsu, Quantaurus-QY Plus C13534-12).

**PL waveguiding loss calculation**

The tool “plot profile” of software “Image J” was used for the calculation of waveguide transmission loss of nanowires.

**Holographic display**

Blue-color holographic dynamic display was achieved through a spatial light modulator (SLM, Holoeye PLUTO-2.1 UV-099).

**Density-functional theory calculation**

The structural and electronic properties of the CsPbBr_3_ nanowires were calculated by using the first-principles density-functional theory (DFT) and the projector augmented wave (PAW) method implemented in the Vienna *Ab initio* simulation package (VASP).^[1,2]^ The generalized gradient approximation (GGA) of the exchange-correlation potential in the form of the Perdew-Burke-Ernzerhof (PBE) was used for the calculations throughout this work. Monkhorst - Pack k-point meshes of 4 × 4 × 1 were used for the (100) and (011) slabs and 3 × 3 × 3 were used for bulk CsPbX_3_ supercells (X = Cl, Br, I and mixed-halide). Besides, a 10 Å vacuum layer was taken and the surface three layers of atoms were fully relaxed to simulate (011) slab adsorbing a water molecule. The plane wave cutoff energy for the expansion of wave functions was set at 520 eV. The conjugate-gradient algorithm was used in all structure models to fully relax the internal forces until energy differences were less than 0.01 meV, stress tensors were less than 0.02 eV/Å.

The facet energy of CsPbBr_3_ was calculated according to^[3]^:

$$E_{surf}=\frac{(E_{slab}-E_{bulk})}{2A}$$

where$E_{surf}$is the facet energy of CsPbBr_3_ nanowire model; $E_{slab}$ is the Gibbs free energy of slab CsPbBr_3_ model, $E_{bulk}$ is the Gibbs free energy of bulk CsPbBr_3_ model and *A* is crystal facet area.

The facet energy with adsorbing a water molecule was calculated based on:

$$E_{surf}=\frac{(E_{slab-H_{2}O}-E_{bulk}-E_{H_{2}O})}{2A}$$

where $E_{slab-H_{2}O}$ is the facet energy of CsPbBr_3_ nanowire adsorbing a water molecule; $E_{H_{2}O}$ is the Gibbs free energy of the water molecule.

**
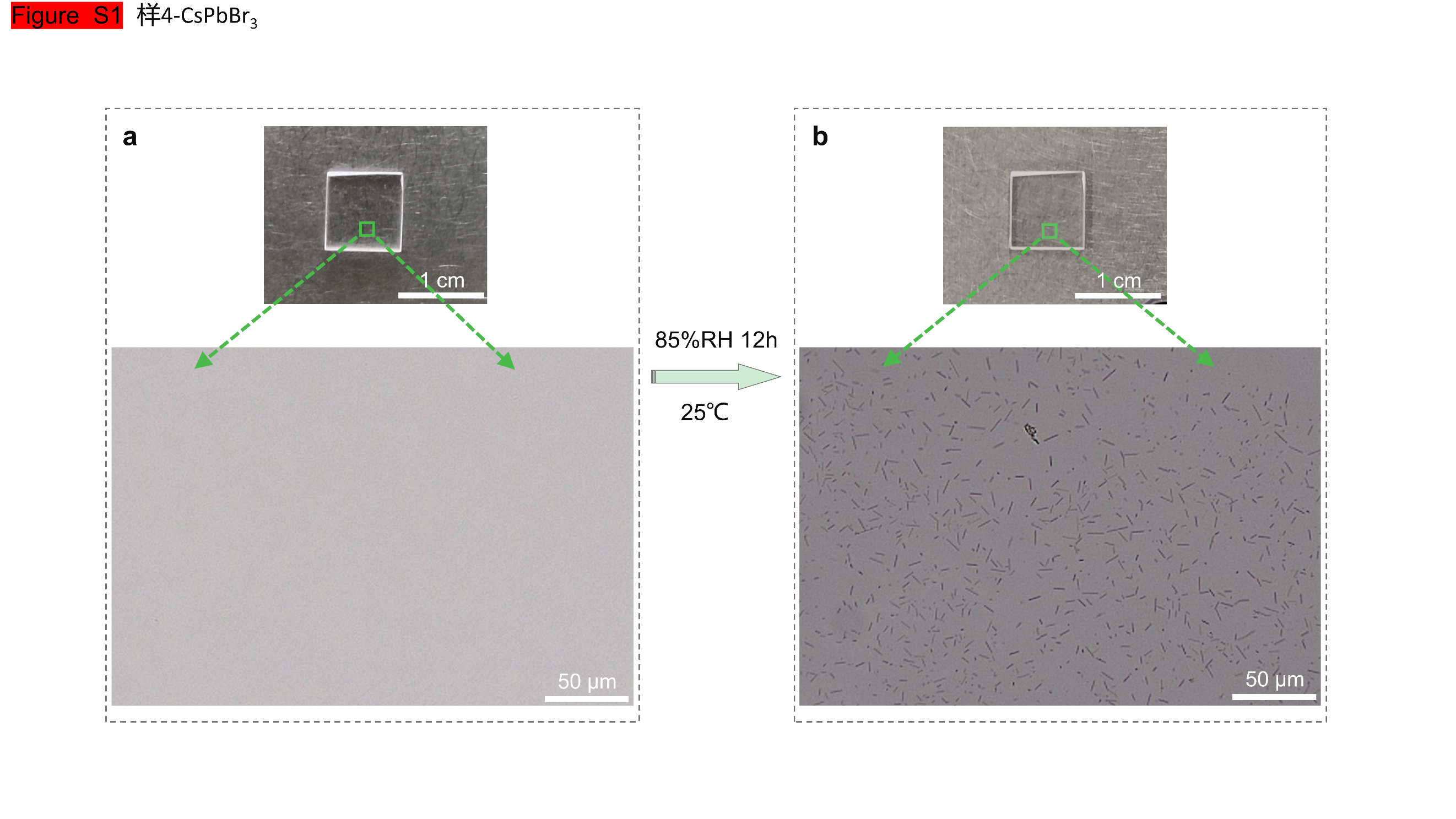
**

**Figure S1.** Optical images. (a) Glass before humidity treatment. (b) CsPbBr_3_ NWs grew on the glass surface after humidity treatment.


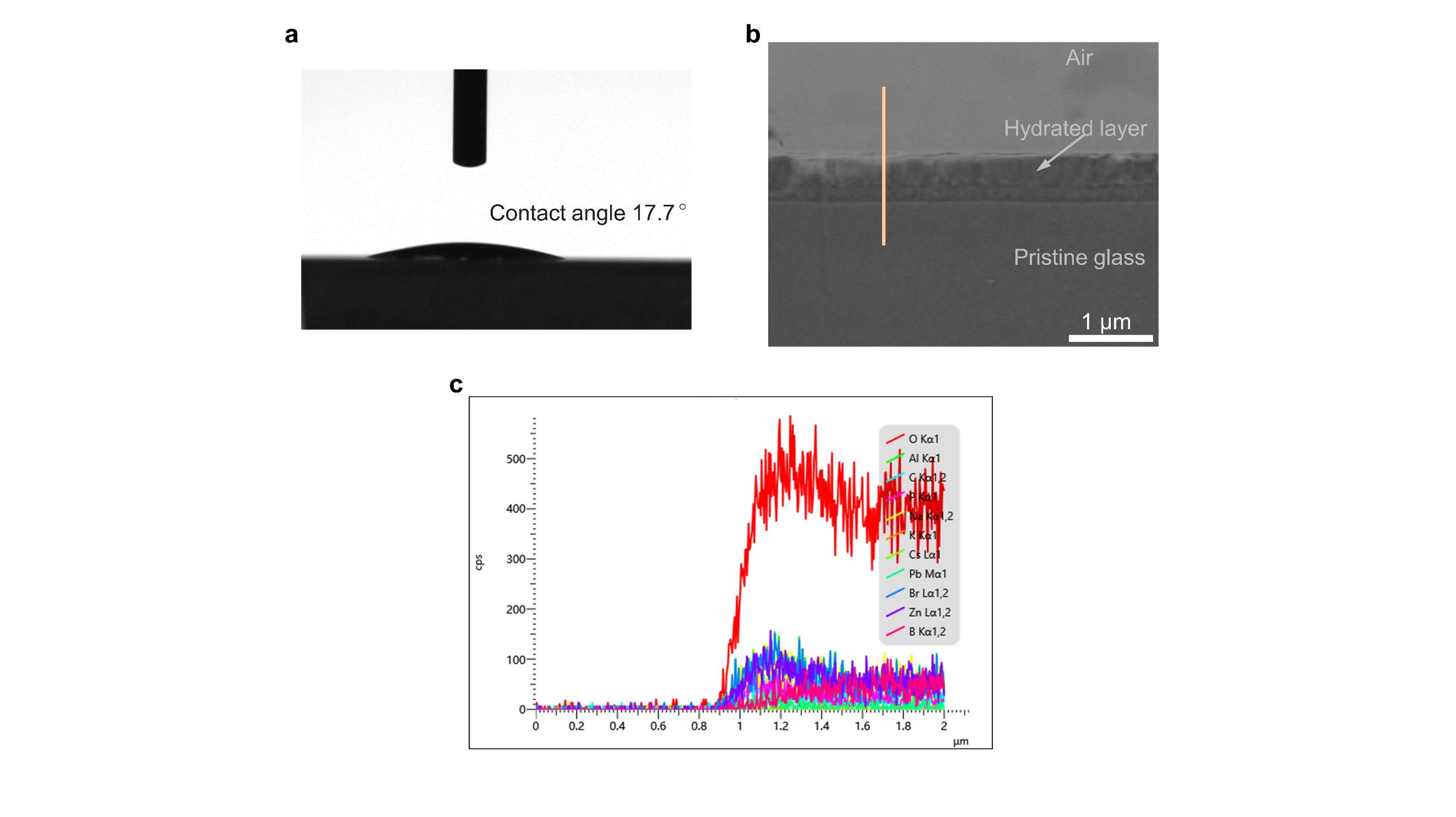


**Figure S2**. **Detection of the hydration on glass surface.** (a) The contact angle of water with glass (containing Cs^+^, Pb^2+^, Br^-^) surface. (b) A SEM image of the cross-sectional view of the hydrated layer after humidity treatment at 85%RH. (c) EDS line scan on the glass sample along the yellow line (top to bottom) in Figure S2b.

The contact angle of water with glass surface is 17.7° (Figure S2a), suggesting the highly hydrophilic nature of glass used in this work, and thus the water molecules can easily adsorb onto the glass surface at a relative high humidity of 85%. The hydrated layer was directly observed with a thickness less than 1 μm (Figure S2b), positioning on the surface of the glass. EDS line scan (Figure S2c) shows that the contents of ions are not homogeneous between the hydrated layer and the inner glass. It is noteworthy that the contents of multiple kinds of ions, for example, Na^+^, Al^3+^, Zn^2+^, Br^-^, are higher in the distance region of 1-1.4 μm (corresponding to the position of hydrated layer), verifying the diffusion of ions from the inside of glass to the glass surface. These results well demonstrated the formation of the hydrated layer and the ion diffusion process at the high relative humidity environment.


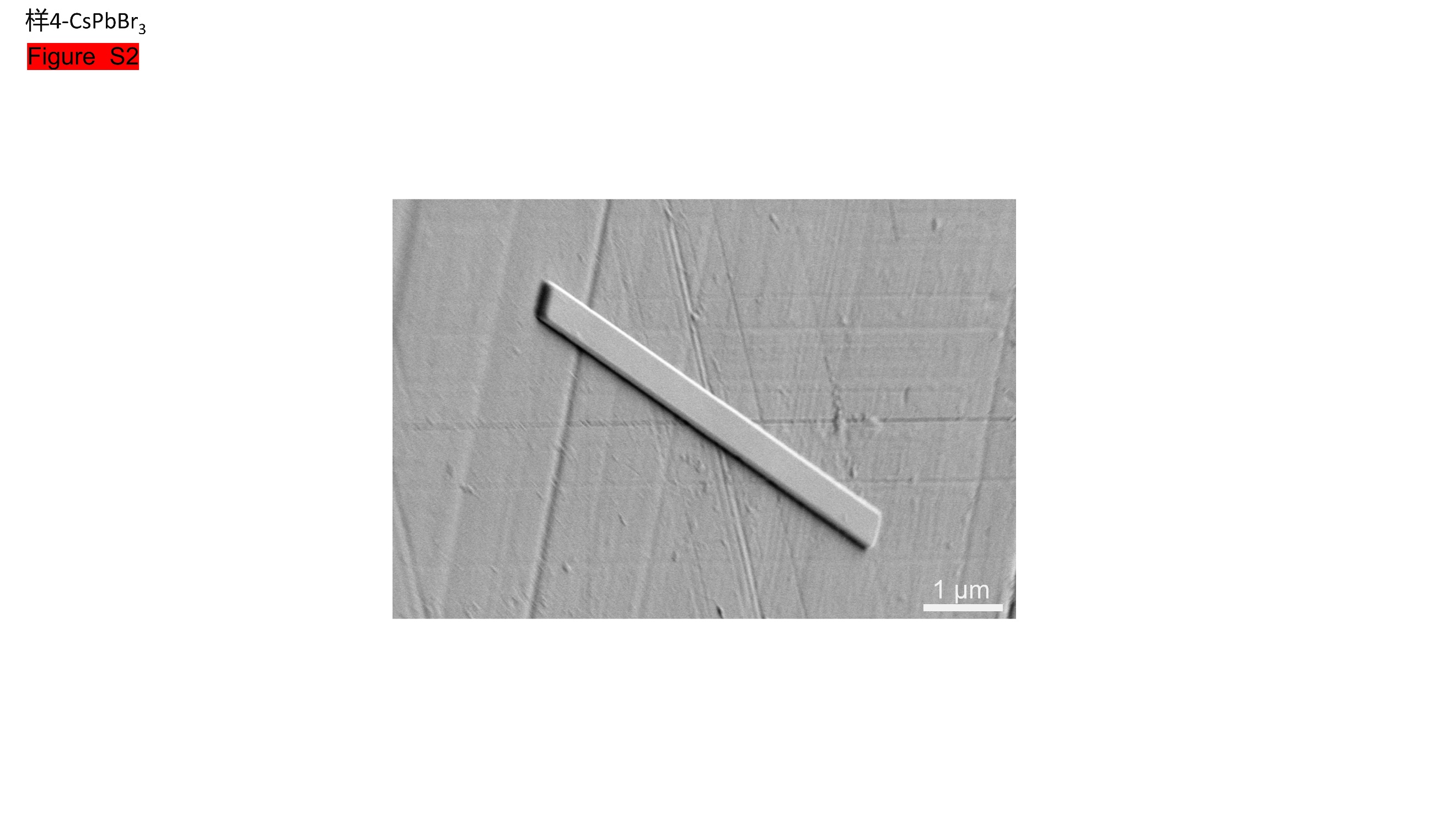


**Figure S3.** Tilt-view SEM image of a CsPbBr_3_ NW.


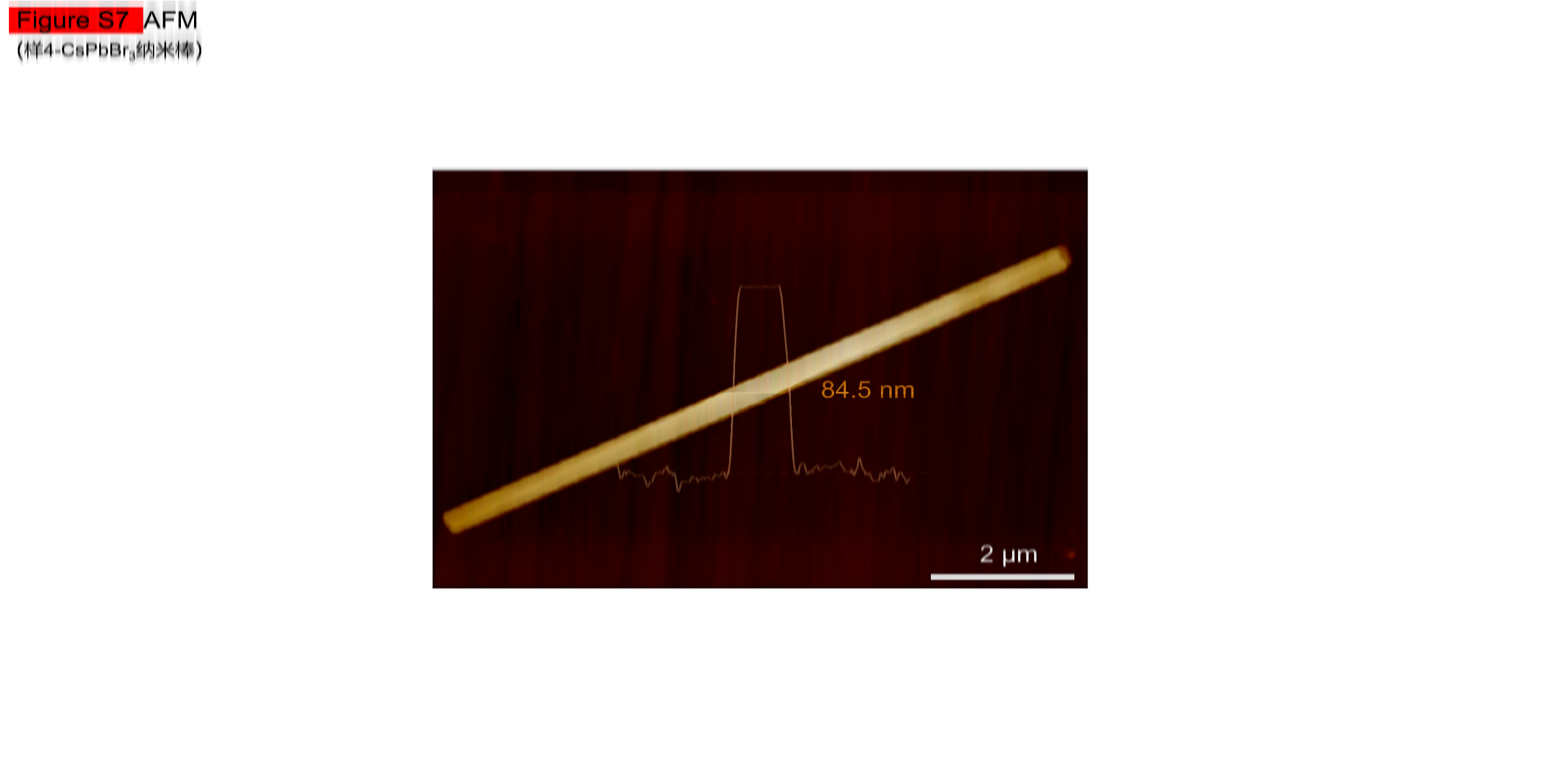


**Figure S4.** An AFM image and the height profile of a CsPbBr_3_ NW grew on the glass surface.


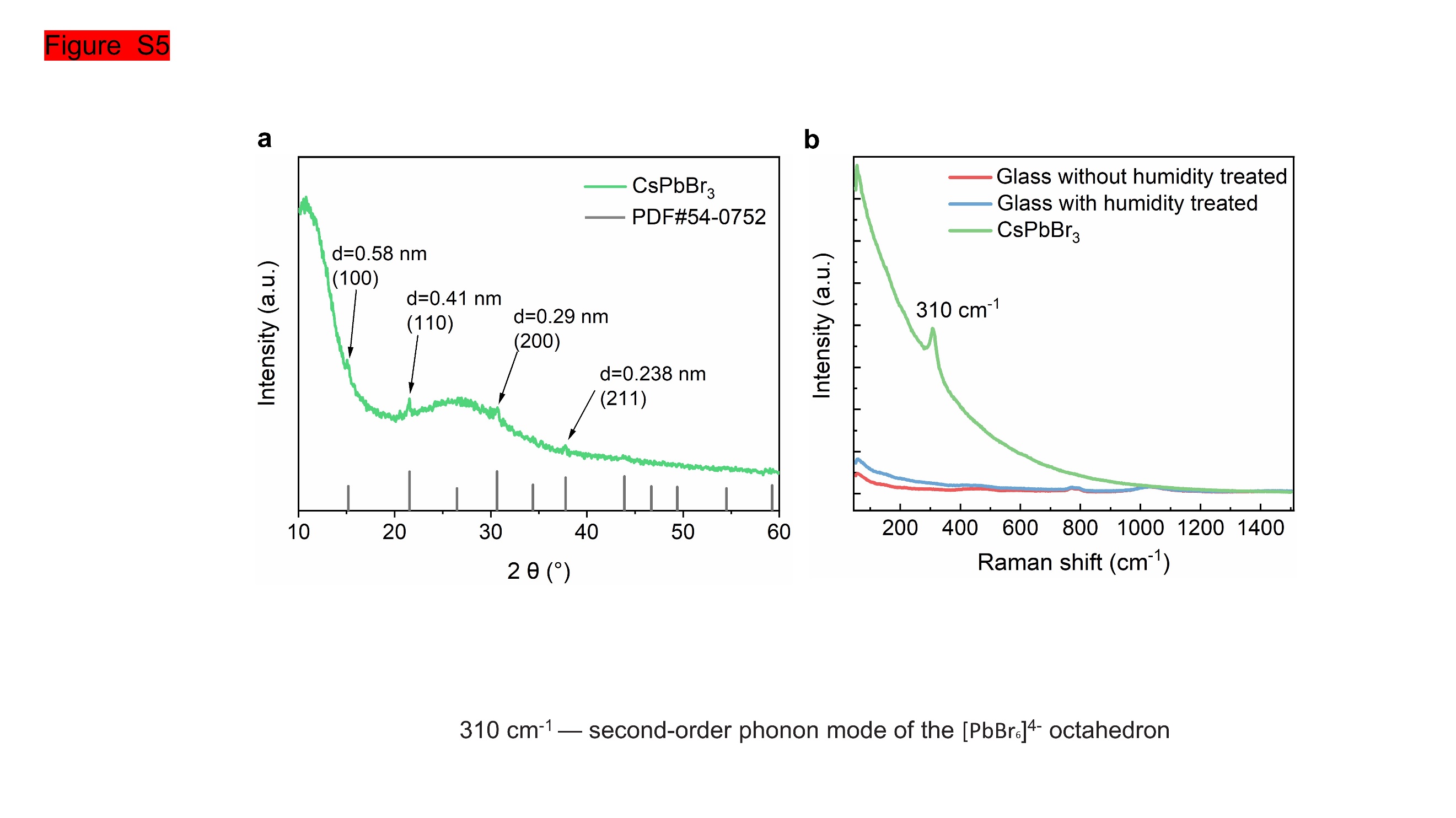


**Figure S5.** Structure characterizations. (a) XRD pattern of CsPbBr_3_ NWs. The diffraction reference is indexed to the cubic CsPbBr_3_ (PDF#54-0752). (b) Raman spectra of initial glass, humidity-treated glass and CsPbBr_3_ nanowires. 310 cm^-1^ is the characteristic peak of second-order phonon mode of the [PbBr_6_]^4-^ octahedron.


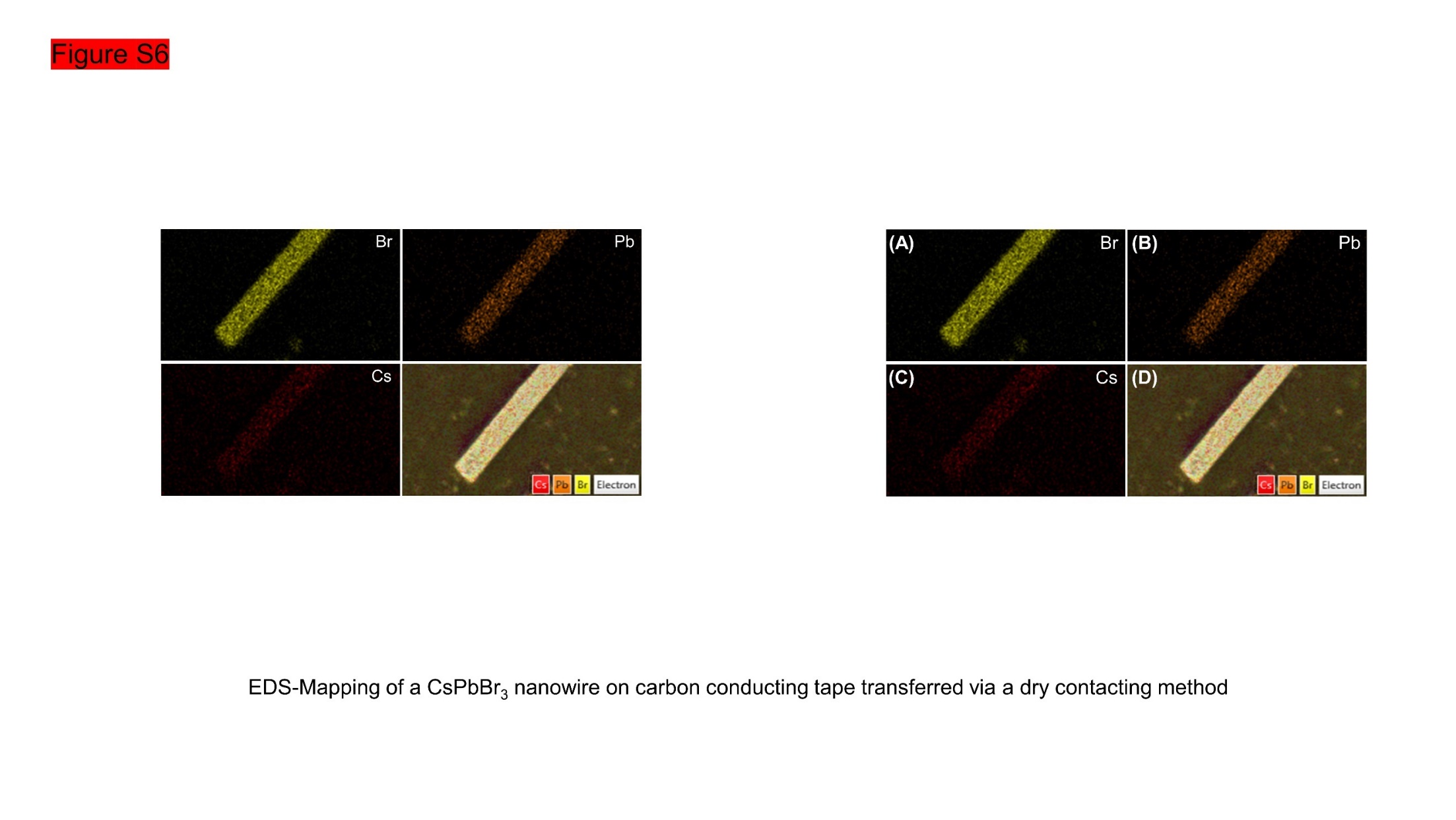


**Figure S6.** Element mappings of a CsPbBr_3_ NW with energy-dispersive spectroscopy.


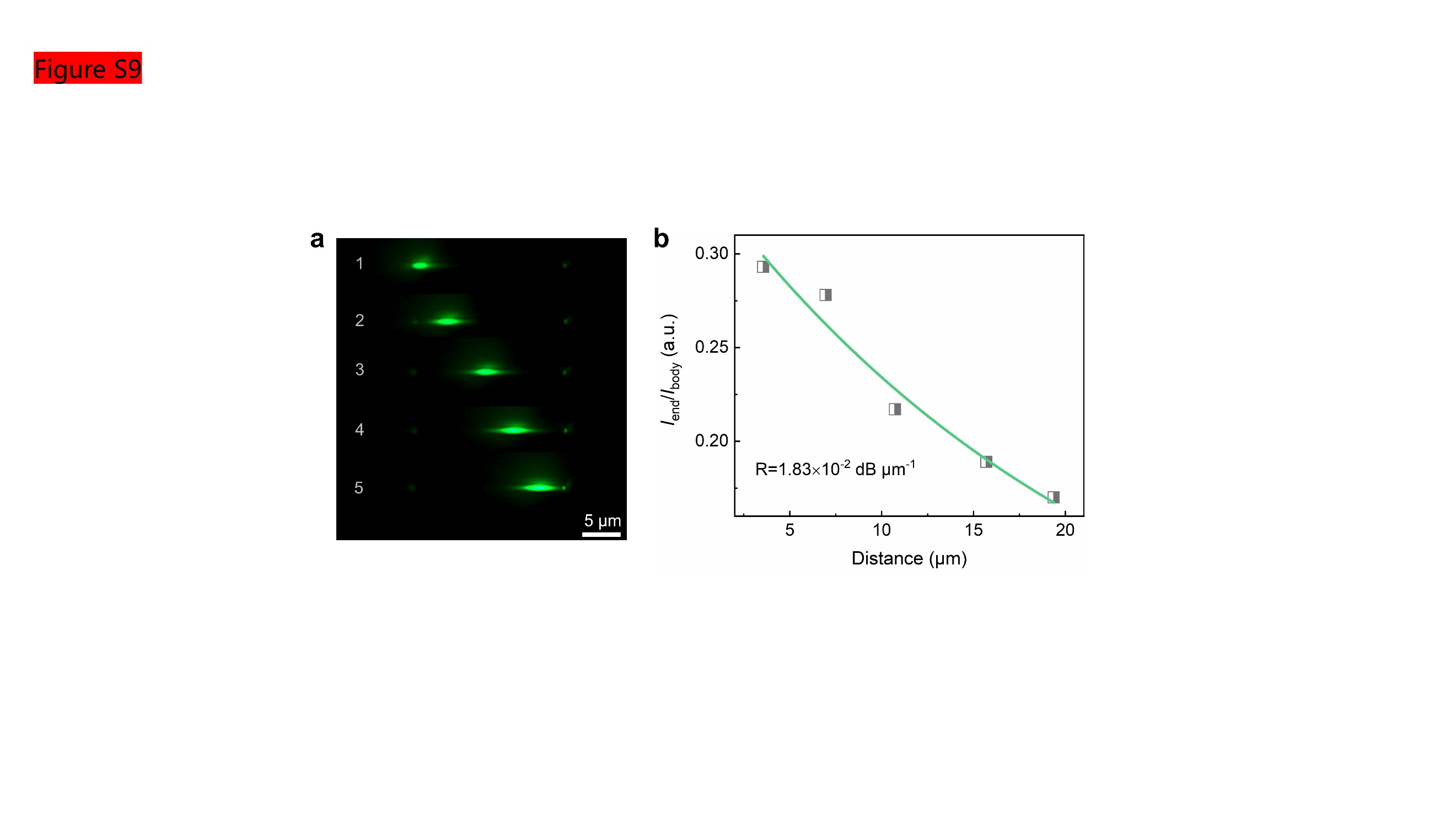


**Figure S7.** Waveguiding performance of CsPbBr_3_ NWs. (a) PL image of a NW excited with a 405 nm laser focused at different positions. (b) Ratio of the intensities at the end and focused positions of a CsPbBr_3_ NW (*I*_end_/*I*_body_). The fitting by curve was following the exponential decay function *I*_end_/*I*_body_ = kexp(–*RD*). *R* is the propagation loss and *D* is the propagating distance. k is a constant.


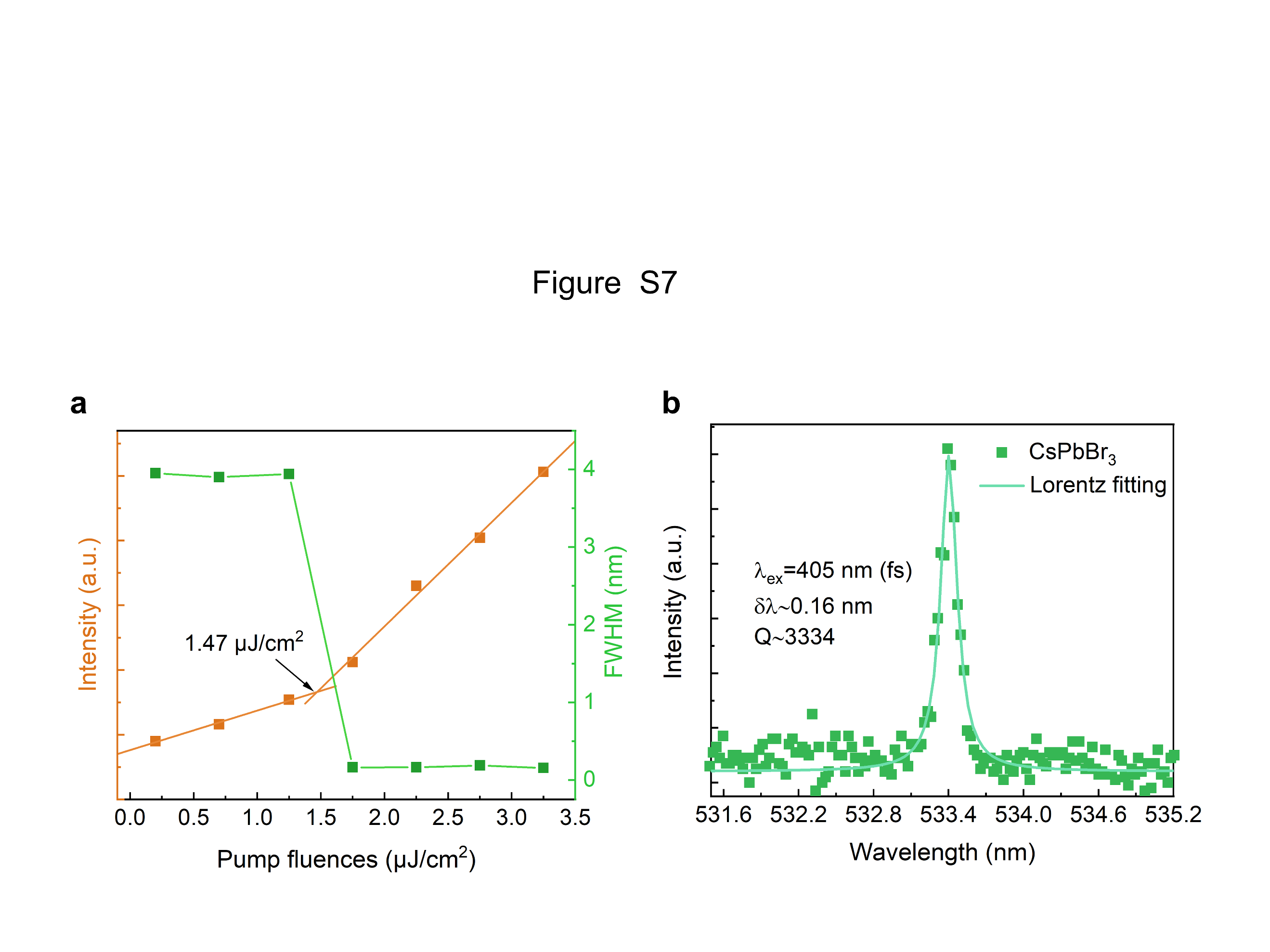


**Figure S8.** Lasing properties of a CsPbBr_3_ NW excited by a 405-nm femtosecond laser. (a) Lasing intensity and FWHM of as function of pumping fluence. (b) Lorentz fitting of the lasing spectrum.


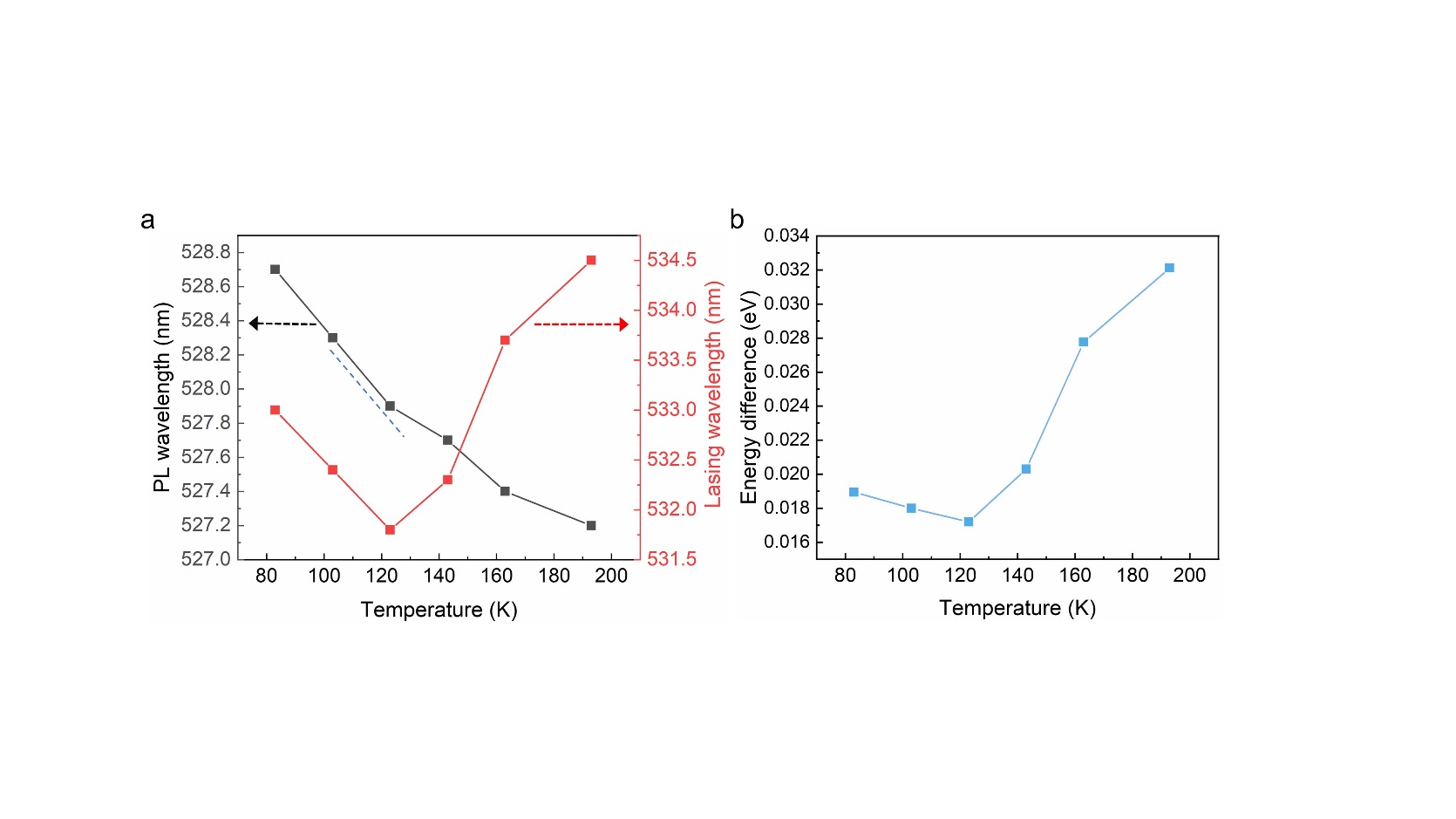


**Figure S9. Plot of the extracted data from Figure 1g**. (a) The extracted PL (black) and lasing (red) wavelengths as a function of temperature from 83 K to 193 K. (b) Plot of the evolution of energy differences between PL and lasing wavelengths with temperature increasing from 83 K to 193 K.

For a perovskite nanowire (NW), the peak center of PL emission is determined by the band gap. Increasing the temperature causes lattice expansion and correspondingly reduces the hybridization between the 4p orbital of Br and the 6s orbital of Pb (determine the value of valence state), and thus increases the band gap. On the other hand, electron-phonon interaction can broaden the valence state and conduction state, and thus cause the reduction of band gap. Hence, the dual function from lattice thermal expansion and electron-phonon interaction on perovskite NWs determines the value of band gaps at different temperatures. In our study, with the increase of temperature from 83 K to 123 K, a linear blue shift of PL emission was observed (Figure S9a), suggesting the major role of thermal expansion in tuning the band gap; however, in the range of 123 K to 193 K, the tendency of blue shift was slowed down with the increase of temperature, revealing that the electron-phonon interaction gradually becomes dominant. Based on these trends, we conjecture that the amplitude of increasement of electron-phonon interaction was larger than that of thermal expansion with temperature surpass 123 K. The analysis on the peak variation tendency of PL emission lays a solid foundation for the mechanism understanding of blue-shift and red-shift in lasing peaks.

The peak position of lasing is mainly determined by the range of gain spectrum relying on two factors, *i.e.*, the electronic band gap and the optical propagation loss. Lasing peaks usually locat at the smaller energy side of PL emission (similar to our work), being ascribed to the self-absorption effect due to the inelastic scattering with electronic-phonon interactions and defects. Via scattering, short-wavelength photons usually lose their energy and generate photons with a longer wavelength. With the increase of temperature from 83 K to 123 K, a blue shift of lasing peaks was observed (Figure S9a), in consistent with that of PL peaks, implying the key role of lattice thermal expansion effect and the increase of band gaps. While in the range of 123 K to 193 K, a red shift tendency of lasing peaks with the increase of temperature was observed and furthermore, the energy difference between PL peaks and lasing peaks becomes larger as the increase of temperature (Figure S9b). The red shift and the increased energy difference strongly reveal the rapidly increased optical propagation loss, where the self-absorption effect played a vital role in determining the lasing peaks. In general, the probability of defect scattering is less sensitive to the temperature. However, the electronic-phonon scattering increases with the temperature and thus increased the optical propagation loss. Moreover, the amplitude of electronic-phonon scattering becomes larger with temperature surpass 123 K, as was revealed from the variation tendency of PL peaks. Based on the red shift tendency of lasing, we concluded that electron-phonon interaction became dominant in the temperature range of 123 K to 193 K.


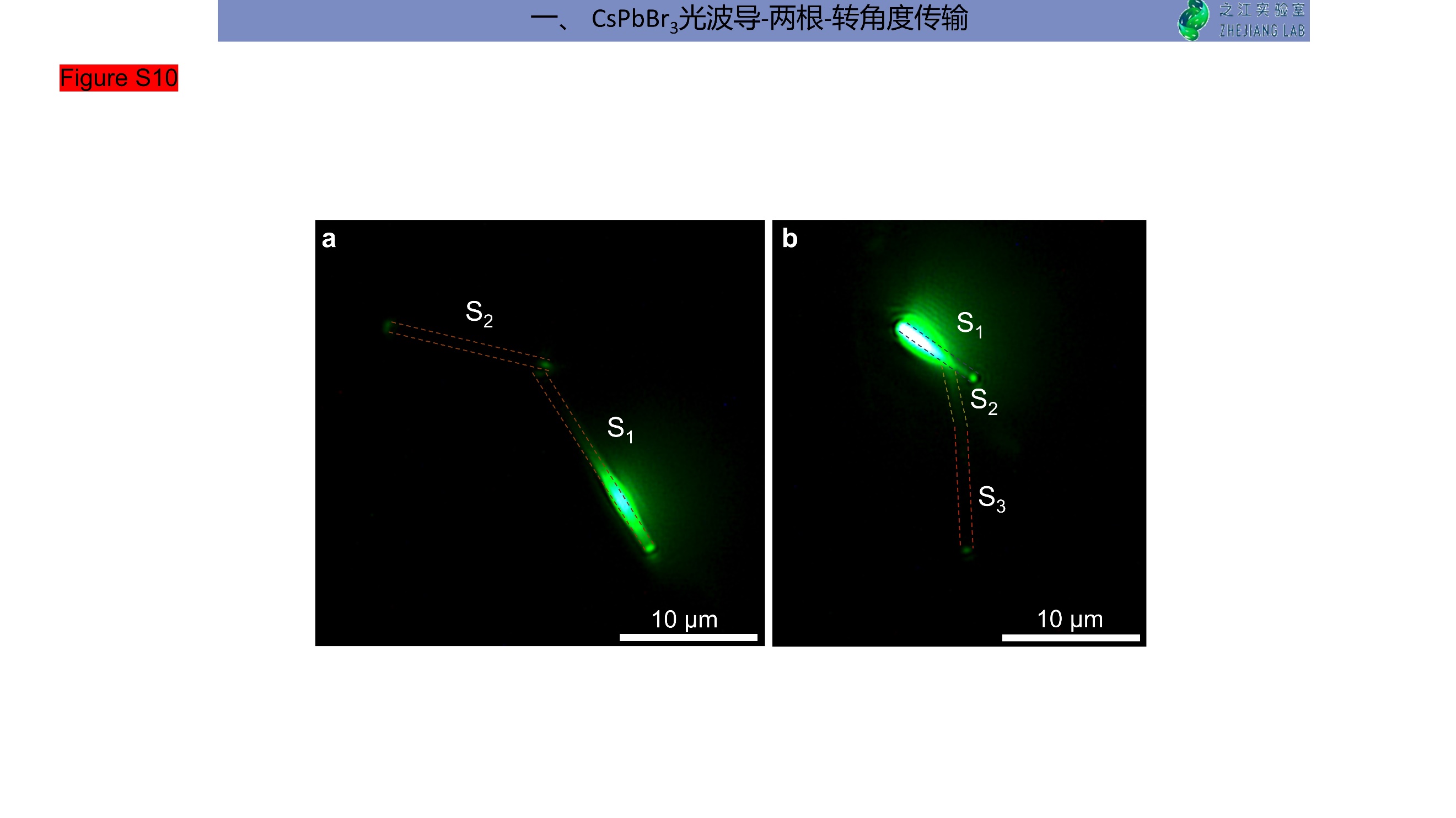


**Figure S10.** Waveguiding behavior of CsPbBr_3_ NWs. (a) The emission coupling to the neighboring nanowire and (b) to the third nanowire. The orange dashed rectangles indicate the waveguides.


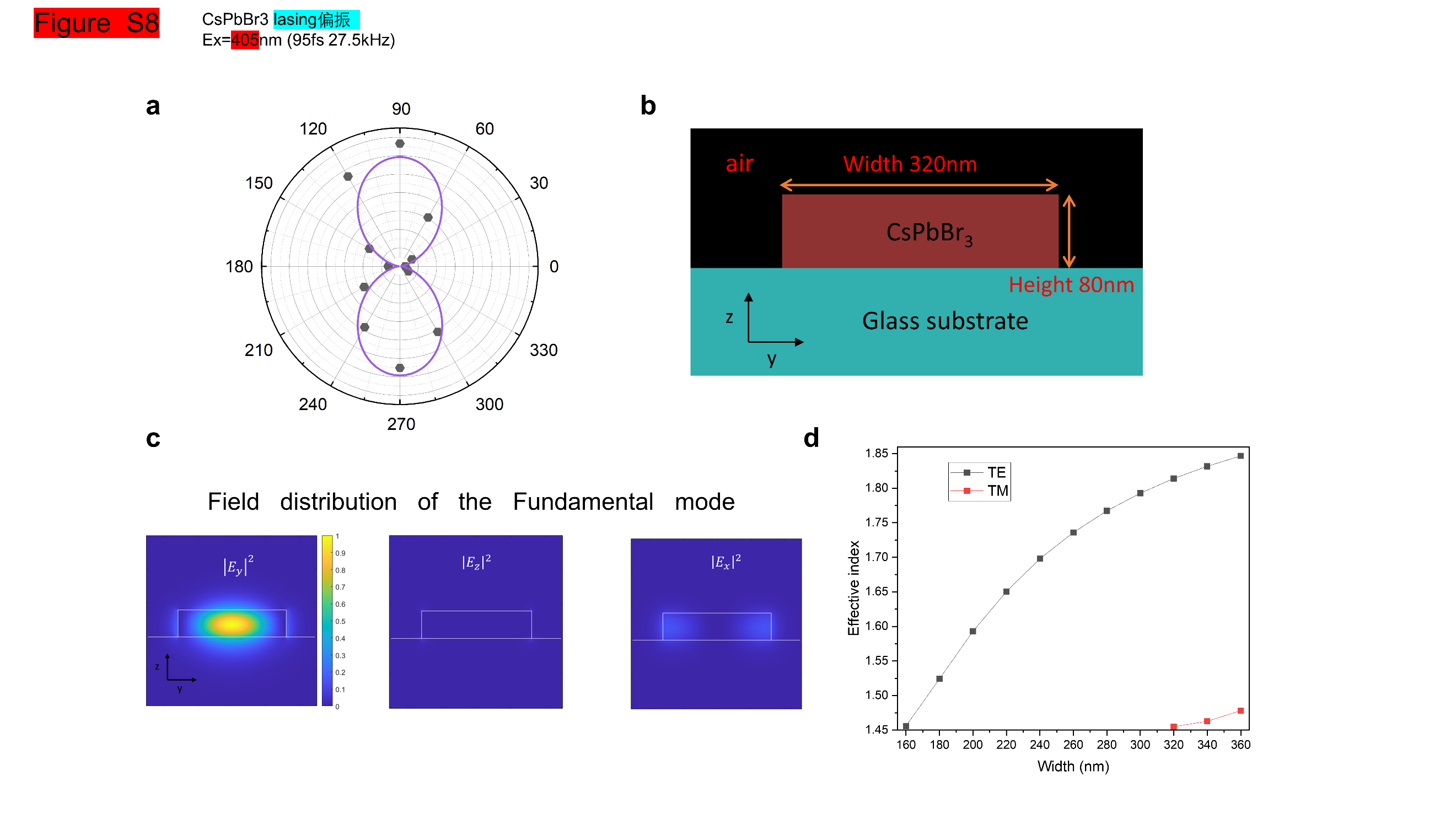


**Figure S11.** Polarization-dependent lasing and mode simulation. (a) Polarization-dependent lasing, 405-nm femtosecond laser as excitation source. (b) Schematic of a CsPbBr_3_ NW on the glass. (c) Cross-section of the simulated electric field intensity distribution at 532.8 nm for fundamental waveguide modes in a CsPbBr_3_ NW (width = 320 nm, height = 80 nm). $E_{y}$ component in the fundamental mode is dominant. Interestingly the TE-like mode has small portion of $E_{x}$ component and almost no $E_{z}$ component. Thus, the lasing mode supported by the fundamental waveguide mode is strongly polarized along y direction. (d) Simulation of the effective refractive index as a function of width of NWs. E_y_: along the substrate, perpendicular to the NW; E_z_: perpendicular to the substrate and NW; E_x_: along the NW. At the lasing wavelength of 532.8nm, the refractive index of glass and CsPbBr_3_ used in the simulation are 1.45 and 2.54, respectively. The fundamental waveguide mode is fundamental-TE-like mode. For simplicity, we call it TE mode. When the width of the nanowire is less than 320 nm, only TE mode can be supported. When the width is 320 nm, fundamental-TM-like mode (TM) appears.

**Figure S12.** Lasing properties of a CsPbBr_3_ NW excited by a 1030-nm femtosecond laser. (a) PL/lasing intensity and FWHM of the lasing spectra as a function of pump fluence. (b) Lasing intensity as a function of pump fluence. (c) Lasing spectrum and Lorentz fitting. (d) The ratio of side-mode suppression (R_SMS_) calculated following the formula of R_SMS_ = 10log_10_(*I*_d_/*I*_s_), where *I*_d_ and *I*_s_ are the intensity of the dominant mode and side mode, respectively.


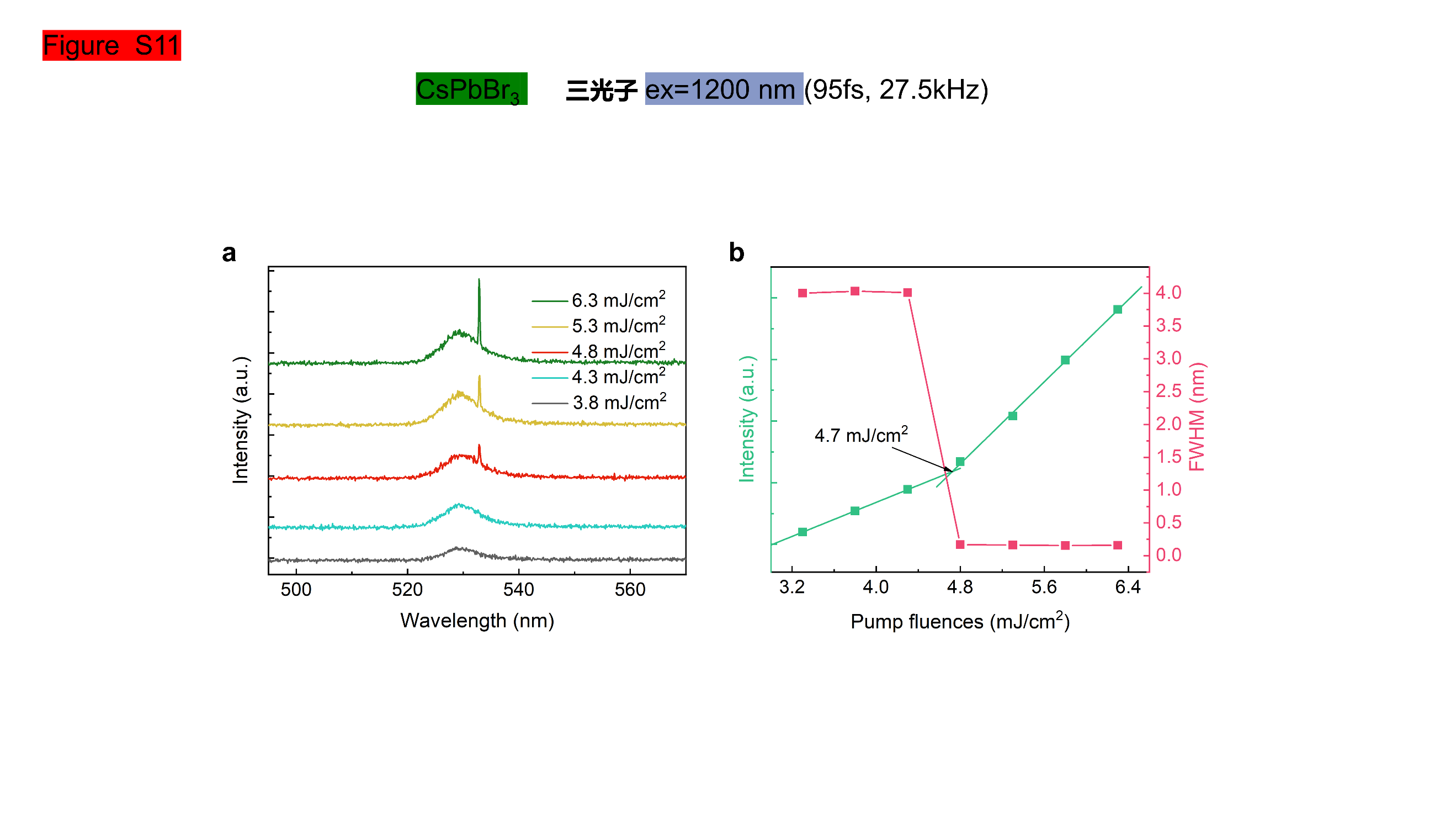


**Figure S13.** Three-photon pumped lasing properties of CsPbBr_3_ halide perovskite NW excited by a 1200-nm femtosecond laser. (a) Lasing spectra as a function of pump fluence. (b) Lasing intensity and FWHM as a function of pump fluence.


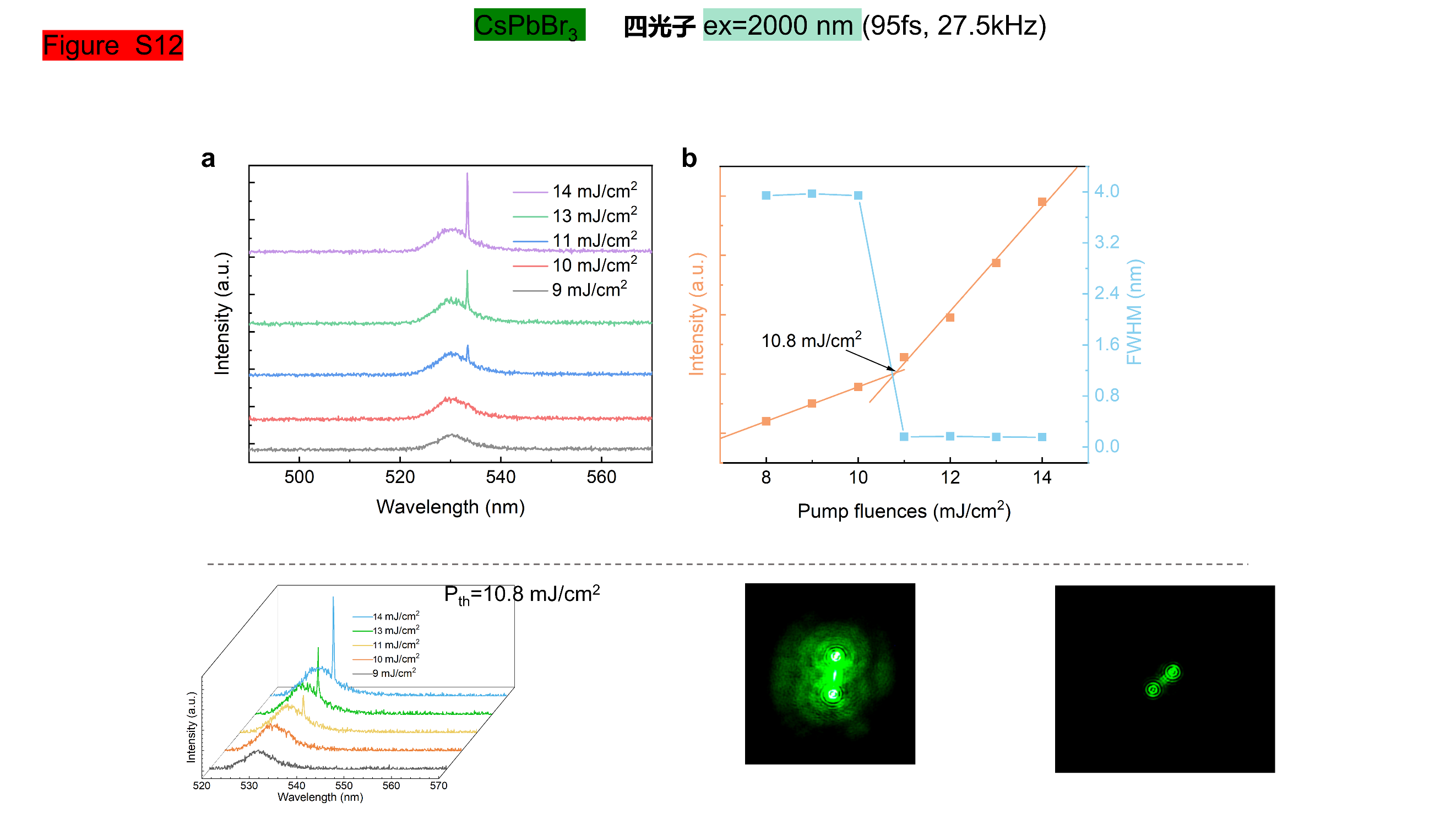


**Figure S14.** Four-photon pumped lasing properties of CsPbBr_3_ NWs excited by a 2000-nm femtosecond laser. (a) Lasing spectra as a function of pump fluence. (b) Lasing intensity and FWHM as a function of pump fluence.


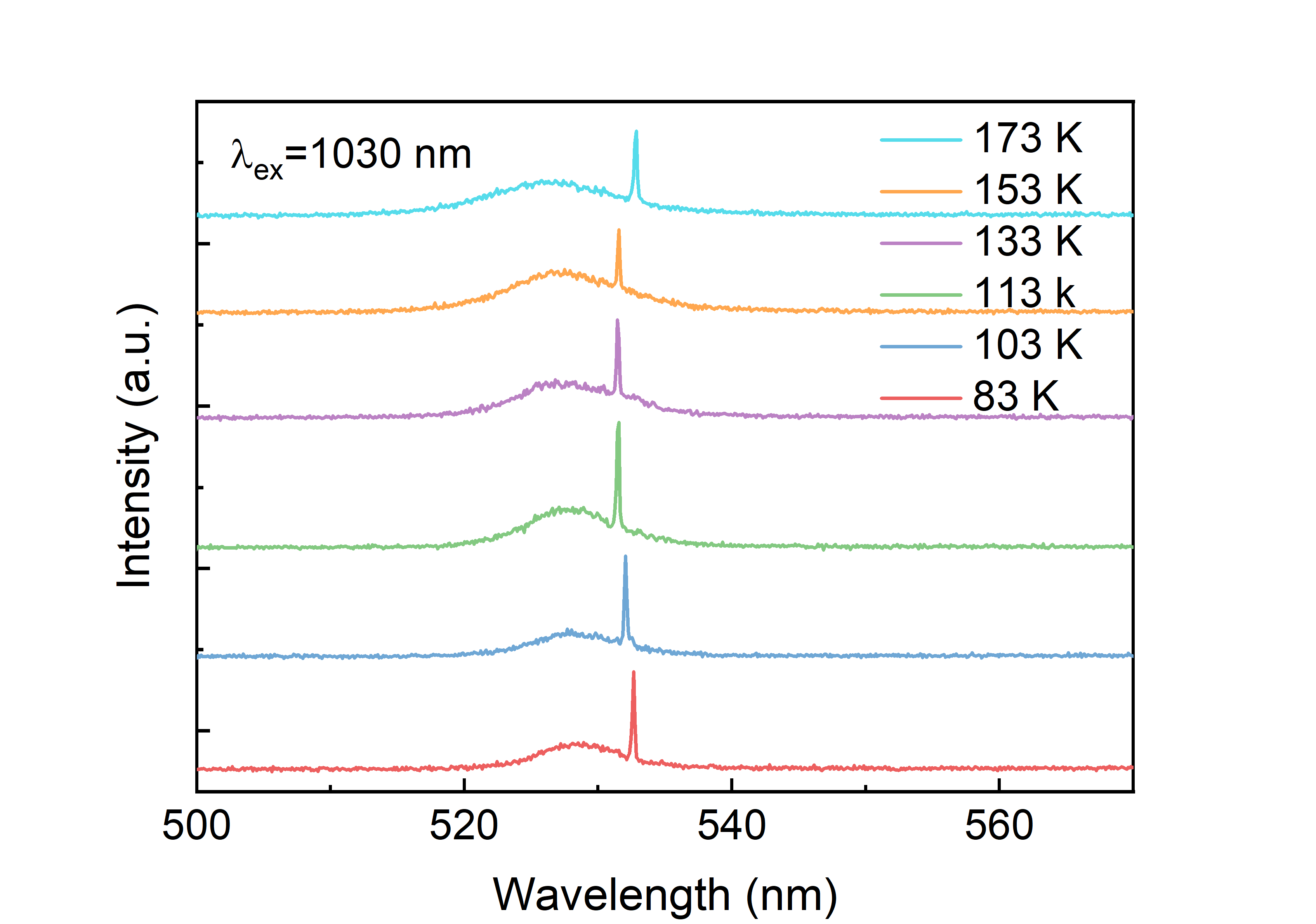


**Figure S15.** Lasing spectra of a CsPbBr_3_ NW excited by a 1030-nm femtosecond laser as a function of temperature.


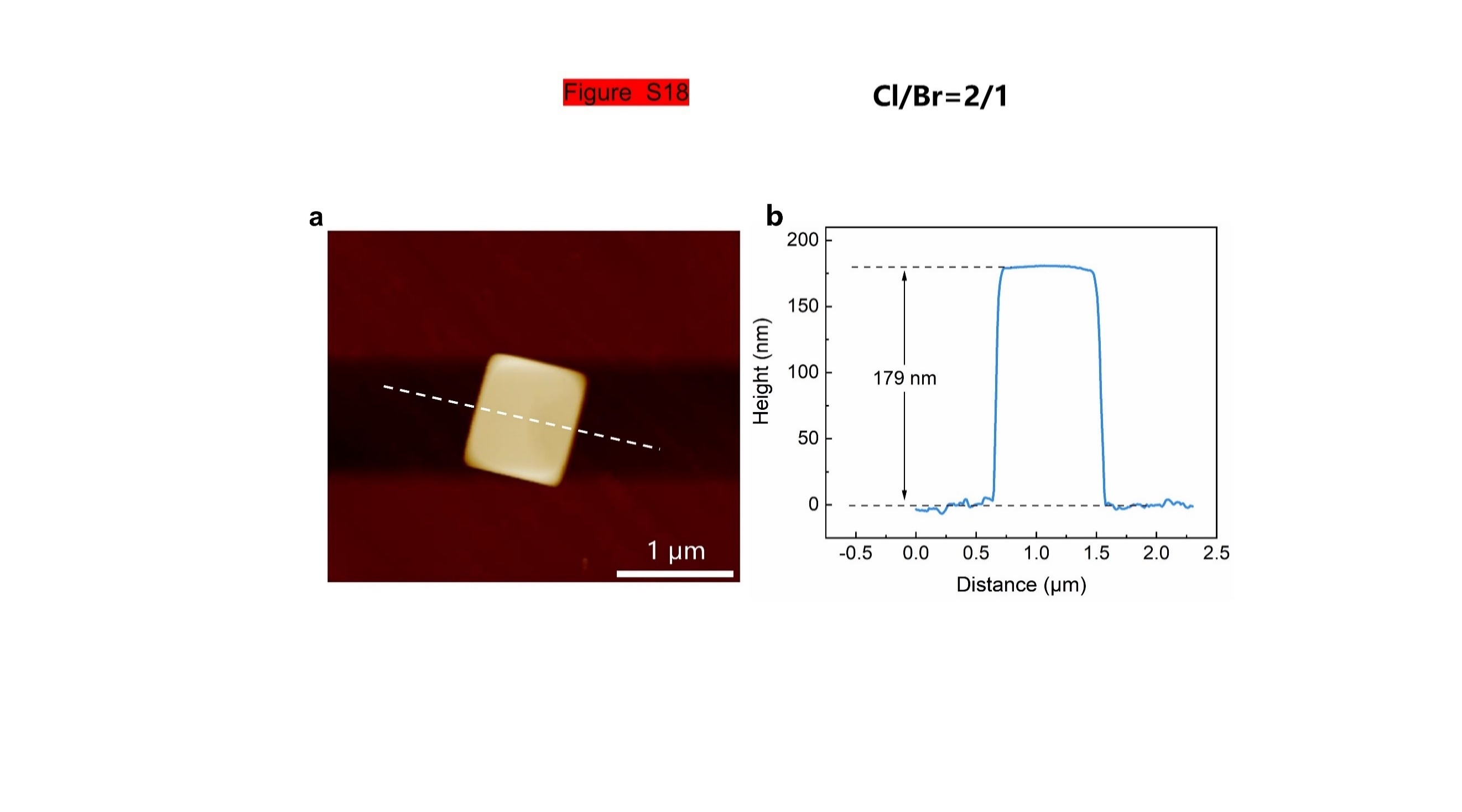


**Figure S16. AFM result of mixed-halide perovskites on glass.** (a) AFM image and (b) Height profile for *R*_Cl/Br_ = 2/1.

**Figure S17.** PL spectra for MHP structures on glasses with *R*_Cl/Br_ = 1/2, 1.5/1.5, and 2/1.


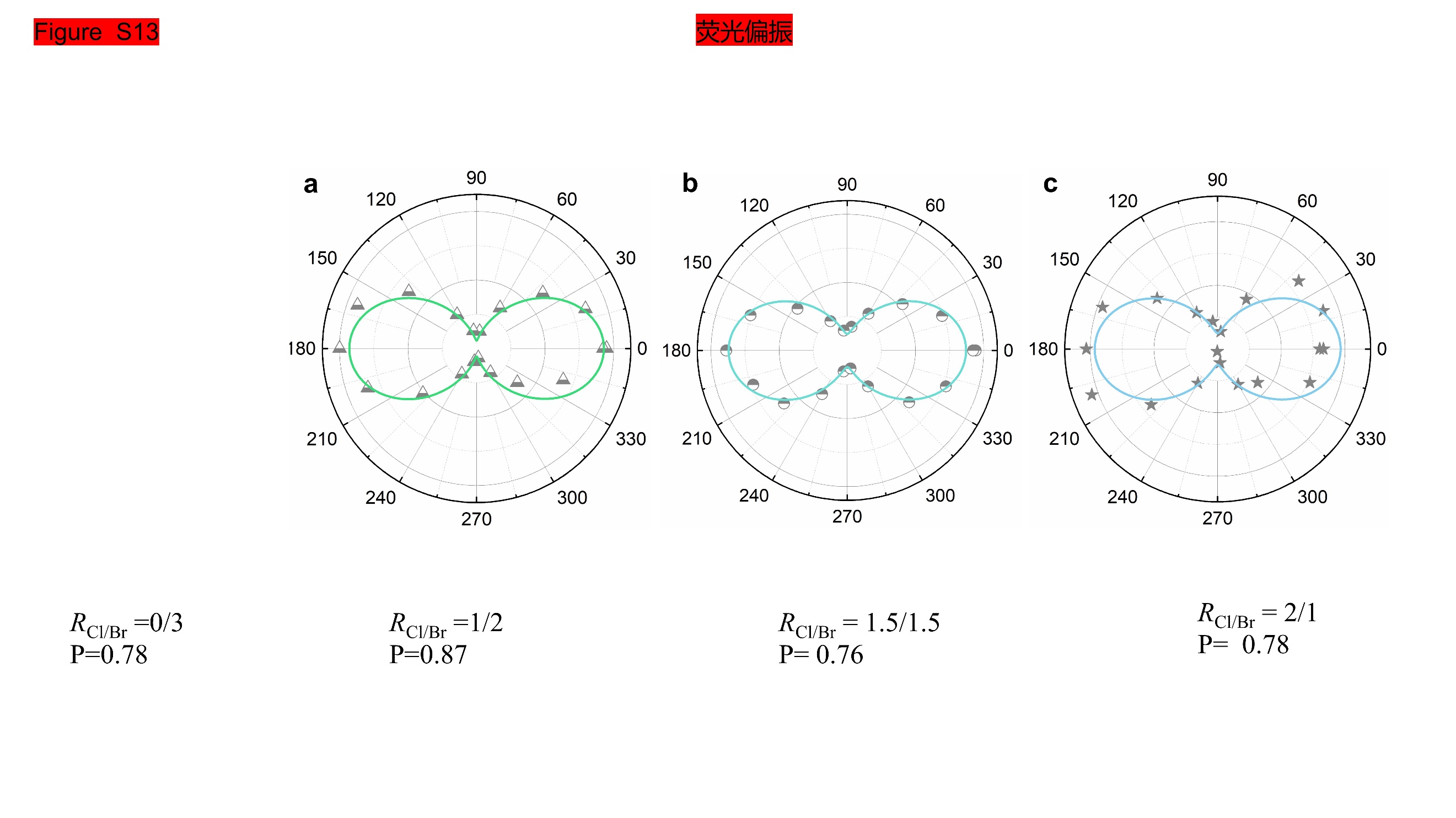


**Figure S18.** Polar plots of intensity of polarization dependent PL for MHPs synthesized on different glasses. (a) *R*_Cl/Br_ = 1/2. (b) *R*_Cl/Br_ = 1.5/1.5. (c) *R*_Cl/Br_ = 2/1.


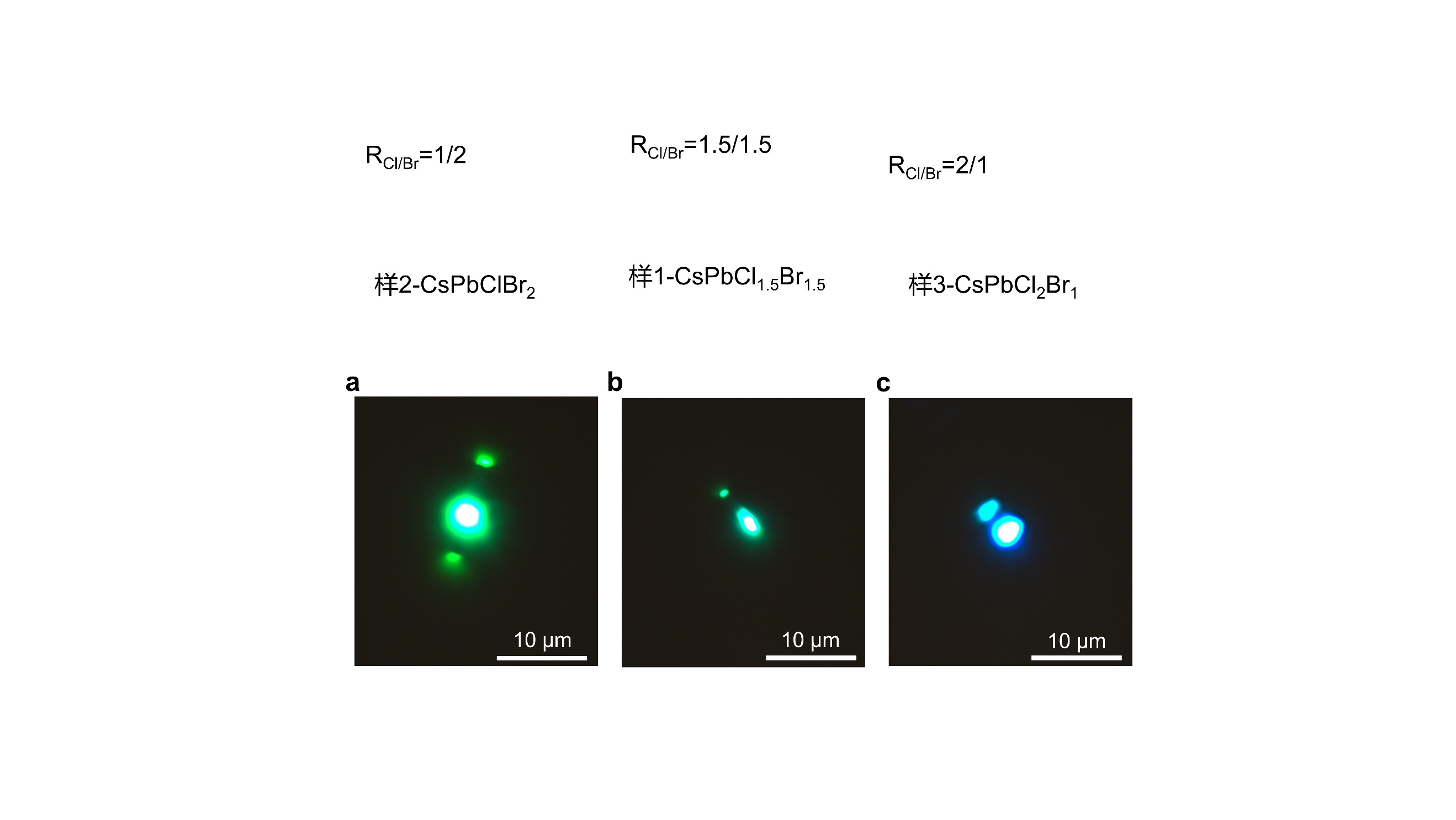


**Figure S19. PL Waveguiding behavior of mixed halide MHPs**. (a) *R*_Cl/Br_ = 1/2. (b) *R*_Cl/Br_ = 1.5/1.5. (c) *R*_Cl/Br_ = 2/1.


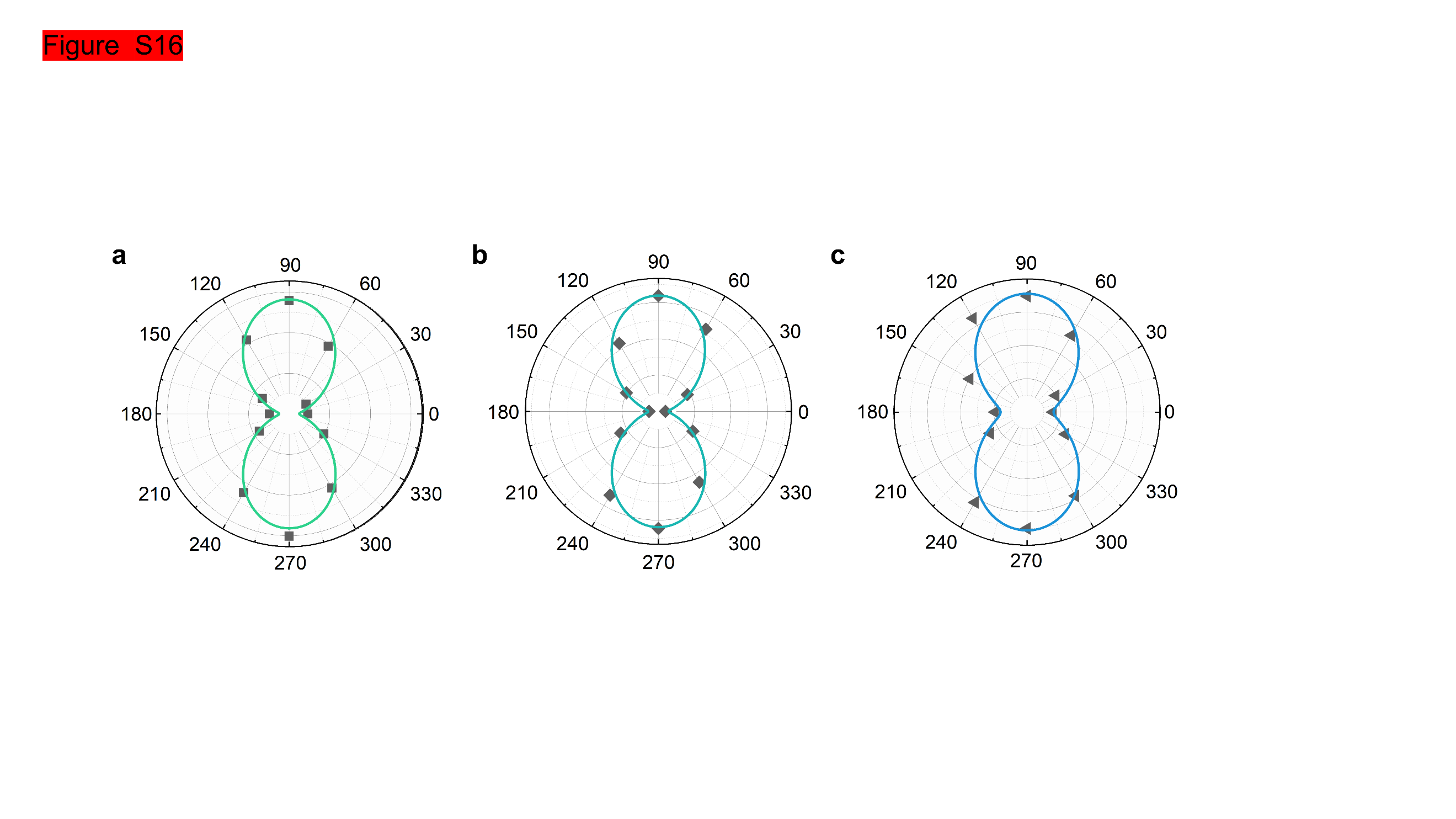


**Figure S20.** Polar plots of intensity of polarization dependent lasing for MHPs synthesized on different glasses. (a) *R*_Cl/Br_ = 1/2. (b) *R*_Cl/Br_ = 1.5/1.5. (c) *R*_Cl/Br_ = 2/1.


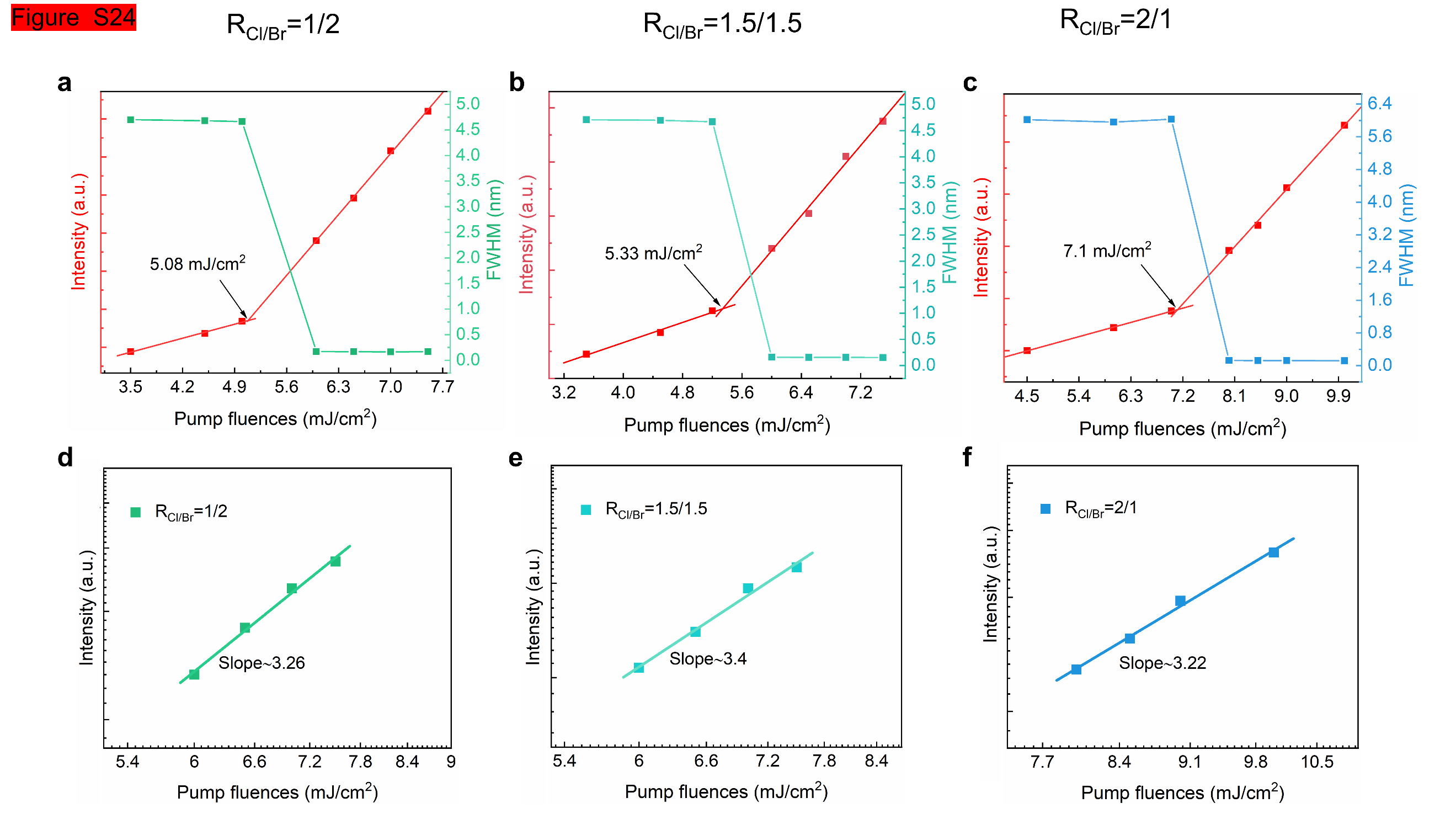


**Figure S21.** Lasing properties of MHPs synthesized on glasses with various *R*_Cl/Br_. (a, b, c, d, e, f) PL/lasing intensity and FWHM of the lasing spectra as a function of pump fluence. (a, d) *R*_Cl/Br_ = 1/2. (b, e) *R*_Cl/Br_ = 1.5/1.5. (c, f) *R*_Cl/Br_ = 2/1.


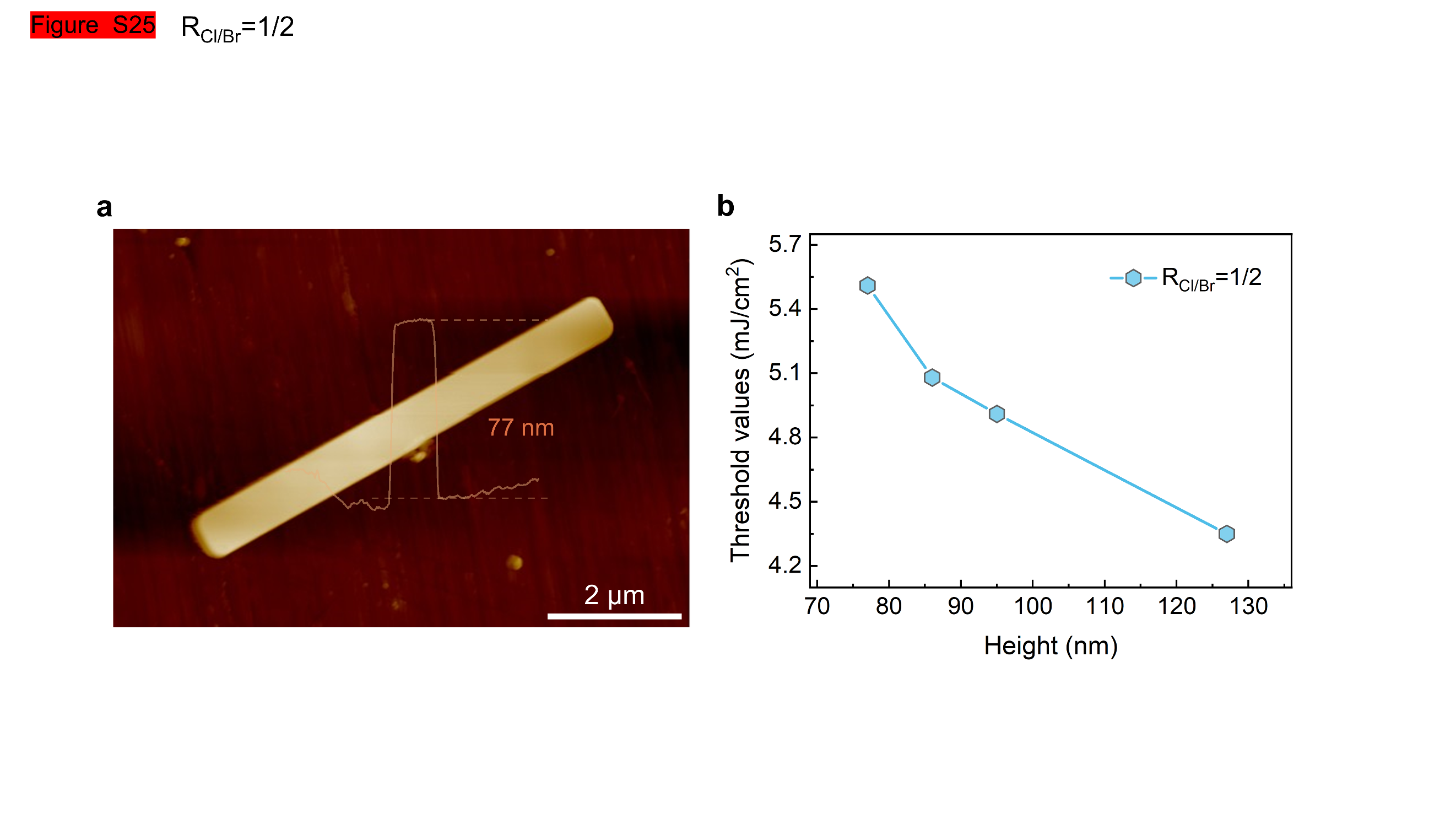


**Figure S22.** Lasing properties. (a) AFM result of a mixed-halide perovskite NW on the glass with *R*_Cl/Br_ = 1/2. (b) Pumping threshold as a function of the height.


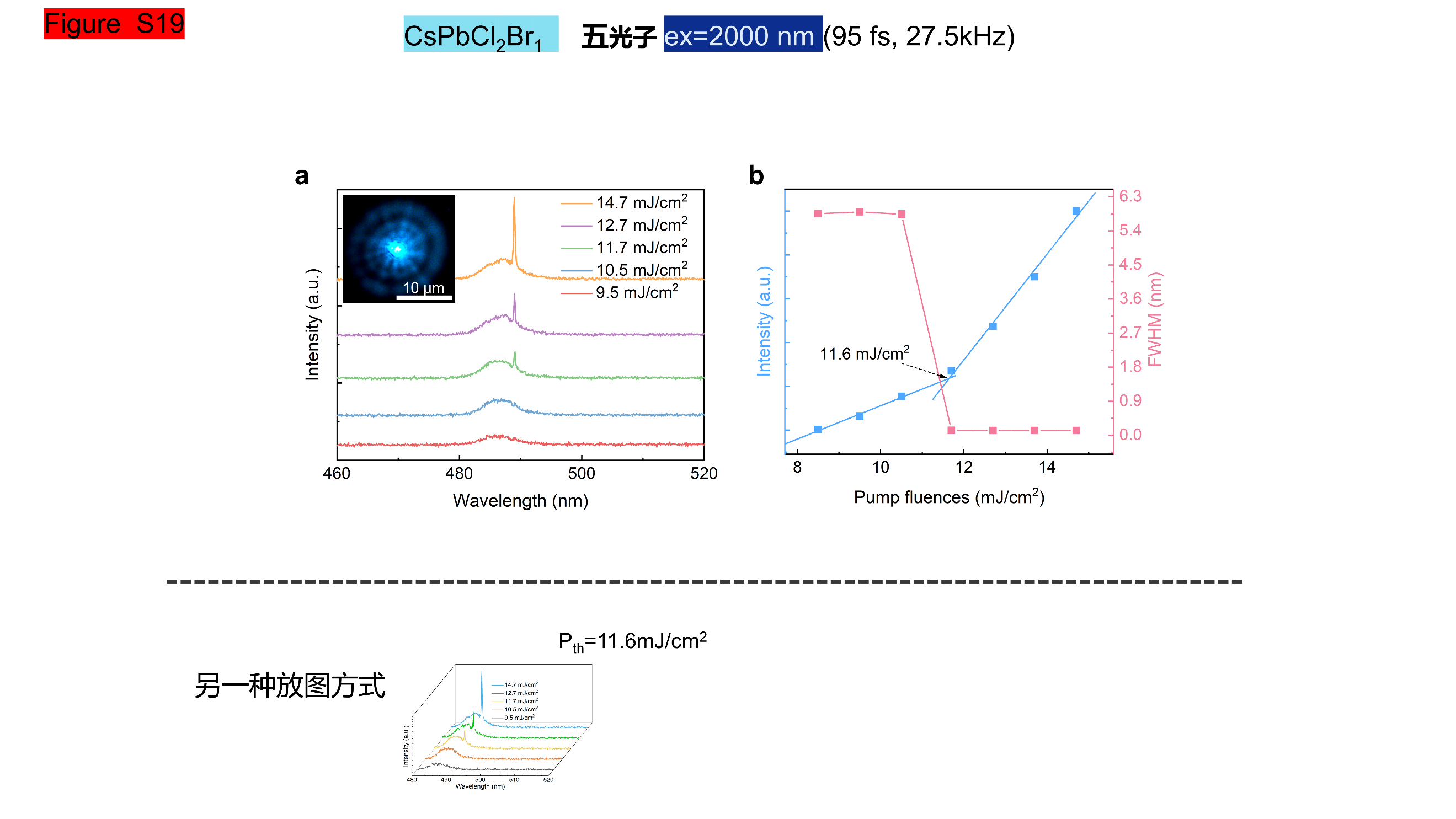


**Figure S23.** Five-photon pumped lasing properties of mixed halide perovskite microplatelets excited by a 2000-nm femtosecond laser. (a) Lasing image. (b) Lasing spectra as a function of pump fluence. (c) Lasing intensity and FWHM as a function of pump fluence.


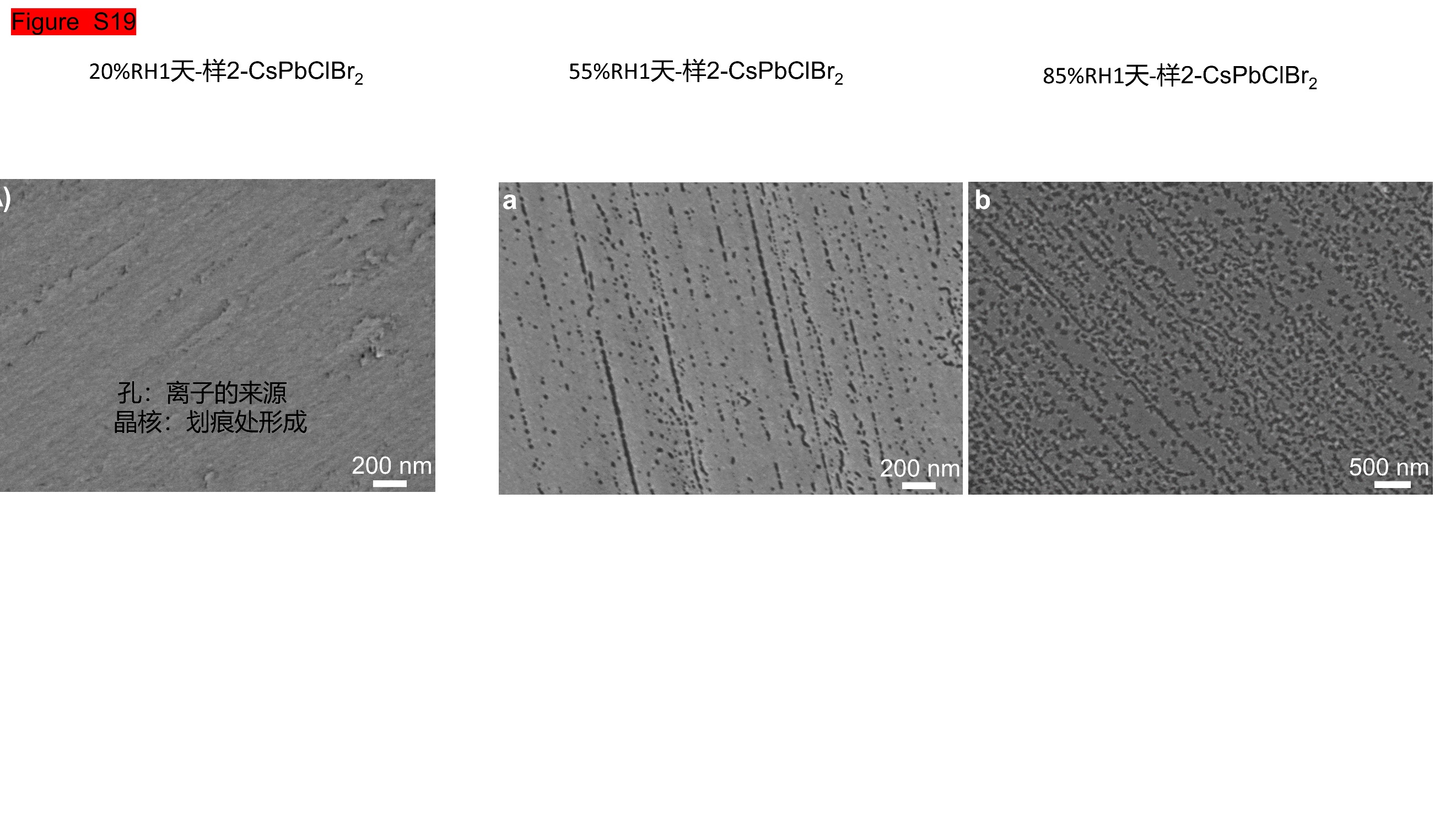


**Figure S24.** Glass surface after humidity induced structure modification for 1 day with various RH. (a) 55% RH. (b) 85% RH. More holes were easily observed along the scratching lines that were produced during glass polishing and this is reasonable that more defects may be present in these lines, accelerating glass corrosion and MHP nucleation.


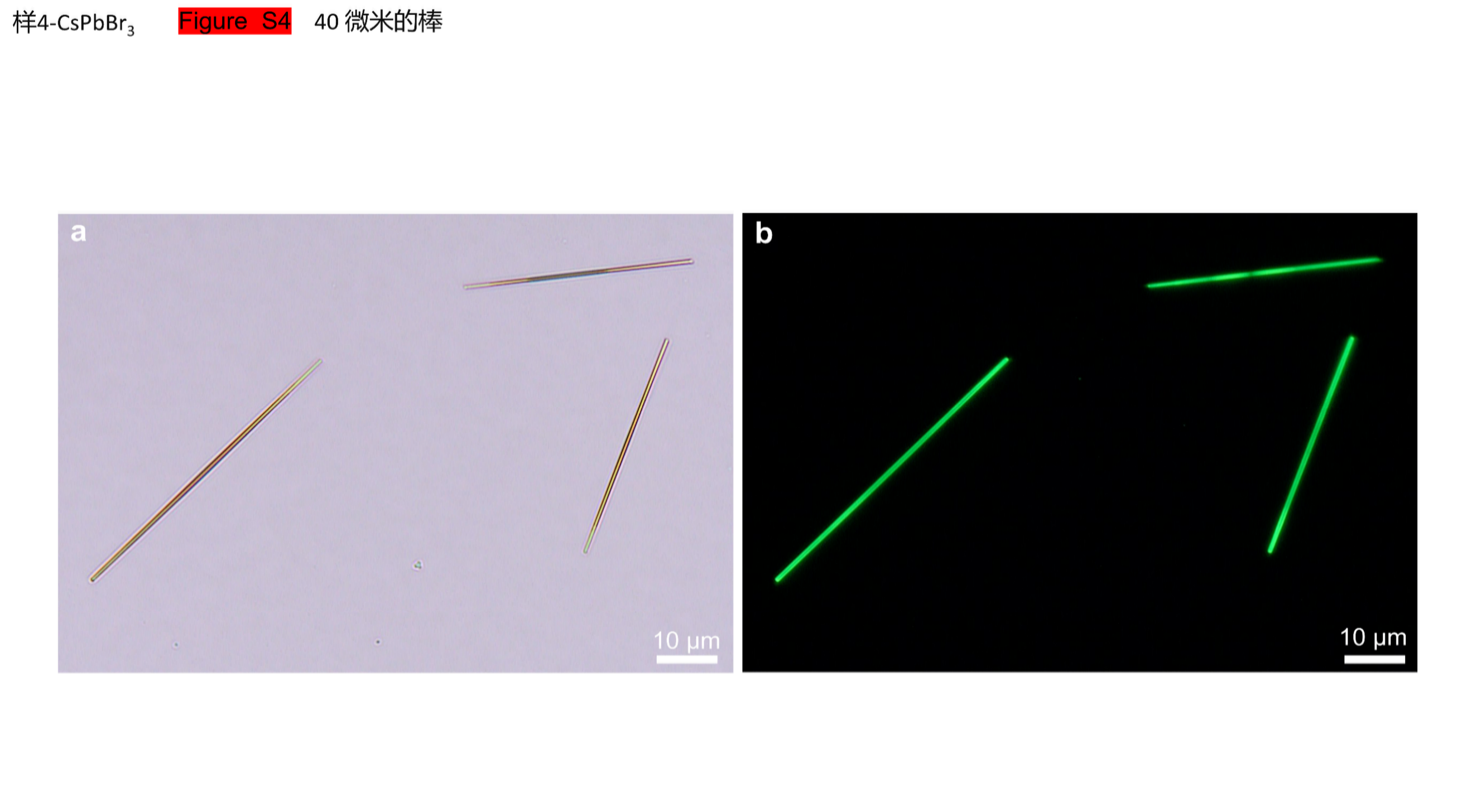


**Figure S25.** 40 μm CsPbBr_3_ NWs. (a) Optical image and (b) PL image. Excitation wavelength: 360 nm.


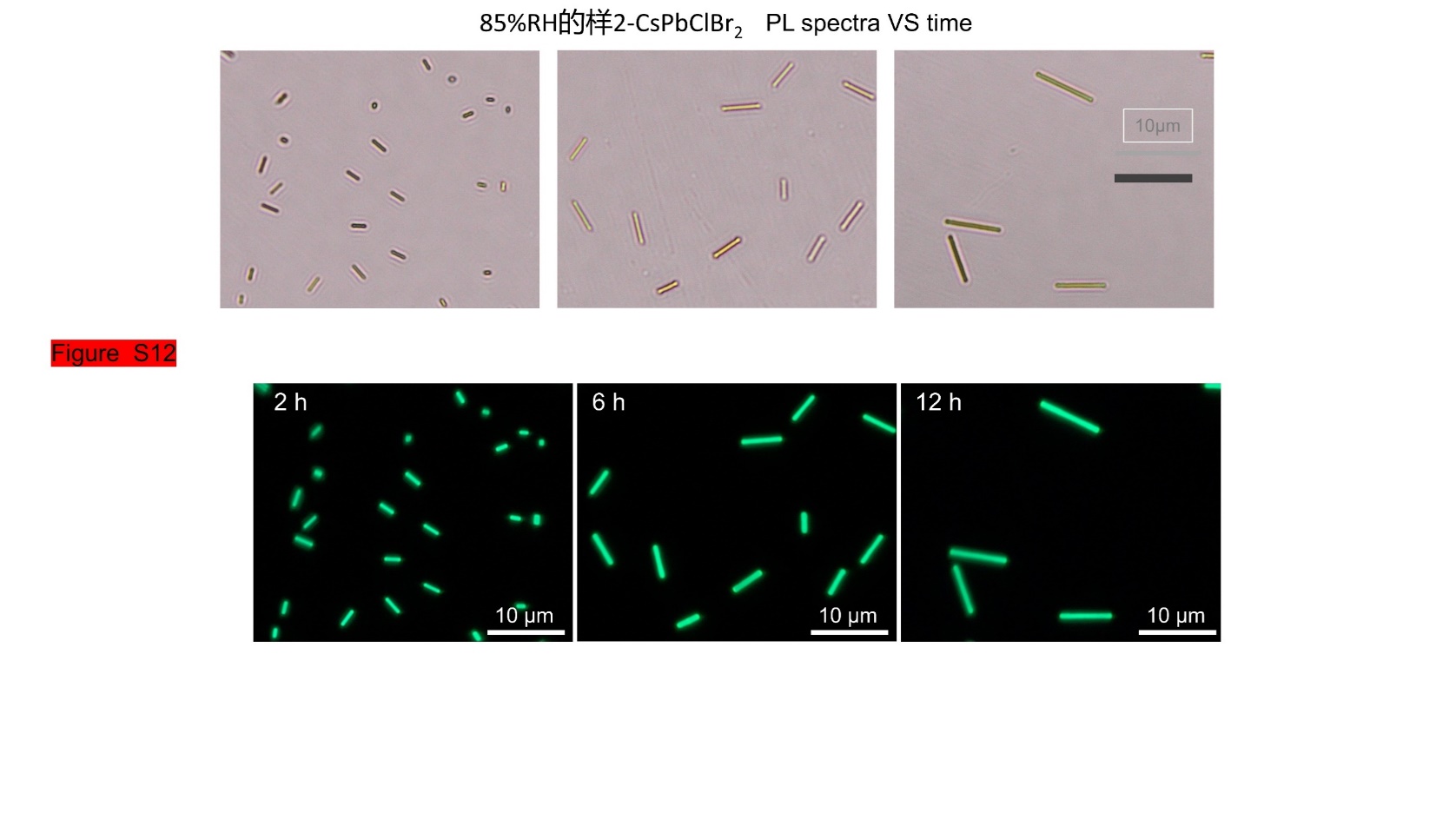


**Figure S26.** PL image of mixed-halide perovskite NWs on the glass with Cl/Br=1/2 for controlled growth times under 85% RH.


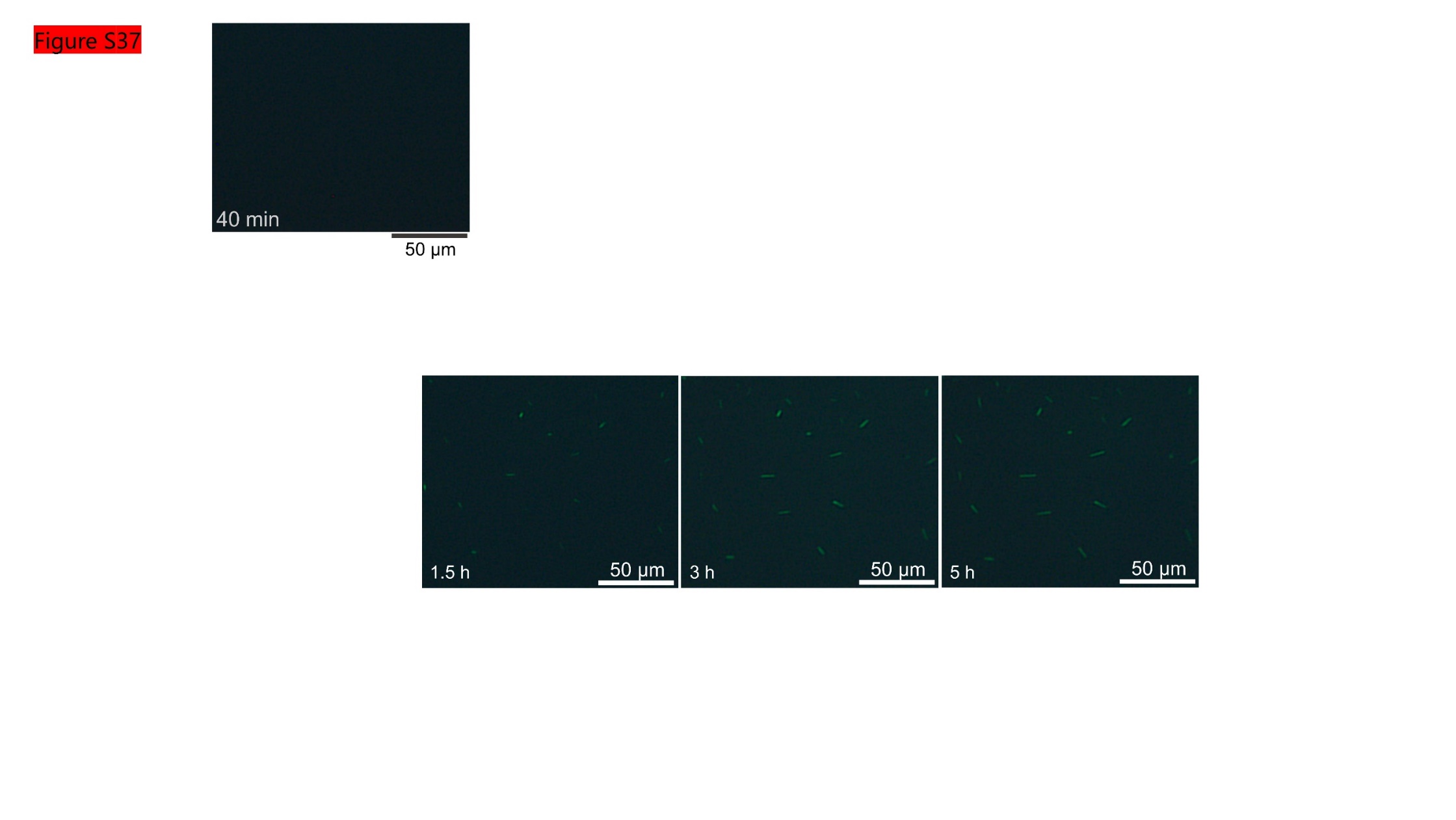


**Figure S27.** In-situ micrographs of CsPbBr_3_ NWs with time excited by UV light of 360 nm.


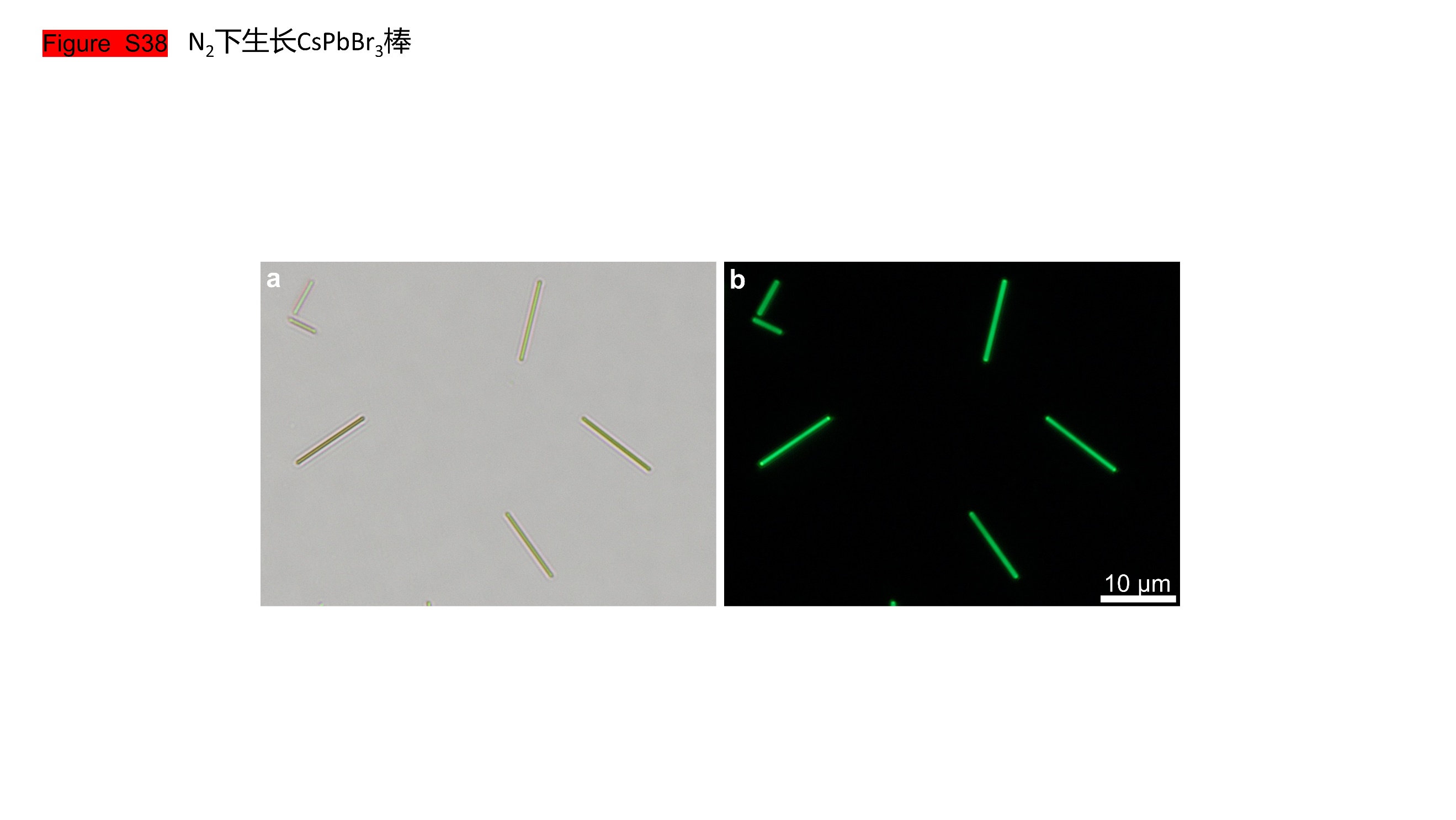


**Figure S28.** CsPbBr_3_ NWs prepared under 85% nitrogen conditions.


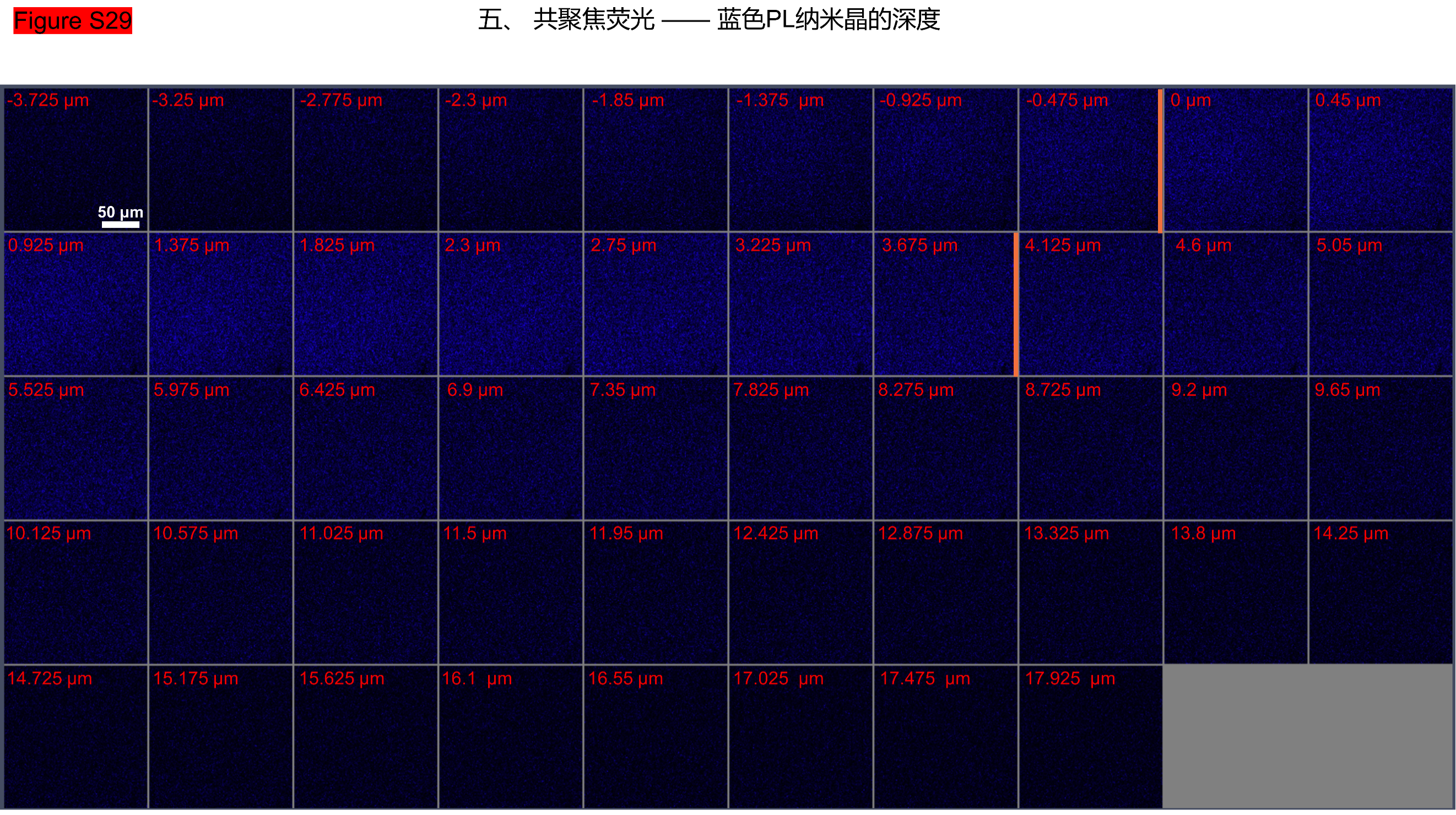


**Figure S29.** PL images of ultrasmall CsPbBr_3_ QDs via laser scanning confocal PL microscope under various focal depths. Negative and positive depth (*d*_q_) means positions above and below the surface, respectively. No distinguished dispersed structures can be identified under optical microscope. There is no PL with *d*_q_ of -2.775 μm. Blue emission becomes observable when the focus approaches the surface, and fades when *d*_q_ is larger 4.125 μm. These results illustrate that QDs locate beneath the glass surface with *d*_q_ in the range of 0-4 μm.


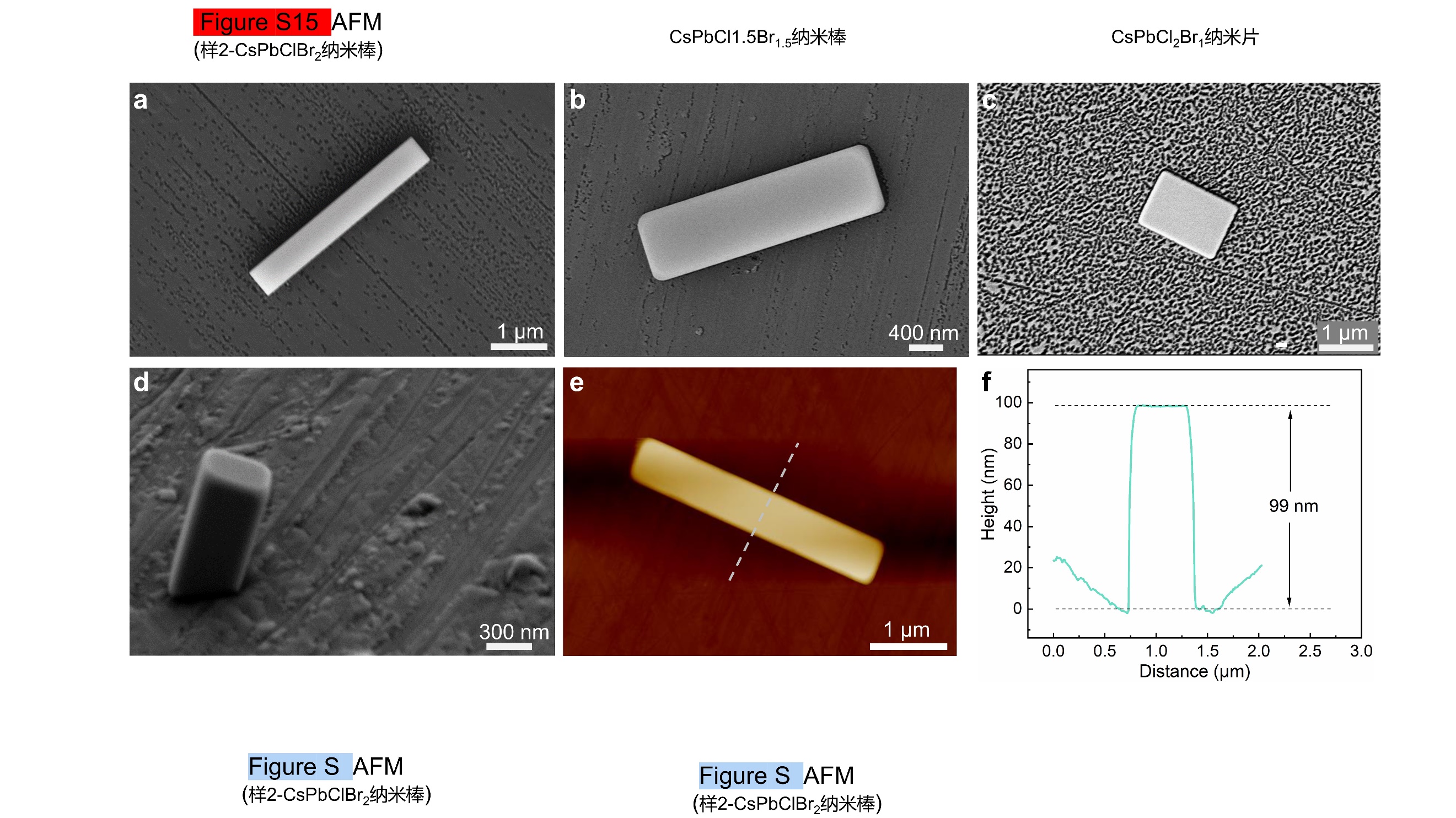


**Figure S30.** SEM images of mixed-halide perovskite NWs on the glass. (a) *R*_Cl/Br_ = 1/2. (b) *R*_Cl/Br_ = 1.5/1.5. (c) *R*_Cl/Br_ = 2/1.


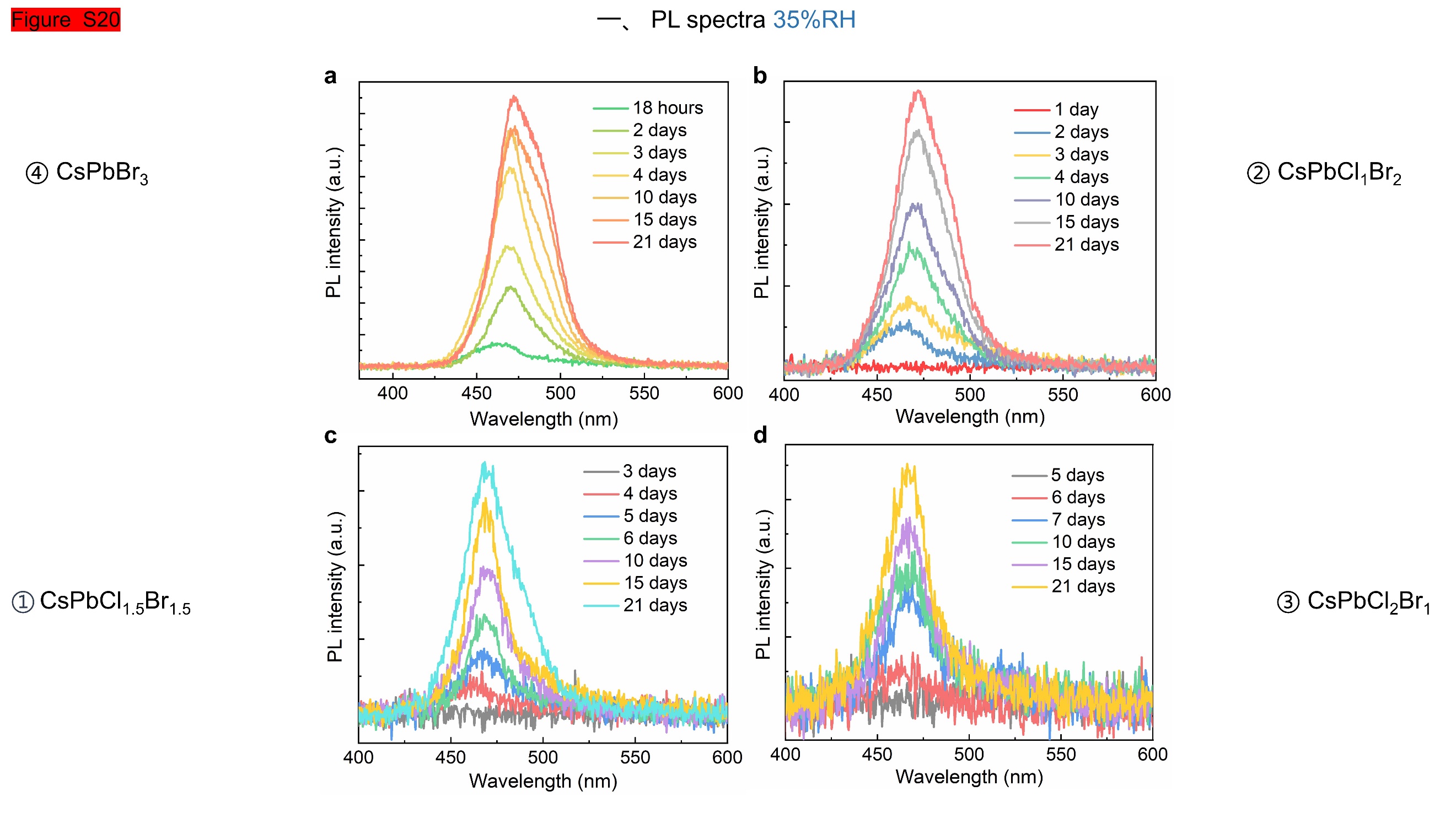


**Figure S31.** PL spectra for MHPs on various types of glass under 35% RH as a function of time. (a) *R*_Cl/Br_ = 0/3. (b) *R*_Cl/Br_ = 1/2. (c) *R*_Cl/Br_ = 1.5/1.5. (d) *R*_Cl/Br_ = 2/1.


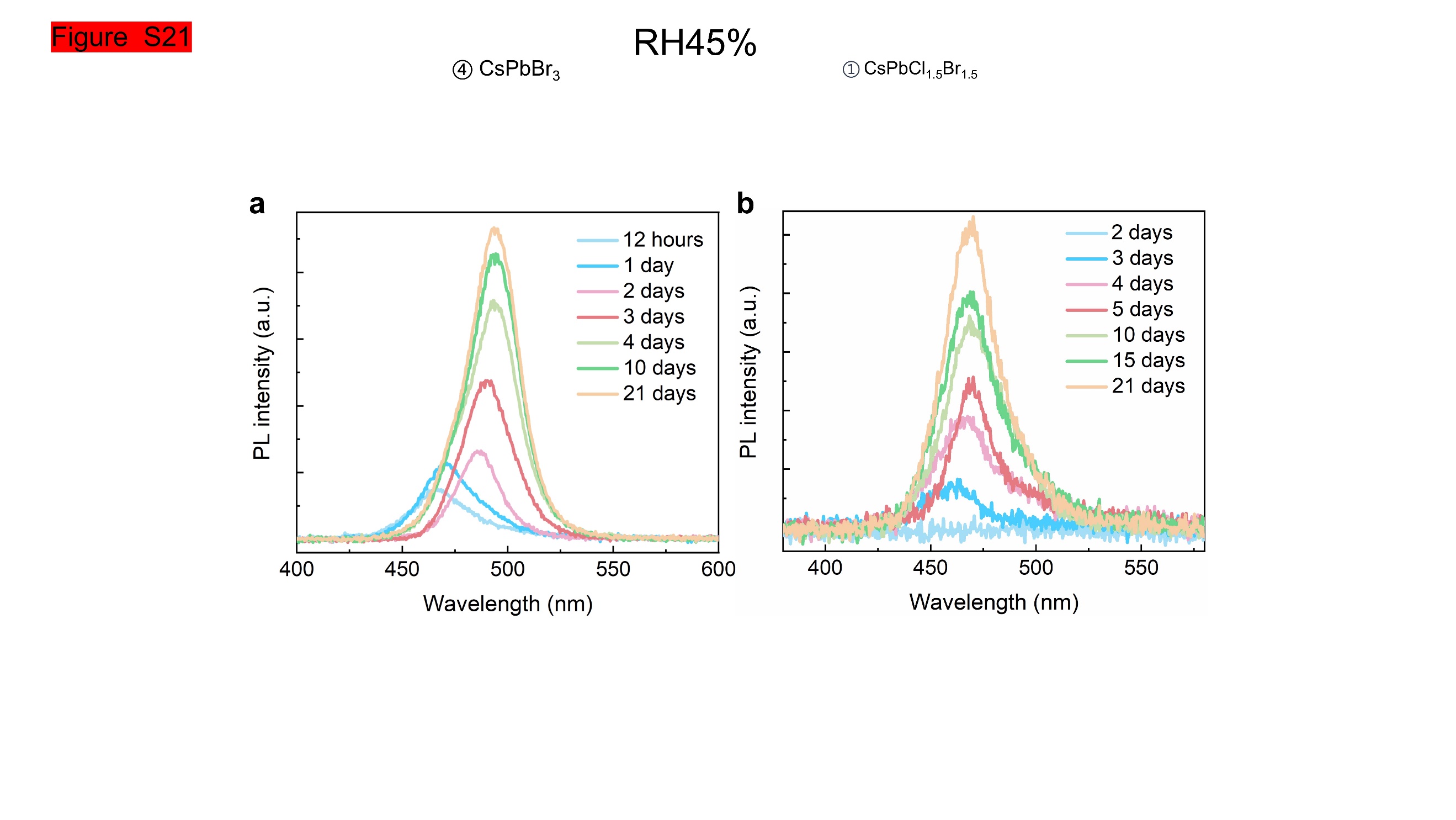


**Figure S32.** PL spectra for MHPs on glass under 45% RH as a function of time. (a) *R*_Cl/Br_ = 0/3. (b) *R*_Cl/Br_ = 1.5/1.5.

Supplementary Figure S31a and S32a illustrate the formation of ultrasmall CsPbBr_3_ quantum dots.


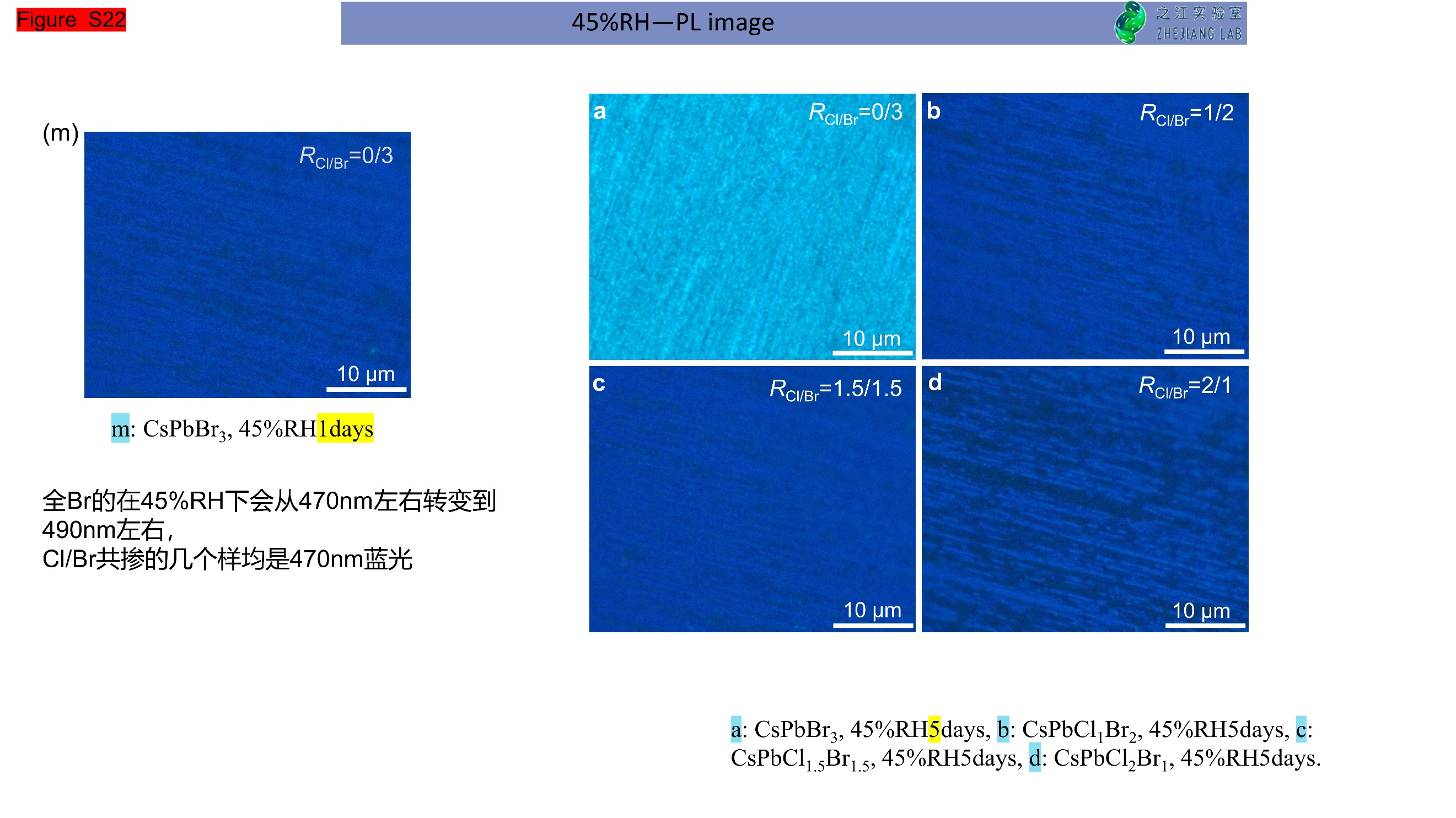


**Figure S33.** PL images excited by UV light. (a) *R*_Cl/Br_ = 0/3, 45% RH, 5 days. (b) *R*_Cl/Br_ = 1/2, 45% RH, 5 days. (c) *R*_Cl/Br_ = 1.5/1.5, 45% RH, 5 days. (d) *R*_Cl/Br_ = 2/1, 45% RH, 5 days.


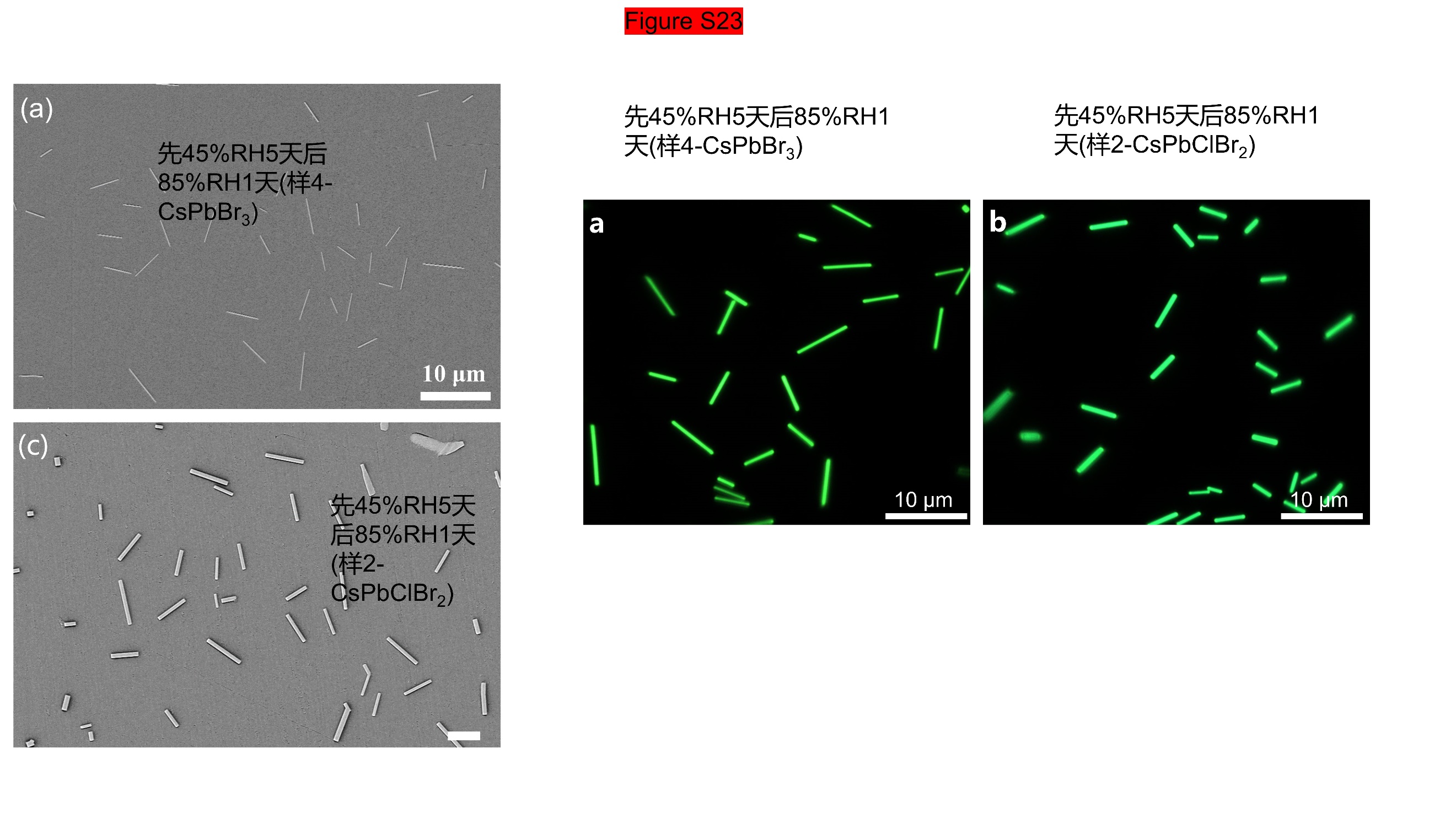


**Figure S34.** PL images of NWs with two-step humidity treatment: 45% RH, 5 days + 85% RH, 1 day. (a) *R*_Cl/Br_ = 0/3. (b) *R*_Cl/Br_ = 1/2.


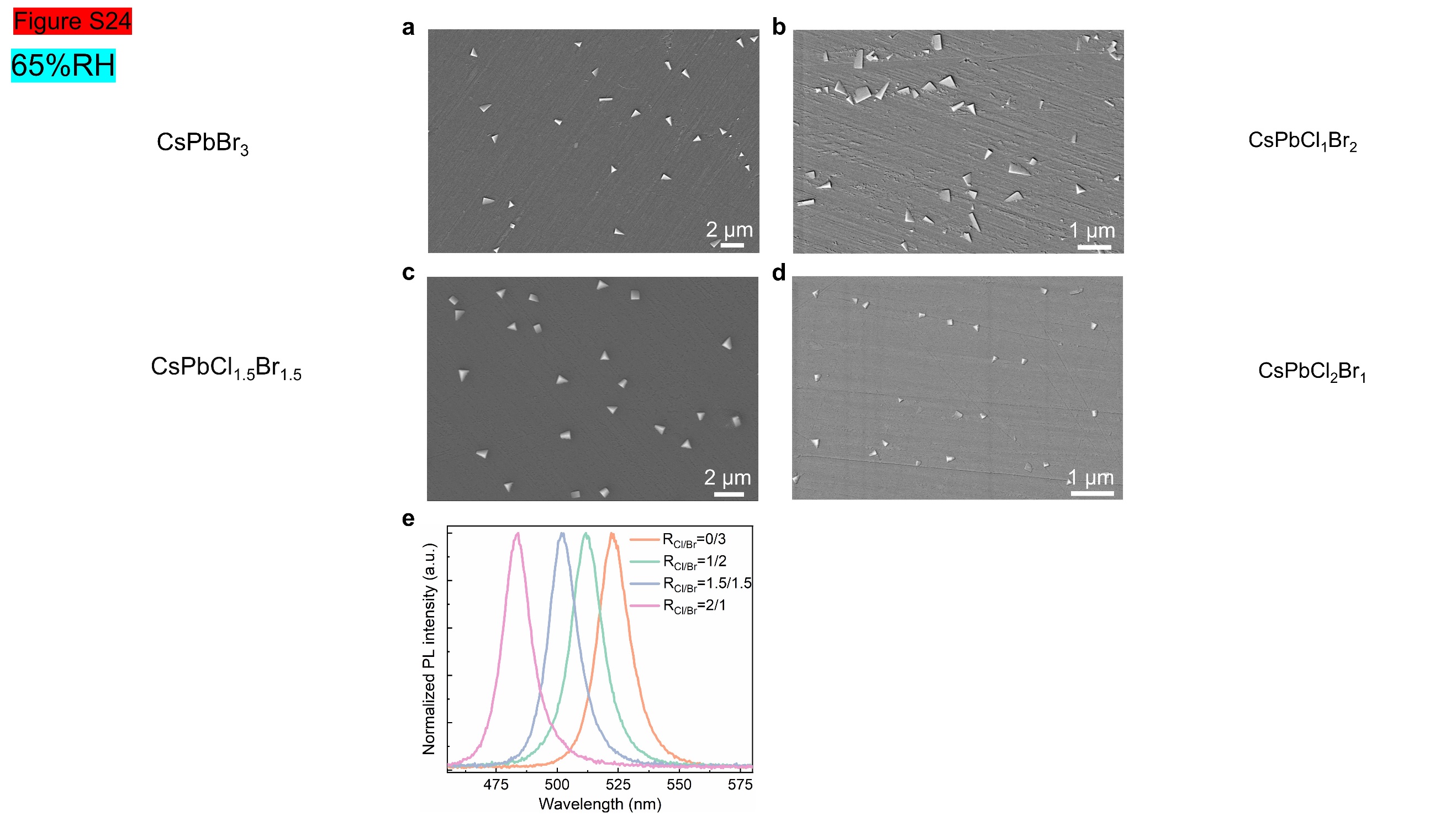


**Figure S35.** MHP tetrahedrons with tunable composition synthesized under 65% RH air on glass surfaces. SEM images of MHP tetrahedrons on the glass with (a) *R*_Cl/Br_ = 0/3, (b) *R*_Cl/Br_ = 1/2, (c) *R*_Cl/Br_ = 1.5/1.5, and (d) *R*_Cl/Br_ = 2/1. (e) PL spectra for the MHP tetrahedrons at 65% RH. Excitation: 405 nm.

As the size of tetrahedra MHPs is much larger than that of the exciton Bohr radii of CsPbBr_3_, supplementary Figure S35e indicates that CsPb(Cl*_x_*Br_1−_*_x_*)_3_ MHPs were synthesized on the glass.


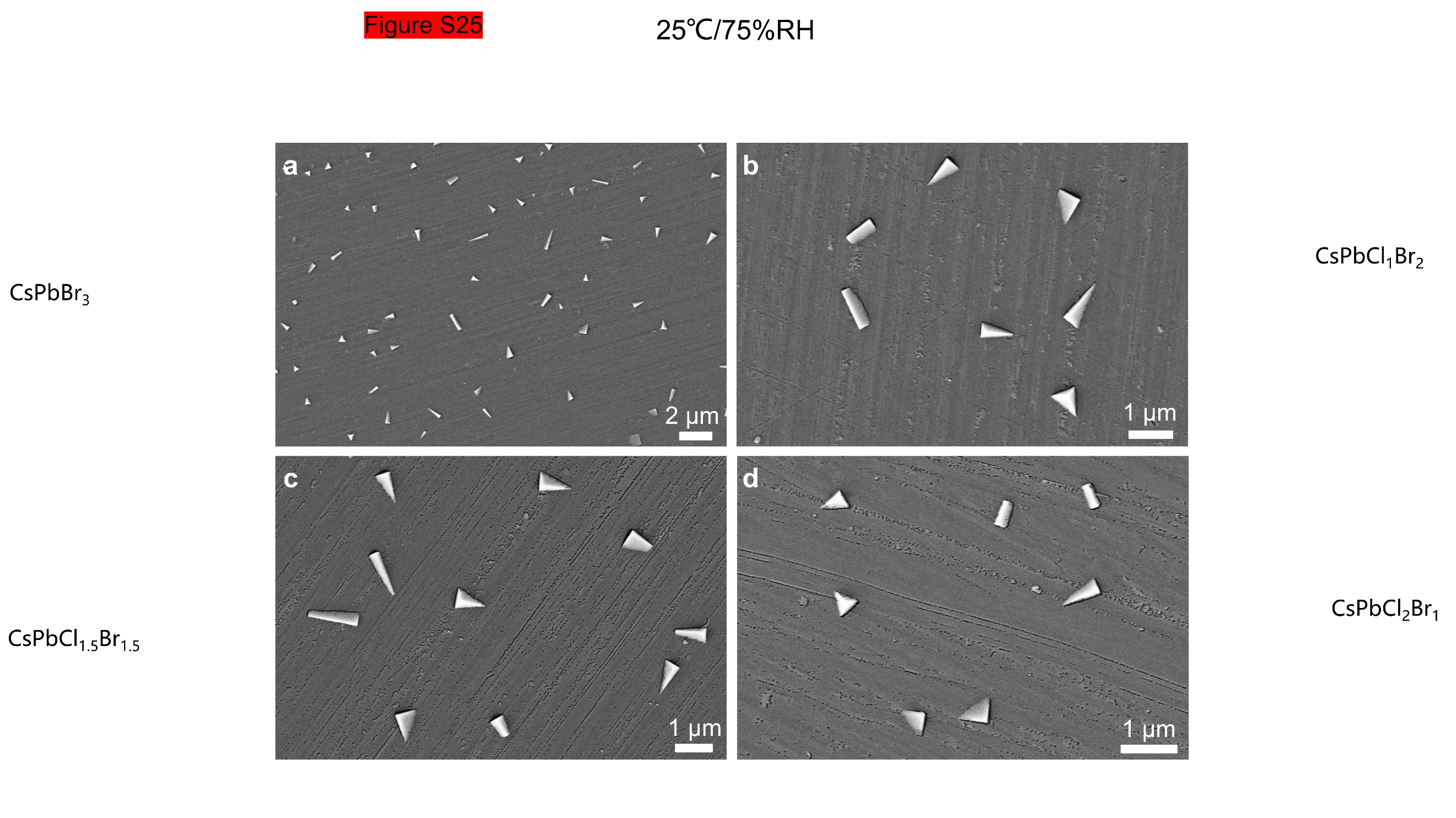


**Figure S36.** SEM images of MHP tetrahedrons synthesized on the glass. (a) *R*_Cl/Br_ = 0/3, (b) *R*_Cl/Br_ = 1/2, (c) *R*_Cl/Br_ = 1.5/1.5 and (d) *R*_Cl/Br_ = 2/1. RH: 75%.


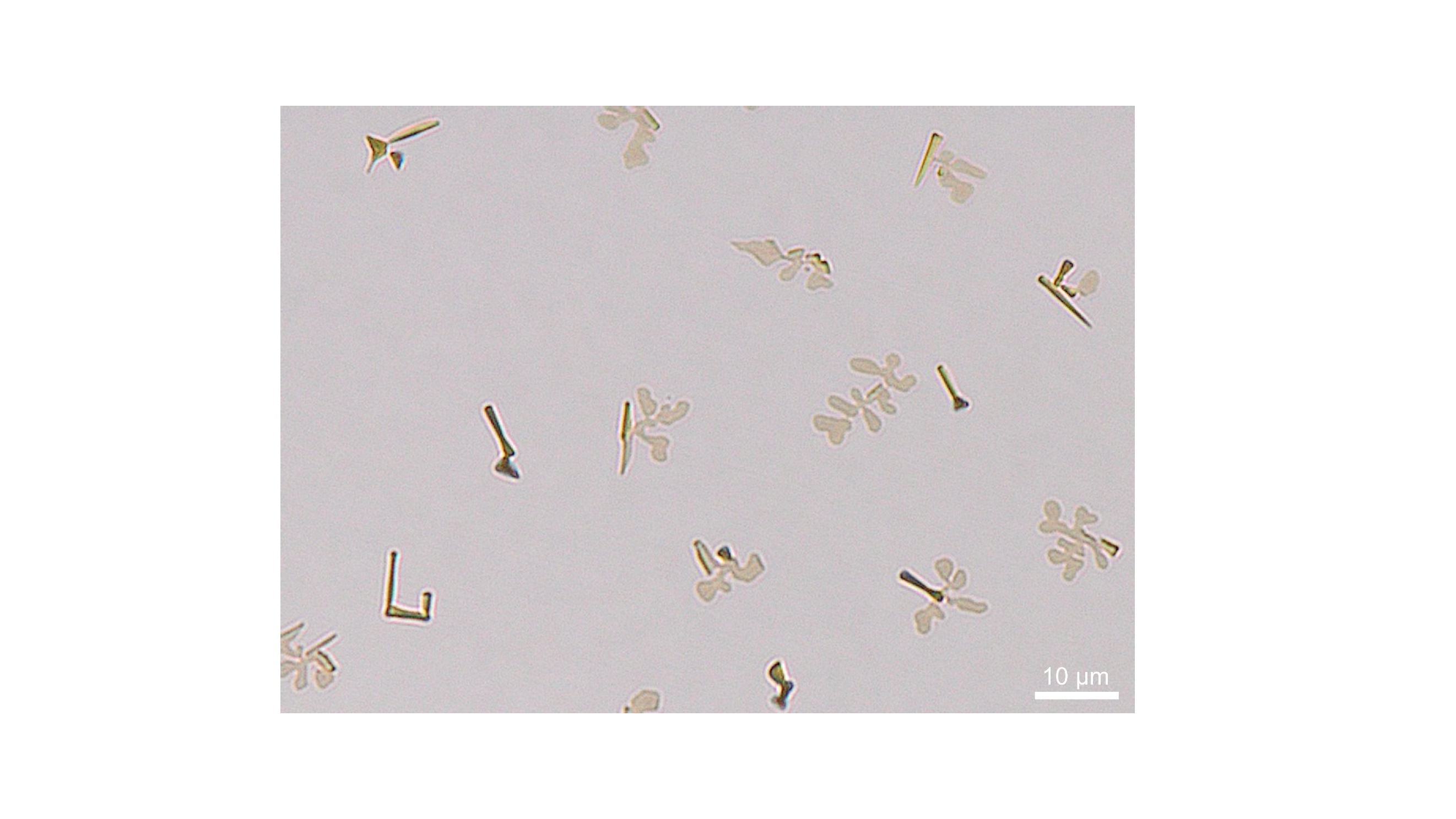


**Figure S37.** Optical microscopic image of perovskite microstructures on glass surface prepared at the relative humidity of 95%RH for 4 hours.


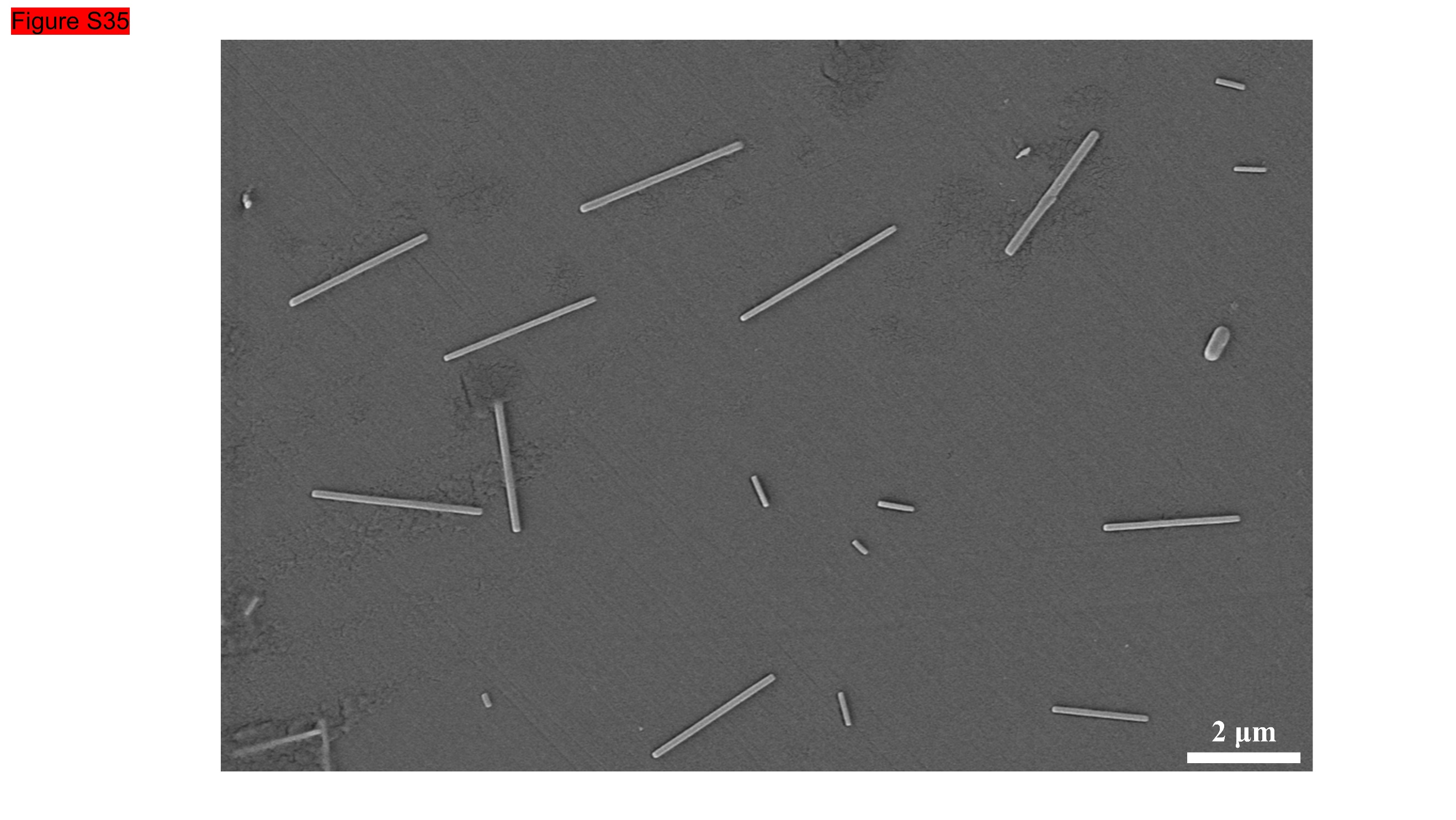


**Figure S38.** CsPbBr_3_ NWs produced by exposure the glass substrate to air in the rainy day.


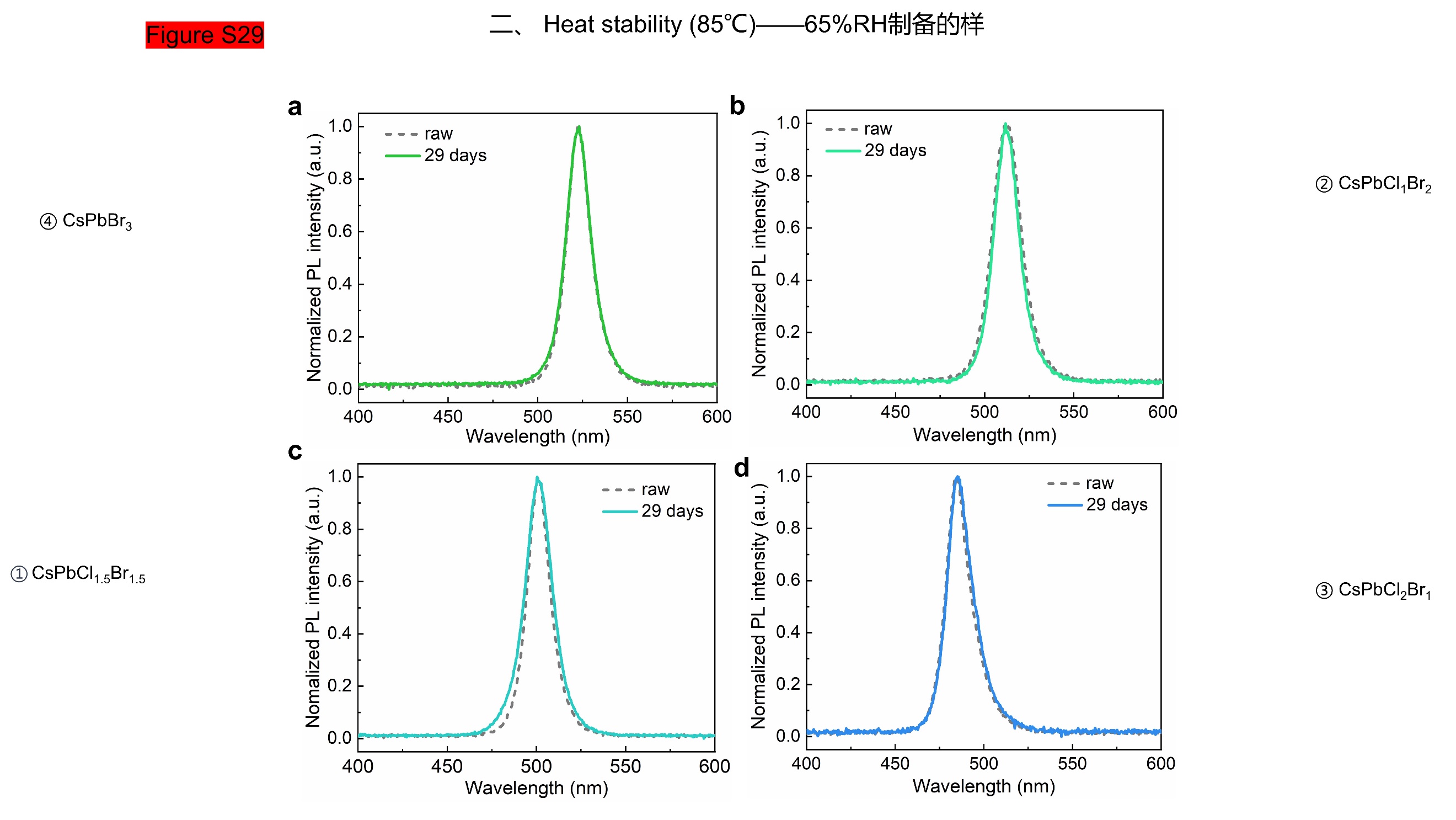


**Figure S39.** Normalized PL spectra of MHP tetrahedrons synthesized at 65% RH before and after storage under 85 ℃ and ambient conditions for 29 days. (a) *R*_Cl/Br_ = 0/3. (b) *R*_Cl/Br_ = 1/2. (c) *R*_Cl/Br_ = 1.5/1.5. (d) *R*_Cl/Br_ = 2/1.

MHP tetrahedrons also showed excellent stability when heated at 85℃ (Figure. S39).


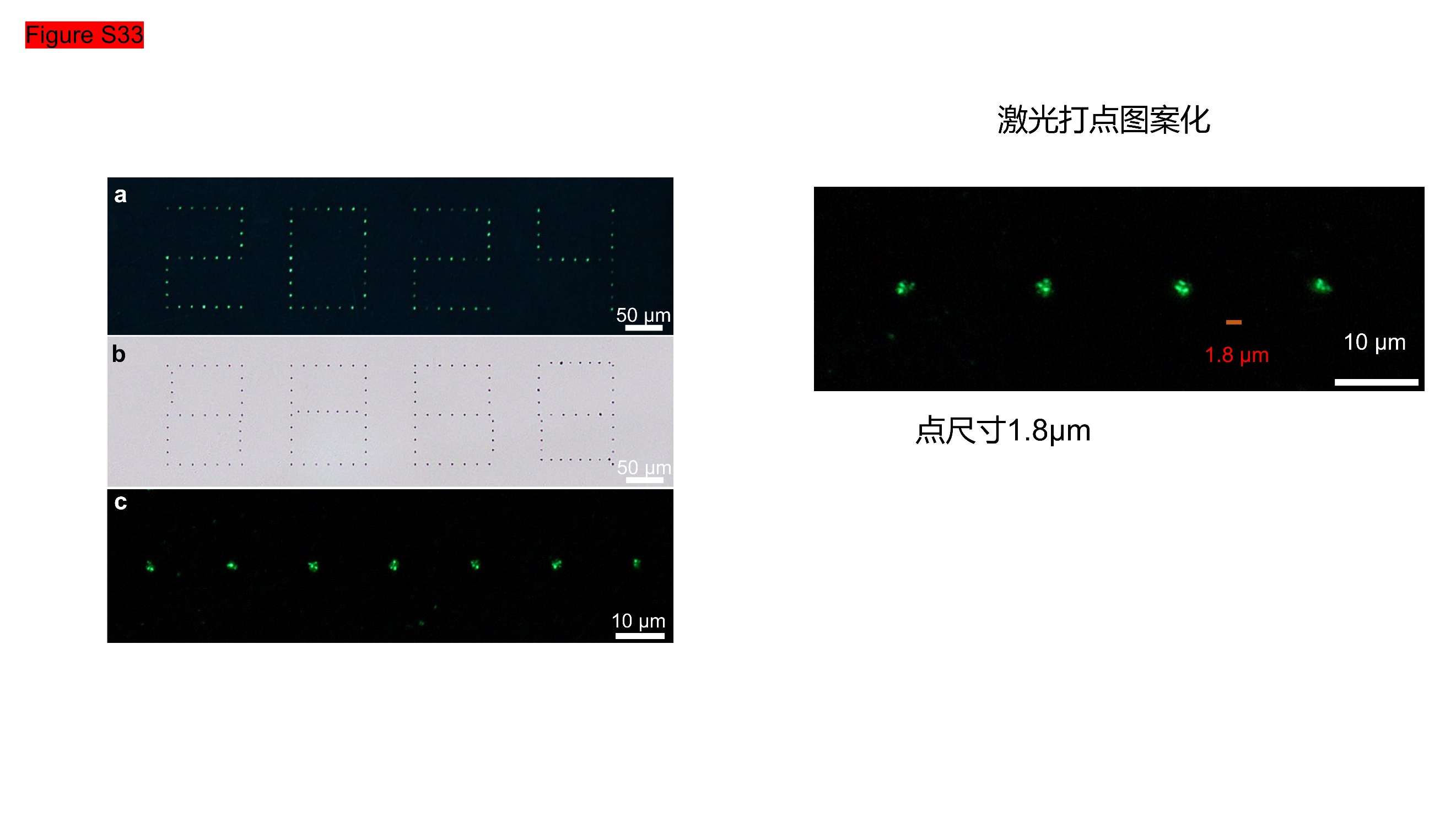


**Figure S40.** Dots created by combination of ultrafast laser direct writing and humidity treatment for optical data storage and information encryption. (a) A “2024” pattern was observed under PL microscopy and an “8888” pattern (b) was observed under optical microscopy in the same area. (c) The smallest size is 1.8 μm.

**Figure S41.** Holographic display based on blue emission excited by 405-nm holographic light.


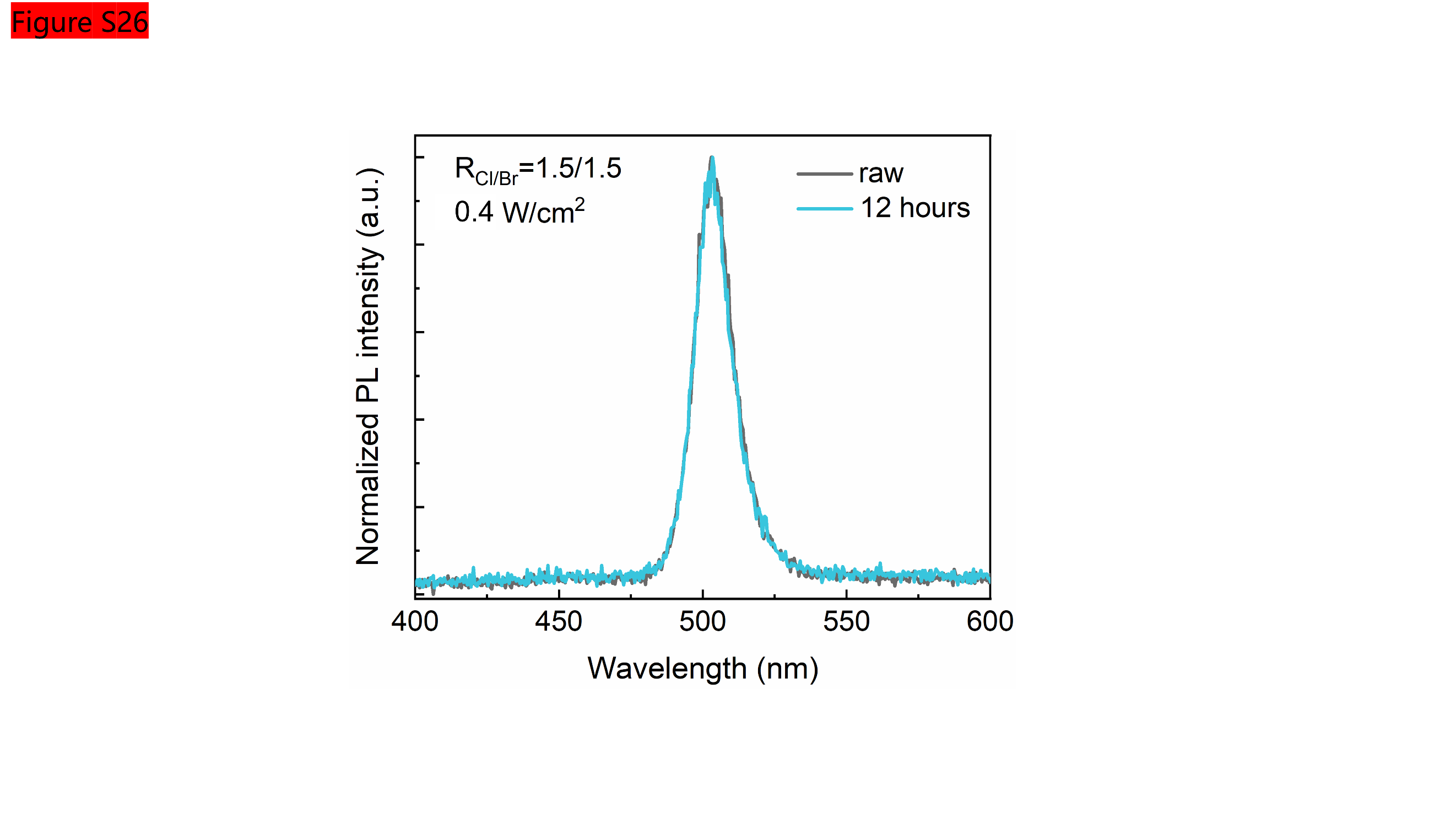


**Figure S42.** Stability of MHPs. PL spectra of CsPb(Cl*_x_*Br_1−_*_x_*)_3_ NWs before and after 405-nm continuous laser irradiation. *R*_Cl/Br_ = 1.5/1.5.


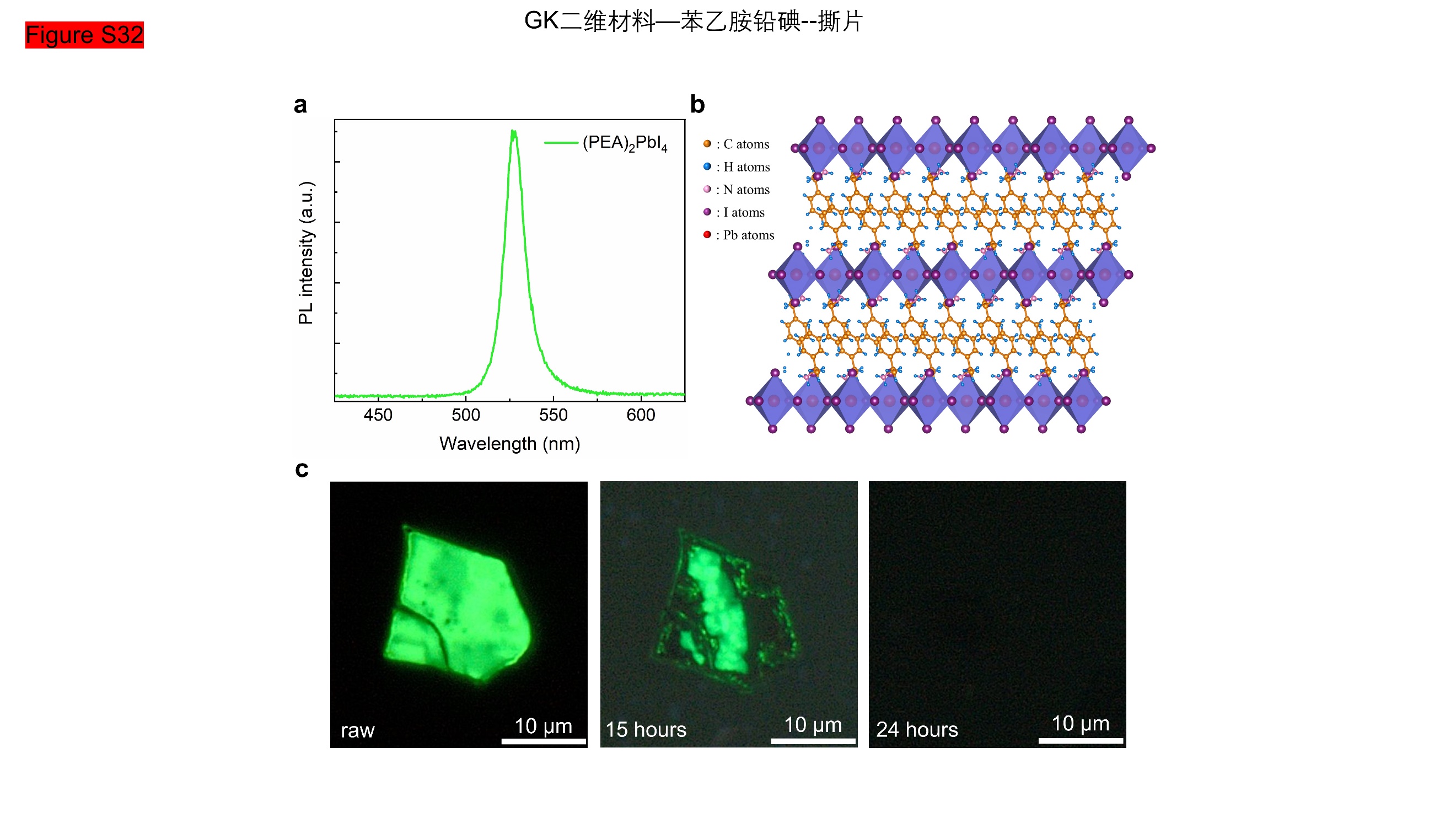


**Figure S43.** PL and crystal structure of two-dimensional layered (C_4_H_9_NH_3_)_2_PbI_4_ perovskite. (a) PL spectrum. (b) Schematic of crystal structure.


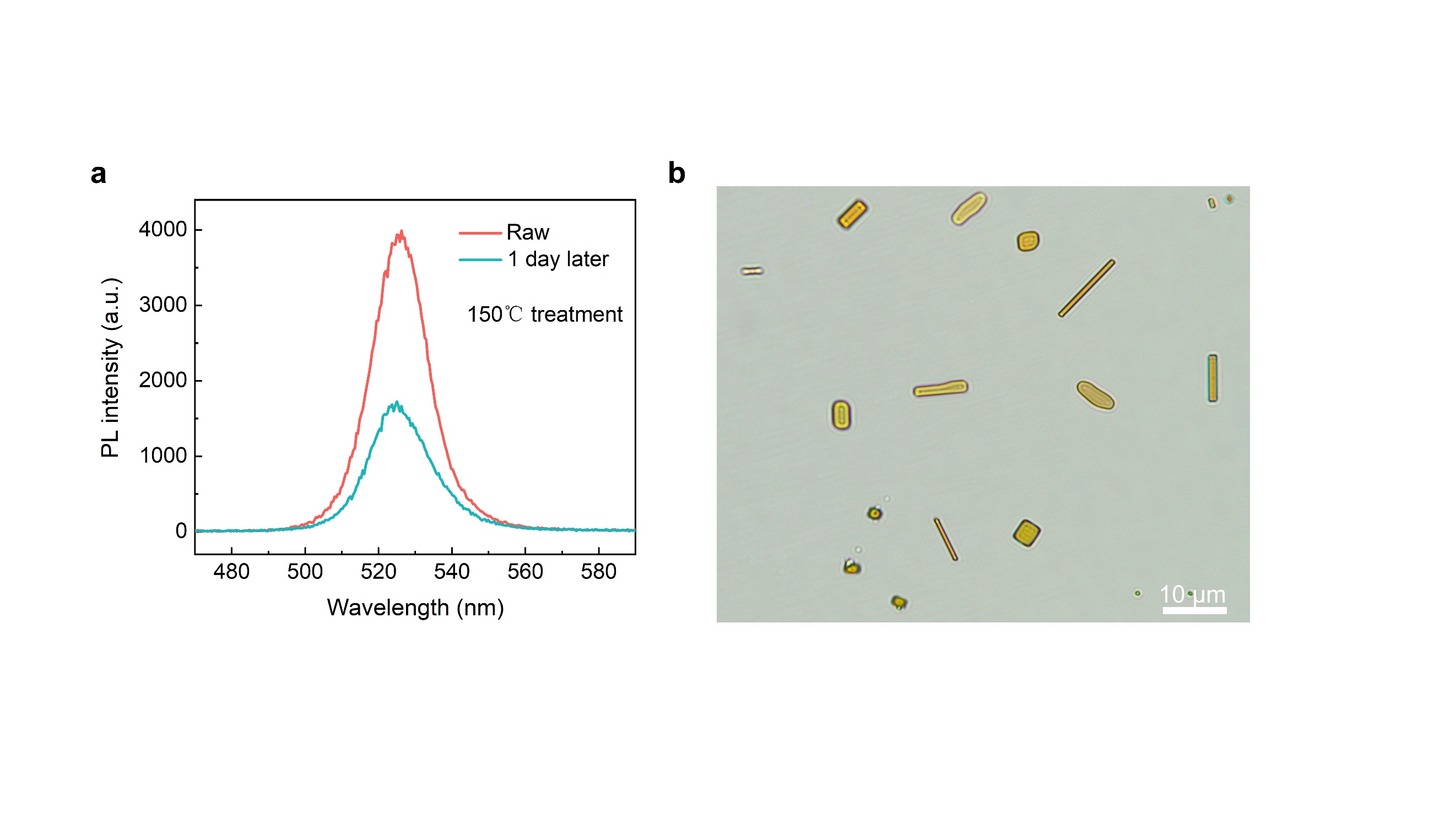


**Figure S44.** PL spectra of CsPbBr_3_ nanowires before and after 150 ℃ treatment.

**Table S1. Full width at half maximum (FWHM) of the PL spectrum, and degree of polarization (DOP) of PL of MHPs produced on various glasses with different *R*_Cl/Br_**.

| *R*_Cl/Br_ | FWHM | DOP of PL |
| --- | --- | --- |
| *R*_Cl/Br_ = 0/3 | 13.8 nm | 0.78 |
| *R*_Cl/Br_ = 1/2 | 13.7 nm | 0.87 |
| *R*_Cl/Br_ = 1.5/1.5 | 12.6 nm | 0.76 |
| *R*_Cl/Br_ = 2/1 | 14.5 nm | 0.78 |

**Table S2. Comparison for FWHM of PL spectrum, and DOP of PL, lasing, and electroluminescence (EL).**

| Materials | FWHM (nm) | DOP | Ref. |
| --- | --- | --- | --- |
| CsPbBr*_3_* nanowires | 13.8 | 0.78 (PL), 0.88 (SML) | This work |
| CH_3_NH_3_PbBr_3_ nanowires | 22 | 0.84 (PL) | [4] |
| CH_3_NH_3_PbI_3_ nanowires | 44 | 0.18 (PL), 0.82 (MML) | [5] |
| CH_3_NH_3_PbI_3_ nanowires | - | 0.7 (PL) | [6] |
| CsPbBr_3_ nanowires | - | 0.78 (PL) | [7] |
| CsPbBr_3_ nanowires | 29 | 0.71 (PL) | [8] |
| CsPbI_3_ nanorods | - | 0.63 (PL) | [9] |
| CsPbBr_3_ nanowires |  | 0.42 (PL) | [10] |
| CsPbBr_3_ nanowires | 20 | 0.1 (PL) | [11] |
| CsPbBr_1.2_I_1.8_ nanowires | 30 | 0.4 (PL) | [11] |
| CsPbBr_3_ nanorods | 56.2 | 0.4 (PL) | [12] |
| CsPbBr_3_ nanowires |  | 0.36 (PL) | [13] |
| CsPbBr_3_ nanowires | - | 0.3 (PL) | [14] |
| CsPbBr_3_ nanowires | 25 | 0.5 (PL) | [15] |
| CsPbBr_3_ nanowires |  | 0.2 (PL) | [16] |
| CsPbI_3_ nanoplatelet superlattices | - | 0.12 (PL), 0.74 (EL) | [17] |

Notes: Single mode lasing was denoted as SML and multi-mode lasing was denoted as MML. For the MML from CH_3_NH_3_PbI_3_ nanowires, one-photon excitation was used (*13*).

**Table S3. Comparison for propagation loss of active micro/nano-structured waveguides.**

| Materials | Propagation loss | Ref. |
| --- | --- | --- |
| CsPbBr_3_ nanowires | 0.018 dB/μm | This work |
| CH_3_NH_3_PbI_3_ microwires | 0.3 dB/μm | [18] |
| CH_3_NH_3_PbI_3_ microwires | 0.3 dB/μm | [19] |
| CsPbBr_3_ microsheets | 0.072 - 0.136 dB/μm | [20] |
| CdS nanoribbons | 0.026 dB/μm | [21] |
| Zinc–organic halide microcrystals | 0.062 dB/μm | [22] |
| Organic cocrystal microwires | 0.018 dB/μm | [23] |
| Organic microwires | 0.069 dB/μm | [24] |
| Organic microwires | 0.052 dB/μm | [25] |
| Organic microcrystal resonator | 0.134 dB/μm | [26] |
| Polymer nanowires | 0.48 dB/μm | [27] |
| Polydiacetylene microtube | 0.18 dB/μm | [28] |

**Table S4. Comparison for lasing threshold, FWHM of laser spectrum, quality factor (Q) and lasing mode.**

| Materials | Pump | Lateral size  (μm^3^) | Threshold  (mJ/cm^2^) | FWHM  (nm) | Q | Lasing  mode | Ref. |
| --- | --- | --- | --- | --- | --- | --- | --- |
| CsPbBr_3_ nanowires | 3PP | 10 x 0.32 x 0.078 | 4.65 | 0.15 | 3481 | SML | This work |
| CsPbBr_3_ microwires | 3PP | 200 x 20 x20 | 1.3 |  |  | SML | [29] |
| CH_3_NH_3_PbBr_3_ microplates | 3PP | 65 x 39 x 1.6 | 130 | 0.3 | 1800 | MML | [30] |
| CsPbBr_3_ microwires | 2PP | 140 x 2 x 1 | 18.89 | 0.17 | 3200 | SML | [31] |
| CsPbBr_3_ nanoorods | 2PP  3PP | 10 x 0.6 x 0.5 | 0.6  1.7 | 0.22 | 2450 | MML  MML | [32] |
| CH_3_NH_3_PbBr_3_ microwires | 2PP | 18.64 x 0.84 x 0.625 | 0.67 | 0.8 | 682 | MML | [33] |
| CsPbBr_3_ microrods | 2PP | 8.7 x 0.58 x 0.75 | 20.1 | 0.13 | 4200 | MML | [34] |
| CsPbBr_3_ QDs | 2PP |  | 0.9 | 0.5 | 1070 | MML | [35] |
| CsPbBr_3_ QDs | 2PP  3PP |  | 2.5  5.2 |  |  | ASE | [36] |
| CsPbBr_3_ NCs 2D photonic supercrystals | 2PP |  | 10.9 |  |  | ASE | [37] |
| CsPbBr_3_ QD film | 2PP |  | 12 |  |  | ASE | [38] |
| CH_3_NH_3_PbI_3_ single crystal | 3PP |  | 5.2 |  |  | ASE | [39] |
| CsPbBr_3_ single Crystals | 2PP |  | 0.65 |  |  | ASE | [40] |
| MAPbBr_3_ film | 2PP |  | 1.19 | 4.82 | 682 | ASE | [41] |
| CdSe/CdS nanoplatelets | 2PP  3PP |  | 1.2  4.3 | 0.54 | 1200 | MML | [42] |
| Metal-organic framework crystal | 3PP |  | 29.5 | 0.38 | 1691 | MML | [43] |

Notes: 2PP means two-photon pumped. 3PP means three-photon pumped. QDs means Quantum dots: NCs means nanocrystals. ASE means amplified spontaneous emission.

**Table S5. Wavelength, FWHM, and DOP of lasing, ratio of side-mode suppression (R_SMS)_, quality factor (Q) of lasing** **for MHP nanostructures synthesized on glass with various *R*_Cl/Br_**.

| *R*_Cl/Br_ | Lasing wavelength | FWHM | DOP | R_SMS_ | Q |
| --- | --- | --- | --- | --- | --- |
| 0/3 | 532.8 nm | 0.15 | 0.88 | 12 dB | 3481 |
| 1/2 | 520.6 nm | 0.17 | 0.83 | 12 dB | 3137 |
| 1.5/1.5 | 507.9 nm | 0.15 | 0.86 | 11 dB | 3341 |
| 2/1 | 489 nm | 0.12 | 0.63 | 9.8 dB | 4075 |

**References**

[1] G. Kresse, J. Furthmuller, *Comput. Mat. Sci*. **1996**, *6*, 15.

[2] G. Kresse, J. Furthmuller, Phys. Rev. B **1996**, *54*, 11169.

[3] K. Yamamoto, S. Iikubo, J. Yamasaki, Y. Ogomi, S. Hayase, *J. Phys. Chem. C*, **2017**, *121*, 27797.

[4] C.-H. Lin, C.-Y. Kang, T.-Z. Wu, C.-L. Tsai, C.-W. Sher, X. Guan, P.-T. Lee, T. Wu, C.-H. Ho, H.-C. Kuo, J.-H. He, *Adv. Funct. Mater.* **2020**, *30*, 1909275.

[5] Zhu, H. Fu, Y. Meng, F. Wu, X. Gong, Z. Ding, Q. Gustafsson, M. V. Trinh, M. T. Jin, S. Zhu, X.-Y, *Nat. Mater.* **2015**, *14*, 636.

[6] D. Tauber, A. Dobrovolsky, R. Camacho, I. G. Scheblykin, *Nano Lett*. **2016**, *16*, 5087.

[7] Y. Gao, L. Zhao, Q. Shang, Y. Zhong, Z. Liu, J. Chen, Z. Zhang, J. Shi, W. Du, Y. Zhang, S. Chen, P. Gao, X. Liu, X. Wang, Q. Zhang, *Adv. Mater*. **2018**, *30*, 1801805.

[8] N. Zhou, Y. Bekenstein, C. N. Eisler, D. Zhang, A. M. Schwartzberg, P. Yang, A. P. Alivisatos, J. A. Lewis, *Sci. Adv*. **2019**, *5*, eaav8141.

[9] W. Shen, Y. Dai, B. Cai, S. Chen, H. Yang, Y. Ma, Y. Chen, Z. Su, J. Zhang, Y. Qiu, Y. Wang, J. Jiang, L. Liu, K. Cao, S. Chen, *ACS Energy Lett*. **2023**, *8*, 2561.

[10] J. Wang, Y. Zhang, J. Chen, Y. Wei, D. Yu, L. Liang, Y. Liu, Y. Wu, W. Shen, X. Li, H. Zeng, *ACS Appl. Mater. Interfaces* **2021**, *13*, 36147.

[11] Y. Dou, F. Cao, T. Dudka, Y. Li, S. Wang, C. Zhang, Y. Gao, X. Yang, A. L. Rogach, *ACS Mater. Lett.* **2020**, *2*, 814.

[12] M. Ng, S. B. Shivarudraiah, J. E. Halpert, *J. Mater. Chem. C* **2022***, 10*, 8947.

[13] Y. Tong, B. J. Bohn, E. Bladt, K. Wang, P. Mgller-Buschbaum, S. Bals, A. S. Urban, L. Polavarapu, J. Feldmann, *Angew. Chem. Int. Ed*. **2017**, *56*, 13887.

[14] S. Liang, M. Zhang, S. He, M. Tian, W. Choi, T. Lian, Z. Lin, *Nat. Synth*. **2023**, *2*, 719.

[15] Y. Wei, Y. Xu, Q. Wang, J. Wang, H. Lu, J. Zhu, *Chem. Commun*. **2020**, *56*, 5413.

[16] Y. Li, H. Huang, Y. Xiong, A. F. Richter, S. V. Kershaw, J. Feldmann, A. L. Rogach, *ACS Nano* **2019**, *13*, 8237.

[17] J. Ye, A. Ren, L. Dai, T. K. Baikie, R. Guo, D. Pal, S. Gorgon, J. E. Heger, J. Huang, Y. Sun, R. Arul, G. Grimaldi, K. Zhang, J. Shamsi, Y.-T. Huang, H. Wang, J. Wu, A. F. Koenderink, L. T. Murciano, M. Schwartzkopf, S. V. Roth, P. Müller-Buschbaum, J. J. Baumberg, S. D. Stranks, N. C. Greenham, L. Polavarapu, W. Zhang, A. Rao, R. L. Z. Hoye, *Nat. Photonics* **2024**, *18*, 586.

[18] Z. Wang, J. Liu, Z.-Q. Xu, Y. Xue, L. Jiang, J. Song, F. Huang, Y. Wang, Y. L. Zhong, Y. Zhang, Y.-B. Cheng, Q. Bao, *Nanoscale* **2016**, *8*, 6258.

[19] W. Mao, J. Zheng, Y. Zhang, A. S. R. Chesman, Q. Ou, J. Hicks, F. Li, Z. Wang, B. Graystone, T. D. M. Bell, M. Uller Rothmann, N. W. Duffy, L. Spiccia, Y.-B. Cheng, Q. Bao, U. Bach, *Angew. Chem. Int. Ed*. **2017**, *56*, 12486.

[20] Z. Li, F. Sun, Z. Zheng, J. Chen, A. V. Davydov, S. Deng, H. Zhang, H. Chen, F. Liu, *Nano Lett*. **2021**, *21*, 1822.

[21] A. Pan, D. Liu, R. Liu, F. Wang, X. Zhu, B. Zou, *Small* **2005**, *1*, 980.

[22] B. Zhou, D. Yan, *Chem. Sci*. **2022**, *13*,7429.

[23] X. Ye, Y. Liu, Q. Guo, Q. Han, C. Ge, S. Cui, L. Zhang, X. Tao, *Nat. Commun*. **2019**, *10*, 761.

[24] W. Yao, Y. Yan, L. Xue, C. Zhang, G. Li, Q. Zheng, Y. S. Zhao, H. Jiang, J. Yao, *Angew. Chem. Int. Ed*. **2013**, *52*, 8713.

[25] M. Rohullah, V. K. Avulu, R. Chandrasekar, *Laser Photonics Rev*. **2024**, *18*, 2400020.

[26] T. Matsuo, J. Kuwabara, T. Kanbara, S Hayashi, *J. Phys. Chem. Lett*. **2023**, *14*, 6577.

[27] D. O'Carroll, I. Lieberwirth, G. Redmond, *Small* **2007**, *3*, 1178.

[28] Hu, W. Chen, Y. Jiang, H. Li, J. Zou, G. Zhang, Q. Zhang, D. Wang, P. Ming, H, *Adv. Mater.* **2014**, *26*, 3136.

[29] G. Weng, J. Yan, S. Chen, C. Zhao, H. Zhang, J. Tian, Y. Liu, X. Hu, J. Tao, S. Chen, Z. Zhu, H. Akiyama, J. Chu, *Photonics Res.* **2021**, *9*, 01000054.

[30] Y. Gao, S. Wang, C. Huang, N. Yi, K. Wang, S. Xiao, Q. Song, *Sci. Rep*. **2017**, *7*, 45391.

[31] J. Lu, X. He, J. Xu, F. Li, Q. Tang, X. Wang, J. Dai, Q. Yao, F. Qin, C. Xu, *Adv. Funct. Mater*. **2024**, *34*, 2308957.

[32] X. Wang, H. Zhou, S. Yuan, W. Zheng, Y. Jiang, X. Zhuang, H. Liu, Q. Zhang, X. Zhu, X. Wang, A. Pan, *Nano Res*. **2017**, *10*, 3385.

[33] Z. Gu, K. Wang, W. Sun, J. Li, S. Liu, Q. Song, S. Xiao, *Adv. Optical Mater.* **2016**, *4*, 472.

[34] S. Wang, K. Wang, Z. Gu, Y. Wang, C. Huang, N. Yi, S. Xiao, Q. Song, *Adv. Optical Mater*. **2017**, *5*, 1700023.

[35] S. Li, D. Lei, W. Ren, X. Guo, M. Chhowalla, A. K.-Y. Jen, *Nat. Commun*. **2020**, *11*, 1192.

[36] Y. Wang, X. Li, X. Zhao, L. Xiao, H. Zeng, H. Sun, *Nano Lett.* **2016**, *16*, 448.

[37] D. Vila-Liarte, M. W. Feil, A. Manzi, J. L. Garcia-Pomar, H. Huang, M. Döblinger, L. M Liz-Marzán, J. Feldmann, L. Polavarapu, A. Mihi, *Angew. Chem. Int. Ed.* **2020**, *59*, 17750.

[38] J. Pan, S. P. Sarmah, B. Murali, I. Dursun, W. Peng, M. R. Parida, J. Liu, L. Sinatra, N. Alyami, C. Zhao, E. Alarousu, T. K. Ng, B. S. Ooi, O. M. Bakr, O. F. Mohammed, *J. Phys. Chem. Lett.* **2015**, *6*, 5027.

[39] D. Yang, C. Xie, J. Sun, H. Zhu, X. Xu, P. You, S. P. Lau, F. Yan, S. F. Yu, *Adv. Optical Mater*. **2016**, *4*, 1053.

[40] C. Zhao, W. Tian, J. Liu, Q. Sun, J. Luo, H. Yuan, B. Gai, J. Tang, J. Guo, S. Jin, *J. Phys. Chem. Lett*. **2019**, *10*, 2357.

[41] T. Shen, J. Qin, Y. Bai, J. Zhang, L. Shi, X. Hou, J. Zi, B. Hu, *Opto-Electron Adv* **2022**, *5*, 200051.

[42] M. Li, M. Zhi, H. Zhu, W.-Y. Wu, Q.-H. Xu, M. H. Jhon, Y. Chan, *Nat. Commun*. **2015**, *6*, 8513.

[43] Q. Zheng, H. Zhu, S.-C. Chen, C. Tang, E. Ma, X. Chen, *Nat. Photonics* **2013**, *7*, 234.
